# Enhanced Solid-State Fluorescence of Flavin Derivatives by Incorporation in the Metal-Organic Frameworks MIL-53(Al) and MOF-5

**DOI:** 10.3390/molecules28062877

**Published:** 2023-03-22

**Authors:** Dietrich Püschel, Simon Hédé, Iván Maisuls, Simon-Patrick Höfert, Dennis Woschko, Ralf Kühnemuth, Suren Felekyan, Claus A. M. Seidel, Constantin Czekelius, Oliver Weingart, Cristian A. Strassert, Christoph Janiak

**Affiliations:** 1 Institut für Anorganische Chemie und Strukturchemie, Heinrich-Heine-Universität Düsseldorf, Universitätsstraße 1, D-40225 Düsseldorf, Germany; 2 Institut für Theoretische Chemie und Computerchemie, Heinrich-Heine-Universität Düsseldorf, Universitätsstraße 1, D-40225 Düsseldorf, Germany; 3 Institut für Anorganische und Analytische Chemie, CeNTech, CiMIC, SoN, Westfälische Wilhelms-Universität Münster, Heisenbergstraße 11, D-48149 Münster, Germany; 4 Institut für Physikalische Chemie, Heinrich-Heine-Universität Düsseldorf, Universitätsstraße 1, D-40225 Düsseldorf, Germany; 5 Institut für Organische Chemie und Makromolekulare Chemie, Heinrich-Heine-Universität Düsseldorf, Universitätsstraße 1, D-40225 Düsseldorf, Germany

**Keywords:** flavins, 10-methyl-isoalloxazine, metal-organic framework (MOF), MIL-53, MOF-5, fluorescence, flavin@MOF, solid solution, dye anisotropy, fluorescence lifetime, multiparametric fluorescence microscopy

## Abstract

The flavin derivatives 10-methyl-isoalloxazine (MIA) and 6-fluoro-10-methyl-isoalloxazine (6F-MIA) were incorporated in two alternative metal-organic frameworks, (MOFs) MIL-53(Al) and MOF-5. We used a post-synthetic, diffusion-based incorporation into microcrystalline MIL-53 powders with one-dimensional (1D) pores and an in-situ approach during the synthesis of MOF-5 with its 3D channel network. The maximum amount of flavin dye incorporation is 3.9 wt% for MIA@MIL-53(Al) and 1.5 wt% for 6F-MIA@MIL-53(Al), 0.85 wt% for MIA@MOF-5 and 5.2 wt% for 6F-MIA@MOF-5. For the high incorporation yields the probability to have more than one dye molecule in a pore volume is significant. As compared to the flavins in solution, the fluorescence spectrum of these flavin@MOF composites is broadened at the bathocromic side especially for MIA. Time-resolved spectroscopy showed that multi-exponential fluorescence lifetimes were needed to describe the decays. The fluorescence-weighted lifetime of flavin@MOF of 4 ± 1 ns also corresponds to those in solution but is significantly prolonged compared to the solid flavin dyes with less than 1 ns, thereby confirming the concept of “solid solutions” for dye@MOF composites. The fluorescence quantum yield (*Φ*_F_) of the flavin@MOF composites is about half of the solution but is significantly higher compared to the solid flavin dyes. Both the fluorescence lifetime and quantum yield of flavin@MOF decrease with the flavin loading in MIL-53 due to the formation of various J-aggregates. Theoretical calculations using plane-wave and QM/MM methods are in good correspondence with the experimental results and explain the electronic structures as well as the photophysical properties of crystalline MIA and the flavin@MOF composites. In the solid flavins, π-stacking interactions of the molecules lead to a charge transfer state with low oscillator strength resulting in aggregation-caused quenching (ACQ) with low lifetimes and quantum yields. In the MOF pores, single flavin molecules represent a major population and the computed MIA@MOF structures do not find π-stacking interactions with the pore walls but only weak van-der-Waals contacts which reasons the enhanced fluorescence lifetime and quantum yield of the flavins in the composites compared to their neat solid state. To analyze the orientation of flavins in MOFs, we measured fluorescence anisotropy images of single flavin@MOF-5 crystals and a static ensemble flavin@MIL53 microcrystals, respectively. Based on image information, anisotropy distributions and overall curve of the time-resolved anisotropy curves combined with theoretical calculations, we can prove that all fluorescent flavins species have a defined and rather homogeneous orientation in the MOF framework. In MIL-53, the transition dipole moments of flavins are orientated along the 1D channel axis, whereas in MOF-5 we resolved an average orientation that is tilted with respect to the cubic crystal lattice. Notably, the more hydrophobic 6F-MIA exhibits a higher degree order than MIA. The flexible MOF MIL-53(Al) was optimized essentially to the experimental large-pore form in the guest-free state with QuantumEspresso (QE) and with MIA molecules in the pores the structure contracted to close to the experimental narrow-pore form which was also confirmed by PXRD. In summary, the incorporation of flavins in MOFs yields solid-state materials with enhanced rigidity, stabilized conformation, defined orientation and reduced aggregations of the flavins, leading to increased fluorescence lifetime and quantum yield as controllable photo-luminescent and photo-physical properties.

## 1. Introduction

**Figure 1:**
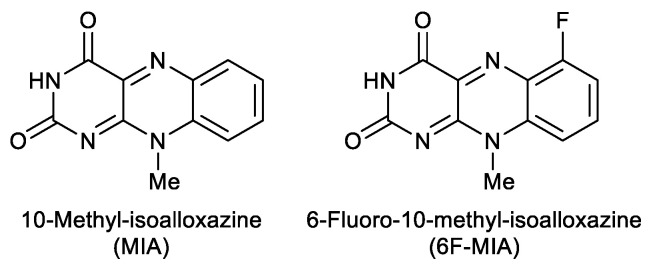
10-Methyl-isoalloxazine (MIA) and the mono-fluorinated derivative 6-fluoro-10-methyl-isoalloxazine (6F-MIA) [17].

Flavins represent a ubiquitous group of compounds in nature [1,2,3,4]. The basic structure consists of an isoalloxazine ring system (Figure 1) that has strong fluorescent properties [4,5]. Besides the well-known vitamin B2, flavins or riboflavins occur predominantly in combination with proteins as the so-called flavoproteins [1,6,7]. Flavoproteins play an important role in the respiratory chain of cells [1]. In addition, light-dependent biological processes such as flowering and circadian rhythm often employ this class of chromophore [8,9]. Flavins that are not bound to proteins are of great interest due to their photochemical and photophysical properties [10]. As in nature, flavins are used as photo-redox catalysts due to their redox properties [11,12]. In aqueous solution, all flavins exhibit strong absorptions in the ultraviolet and visible range. The absorption spectra typically show four bands with high molar absorptivities (>10^4^ L^−1^ mol cm^−1^) which are attributed to *π*→π* excitations [12,13,14]. The exact position of the absorption maxima, the absorptivity and the photoluminescence quantum yield depend significantly on the chemical structure of the flavin derivative and the chemical-physical environment, including solvatochromic effects [11,12,15,16].

In their neat solid state, the photoluminescence of dye molecules is often quenched by nonradiative processes. The incorporation of luminescent dye molecules into metal-organic frameworks (MOFs) as a dye@MOF composite tries to exploit the photophysical properties of single molecules in a well-defined solid-state environment for applications such as white-light emission, upconversion, non-linear optics, biological fluorescent imaging, chemical sensing, optical thermometry and others [18,19,20,21,22,23]. The structure and pore environment of MOF compounds can be designed by the selection of organic linkers and the metal cluster secondary building units (SBUs) [24,25,26,27,28,29,30,31]. Through incorporation into MOFs, the dye molecules could be protected from dynamic quenching, e.g., by O_2_ or solvent interactions. Aggregation-caused quenching (ACQ) of the dye in the solid-state is also effectively prevented by the confinement of separated single molecules in the pore system [18,32]. This protection could ensure consistent luminescence even under harsh environmental conditions. Conversely, targeted quenching of dyes by the selective additional adsorption of analyte species in dye@MOF composites could be interesting in sensor technologies [18,33,34,35,36,37,38,39,40].

A prerequisite for the MOF as a host for a dye guest with unchanged fluorescence is a minimal interference of MOF and dye. If the MOF should just act as a host matrix then ideally the MOF should show no fluorescence and color. Accordingly, aluminum-based MIL-53, [Al(OH)(bdc)] (bdc = benzene-1,4-dicarboxylate) [41,42,43,44,45,46,47,48,49,50] and zinc-based MOF-5, [Zn_4_O(bdc)_3_] [51,52] are promising host networks (see Section S3, Supplementary materials for structure details).

**Figure 2:**
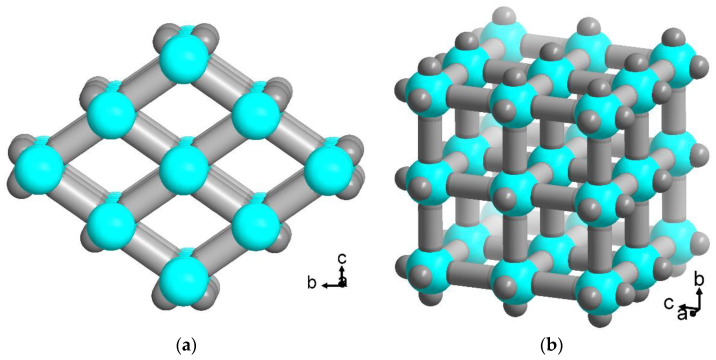
Schematic presentation of the periodic channel structures in (**a**) MIL-53, [Al(OH)(bdc)] and (**b**) MOF-5, [Zn_4_O(bdc)_3_] (bdc = benzene-1,4-dicarboxylate) (The inorganic building units {AlO_6_} and {Zn_4_(O)(O_2_)_6_}, respectively, are represented in cyan and the bdc linker schematically as a grey rod; see Section S3, Supplementary materials for structure details). (Structure images were drawn with Diamond [57] from the deposited cif files under CCDC-no./Refcode 220476/SABVUN [41] and 256966/SAHYOQ [52,58]).

The crystalline MOF host structures are often seen to give also a highly-ordered packing of the chromophore guests. Such an optimal alignment in host-guest MOF hybrid materials would be needed for polarized nonlinear optical responses [20,53] or to tune exciton coupling [54]. Yet, a correlation between photoresponse and anisotropy of dye guest molecules in ordered host matrices appears to be seldom done, e.g., in clays [55,56]. Here we chose the prototypical and well-characterized MOFs MIL-53(Al) with its periodic one-dimensional channels and MOF-5 with its three-dimensional orthogonal channel structure (Figure 2) for a proof-of-principle analysis of the anisotropy of encapsulated flavin dye molecules in these MOFs.

Furthermore, single crystals with an average diameter of 100 μm can be obtained for MOF-5 which allow the imaging of the embedded flavin with confocal laser scanning microscopy (CLSM) [59].

To the best of our knowledge we are not aware of flavin@MOF composites. Here, we report the incorporation of the flavin derivatives 10-methyl-isoalloxazine (MIA) and 6-fluoro-10-methyl-isoalloxazine (6F-MIA) (Figure 1) in the prototypical MOFs MIL-53 and MOF-5 and analyze the effects of the flavin@MOF composites on emission wavelength, the fluorescence lifetimes and photoluminescence quantum yields in comparison to the neat solid state and solution. The electronic structures of crystalline MIA, MIA@MIL-53 and MIA@MOF-5 and the resulting luminescent properties were calculated with plane-wave and QM/MM methods and compared with the experimental data.

## 2. Results and Discussion

The incorporation of the flavin derivatives 10-methyl-isoalloxazine (MIA) and 6-fluoro-10-methyl-isoalloxazine (6F-MIA) into MIL-53 and MOF-5 was carried out using a post-synthetic, diffusion-based incorporation into microcrystalline MIL-53 powders and an in situ embedding during MOF-5 synthesis.

### 2.1. Flavin@MIL-53 Synthesis

For the post-synthetic wet infiltration of MIL-53, different quantities of a saturated MIA or 6F-MIA solution in chloroform were added to the activated MOF MIL-53-lp and the suspension stored on an orbital shaker until all the solvent had been evaporated. The flavin@MIL-53 composites were washed with chloroform and then dried at 60 °C under dynamic vacuum. The MIA@MIL-53 composites contained 0.4 to 2.7 wt% of the flavin in the MOF and the 6F-MIA@MIL-53 composites between 0.1 to 1.5 wt% (see Supplementary Materials for details).

To further verify the incorporation of the flavins in the MOFs, nitrogen adsorption isotherms were recorded at 77 K (Figure S16). For very small amounts of incorporated flavin in MIL-53 (<1 wt% for MIA and 0.1 wt% for 6F-MIA), a slight increase in the internal surface area may occur (Table 1), which can be attributed to an additional washing effect exerted by shaking in the flavin solution. The proportional decrease in BET surface area and pore volume (Table 1) with an increase in flavin amount indicates successful incorporation into the network. The initial S_BET_ value of 1130–1150 m^2^/g for MIL-53 decreases to 80 m^2^/g (MIA@MIL-53) and 40 m^2^/g (6F-MIA@MIL-53), corresponding to a nearly nonporous structure which is due to pore blocking by occupation of the pore mouths with the flavin molecules. For the small wt% of the flavin the pore volume of the large-pore form of MIL-53 cannot be completely filled. Based on the crystallographic density ρ in the crystal structure of MIA of ρ = 1.6 g/cm^3^ [60] and 1/ρ = 0.63 cm^3^/g, the maximum flavin loading of ~4 wt%, that is 0.04 g(flavin)/g(composite), would only occupy a volume of 0.025 cm^3^/g or about 6% of the determined total pore volume of ~0.5 cm^3^/g in neat MIL-53.

When we consider the MIL-53 formula unit of [Al(OH)bdc] (M = 208.11 g/mol), then there is one channel segment of 13 Å (which is about the length of a MIA molecule) for 8 formula units. At a loading of 4 wt% MIA@MIL-53 we have 0.04 g MIA (0.175 mmol) in 0.96 g MIL-53 (4.6 mmol). Thus, the molar ratio of MIA molecules to the channel segments of 13 Å is 0.175:(4.6/8) = 0.3 or ~3.3 channel segments per MIA molecule. In other words, on average at this loading the MIA molecules can be ~2 × 13 Å = 26 Å apart, that is separated by 26 Å. At a loading of 1.5 wt% 6F-MIA@MIL-53, we have 0.015 g MIA (0.06 mmol) in 0.985 g MIL-53 (4.7 mmol). Thus, the molar ratio of 6F-MIA molecules to the channel segments of 13 Å is 0.06:(4.7/8) = 0.1 or ~10 channel segments per 6F-MIA molecule. On average at this loading the 6F-MIA molecules can be ~9 × 13 Å = 117 Å separated along a channel. Other diffusion-based incorporations into MOFs have shown that in such a post-synthetic modification the molecules remain trapped close to the pore mouths [59]. For MIL-53 with its flexibility and breathing mode, formation of the narrow-pore form can also occur upon activation, which precedes the nitrogen sorption measurement.

The phase analysis of the flexible MIL-53 network structure after flavin insertion and before activation by powder X-ray diffraction (PXRD) shows a superposition of the three MIL-53 phases from the comparison of the MIA@MIL-53 composites with the simulated MIL-53-as, MIL-53-np and MIL-53-lp diffractograms (Figure S17a). The samples with the two highest loadings (2.7 wt% and 3.9 wt% for MIA, 1.0 wt% and 1.5 wt% for 6F-MIA) were measured before and after activation (Figure S17b). The incorporated flavin affects the phase which the flexible MIL-53 network assumes. A loading of up to 2.7 wt% MIA gives only the narrow-pore form after activation which is understandable if the flavin molecules would occupy little of the inner pore volume and do not penetrate deep into the channels. At a loading of 3.9 wt% MIA, a superposition of the large- and narrow-pore form remains (Figure S17b). The comparison of the 6F-MIA@MIL-53 composites with the simulated MIL-53 PXRDs patterns show good matches with the MIL-53-lp form, independently of incorporated flavin amounts (Figure S18a). For 1.0 wt% 6F-MIA@MIL-53 after activation the PXRD is also a superposition of the large-and narrow-pore phases (Figure S18b). At the maximum loading of 1.5 wt% of 6F-MIA, almost no change in the diffractogram is evident upon activation, indicating retention of the large-pore form with just a trace formation of the narrow-pore phase (Figure S18b).

### 2.2. Flavin@MOF-5 Synthesis

The flavins were incorporated in situ into MOF-5, that is during the MOF formation, in order to achieve a uniform distribution and pore filling of the flavins throughout the network [51,59]. From a comparison of the dimensions of MIA and 6F-MIA (4 × 8.5 × 12 Å) (Figure S12) with the pore diameter of 15 Å in MOF-5, which is larger than its pore window size (8 × 8 Å) (Figure S15), the flavin molecules can be very well incorporated during the crystal formation. By using concentrated solutions of the flavins in dimethylformamide (DMF), cubic single crystals with an edge length up to 1 mm were synthesized according to a protocol by Han et al. [62].

The incorporated flavin amounts were determined to 0.85 wt% for MIA@MOF-5 by UV/Vis spectroscopy from the supernatant solution (Section S1.2) and to 5.2 wt% for 6F-MIA@MOF-5 by postsynthetic digestion ^1^H NMR (Section S1.2). The higher amount of 6F-MIA is explained by the very good solubility of 6F-MIA in DMF for a saturated solution of *c*_6F-MIA_ = 2640 mg∙L^−1^ whereas MIA was much less soluble, giving only *c*_MIA_ = 272 mg∙L^−1^ as a saturated solution (Section S2). From the crystallographic density of MIA of ρ = 1.6 g/cm^3^ [60] and 1/ρ = 0.63 cm^3^/g, the 6F-MIA loading of 0.052 g(6F-MIA)/g(composite) would still occupy only a volume of 0.03 cm^3^/g or about 3% of the total pore volume of ~1.0 cm^3^/g in neat MOF-5 [52]. The MOF-5 formula unit is [Zn_4_O(bdc)_3_] (M = 769.87 g/mol). There is 1 “pore” per formula unit. At a loading of 0.85 wt% MIA@MOF-5 we have 0.0085 g MIA (37 µmol) in 0.9915 g MOF-5 (1.3 mmol). Thus, the molar ratio of MIA molecules to pores is 0.037:1.3 or 0.029 (MIA per pore), or ~34 pores for each MIA molecule. At a loading of 5.2 wt% 6F-MIA@MOF-5 we have 0.052 g MIA (0.21 mmol) in 0.95 g MOF-5 (1.2 mmol). Thus, the molar ratio of 6F-MIA molecules to pores is 0.21:1.2 or 0.18 (6F-MIA per pore), that is on average 5.7 pores for each MIA molecule.

Powder X-ray diffraction shows a good match between the flavin@MOF-5 composites, synthesized MOF-5 crystals and the simulated MOF-5 diffractogram (Figure S19).

**Figure 3:**
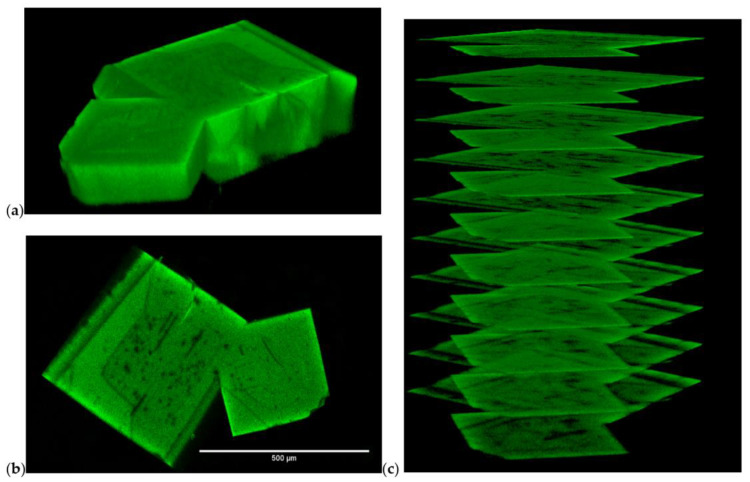
Confocal images of MIA@MOF-5. (**a**) Confocal laser scanning microscopy 3D profile, (**b**) sectional plane at 200 µm from top, and (**c**) stack of sectional planes of MIA@MOF-5 (cw excitation at λ_ex_ = 458 nm, λ_em_ = 530–555 nm). Every sectional plane corresponds to a measurement thickness of 4 μm, which corresponds to the optical resolution of the used objective UPLSAPO10X/0.4NA.

**Figure 4:**
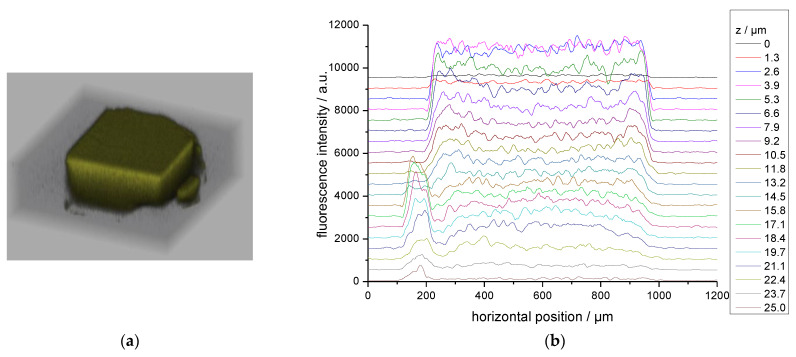
(**a**) Confocal laser scanning microscopy 3D profile for a height *z* of 25 µm, (**b**) line profile along *z* of 6F-MIA@MOF-5 (cw excitation at λ_ex_ = 458 nm, λ_em_ = 530–555 nm, objective UPLSAPO10X/0.4NA). The fluorescence intensity decreases with the depth due to the inner filter effect.

The large flavin@MOF-5 crystals were analyzed by confocal laser scanning microscopy (CLSM) for the distribution of MIA (Figure 3) and 6F-MIA (Figure 4) in the MOF. The fluorescence images of the sectional planes support an even incorporation of the flavin dyes throughout the whole MOF crystals during the in situ synthesis.

### 2.3. Fluorescence Spectroscopy and Photophysics

Before discussing the fluorescence properties of the flavin@MOF composites in detail, it is important to consider which fluorescent species can be expected upon incorporation in MOFs. Since we varied the loading rate of incorporated flavins, we computed the probability *p* that a certain occupation number of flavins per site could be incorporated in MOFs assuming a random distribution of dyes. Using the average numbers of flavins per site *n_av_(flavins*/*site)* approximated in Section 2.1 and Section 2.2, our computation shows that the probability *p(> one)* that more than one flavin is incorporated per site can be significant for our conditions (Table 2). The probability *p(> one)* is higher in MIL-53 already at low loading as compared to MOF-5. In this context, it important to take into account that organic chromophores with an extended π system can stack in solution with increasing concentration so that H- or J-aggregates of variable composition and structural arrangements can be formed. Due to the affinity of these chromophores to associate, the computed probabilities *p(> one)* represent only lower limits that will be even higher in reality. Thus, flavin confinement in MOF composites could lead to J- or H-aggregates of variable stoichiometry. Notably, the emission properties of the chromophore aggregates critically depend on the strength of the excitonic coupling and on charge transfer interactions which can be influenced by their orientation stoichiometry and chemical structure. Therefore, we can expect variable fluorescent species for flavin@MOFs. Accordingly, we investigated in the following, whether the measured fluorescence spectra, lifetimes and anisotropies indicate the presence of multiple fluorescent species that could be related to aggregates.

**Figure 5:**
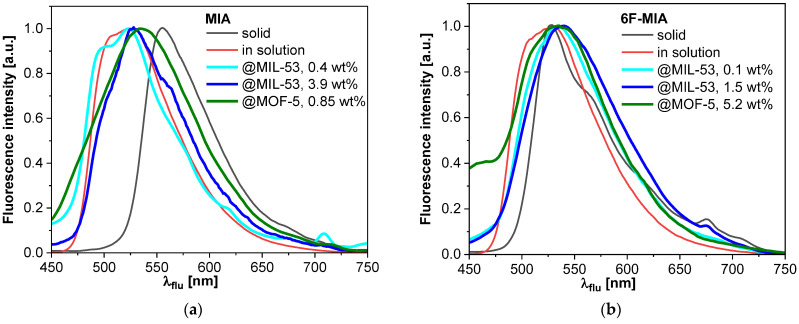
Normalized fluorescence spectra at room temperature of (**a**) MIA and (**b**) 6F-MIA in their neat solid state, in 1:1 DCM/MeOH solution and for the flavin@MOF composites; wt% refers to the flavin loading. Since the fluorescence signal of flavin@MOF composites with low loading is weak, the contributions of scattered excitation light at short emission wavelengths becomes relevant, which causes additional shoulders in the spectra at short wavelengths.

The fluorescence spectra at room temperature of both flavins, in a solution of 1:1 DCM/MeOH and in solid amorphous powder, are compared with their composites (bold lines) in Figure 5 for measurements at room temperature. Additional fluorescence spectra at 77 K were measured for all compounds and are compared with the corresponding spectra at room temperature in the supplementary materials Section S5, Figures S20–S23. The fluorescence spectrum of solid MIA is strongly red shifted with respect to the solution in 1:1 DCM/MeOH, while this shift is much weaker for 6F-MIA where a shoulder appears around 650 nm. The fluorescence spectra of flavin@MOF composites reveal a broadening at the bathochromic side that is stronger for higher loadings. We conclude that a mixture of emitters (flavin monomers and aggregates) exists. Due to the bathochromic shift of the spectra and the presence of shoulders for lower emission energies, we identify fluorescent J-aggregates as additional emitters. Consistently, the presence of bathochromically shifted emitters was enhanced for high flavin loadings at 77 K.

Next, we analyzed the fluorescence lifetimes and quantum yields. Table 3 summarizes fluorescence lifetimes (*τ*), quantum yields (*Φ*_F_*)* and the wavelengths of the fluorescence maxima (*λ_F,max_*) of the flavin dyes MIA, 6F-MIA and the flavin@MOF composites, which were measured with a time-resolved fluorescence spectrometer front-face sample holder (fluorescence spectra in Figure 5 and Figures S20–S23) and multiparameter fluorescence image spectroscopy (MFIS) with polarization resolved detection, both using single photon timing electronics. All measured time-resolved fluorescence decays of both instruments are displayed in Figures S23–S33. The time-resolved fluorescence measurements in the fluorescence spectrometer and the confocal microscope with multiparameter fluorescence detection (see Section 3) gave consistent results. All fluorescence decays of flavin@MOF composites are multi-exponential. In addition to three fluorescence lifetimes in the ns range, polarisation-resolved MFIS detected a significant fraction of depolarized signal (i.e., fluorescence and not scattered excitation light) with a lifetime of 30 ps, which indicates the presence of strongly quenched fluorescent species. This agrees with the drop of the fluorescence quantum yields, *Φ*_F_, observed for flavin@MOF composites. Notably, the fluorescence lifetimes of solid flavins are all in low picosecond time ranges, too. This finding agrees also with the quantum-chemical calculations presented in Section 2.4. below and in supplementary material section S6. Although the fluorescence quantum yields, *Φ*_F_, in flavin@MOF composites indicate partially quenched fluorescence species, the *Φ*_F_ values are up to 8 times higher than for solid flavins. Moreover, in agreement with Table 2, we observe a qualitative correlation between lower fractions of quenched species for low loadings and *n_av_(flavins/site)*. The flavin’s tendency of forming J-aggregates is already visible in solution measurements of pure dyes. While at least three fluorescent species (most likely a small fraction of fluorescent aggregates) are detectable by up to three lifetimes in the solvent 1:1 DCM/MeOH dissolving flavins only moderately, a single fluorescent species is detectable in water, where flavins are dissolved well. In 1:1 DCM/MeOH, the longest lifetime with species fraction between 10 and 20% is in the range of 8 to 10 ns that is longer than the lifetime of the dye in water. The solid-state measurements and quantum-chemical calculation suggest that weakly and strongly fluorescent aggregates can coexist.

We applied confocal multi-parameter fluorescence image spectroscopy (MFIS) to map also the spatial and orientation dependence of the fluorescence properties for the microcrystalline sample of flavin@MIL-53 and large single crystals of flavin@MOF-5.

**Figure 6:**
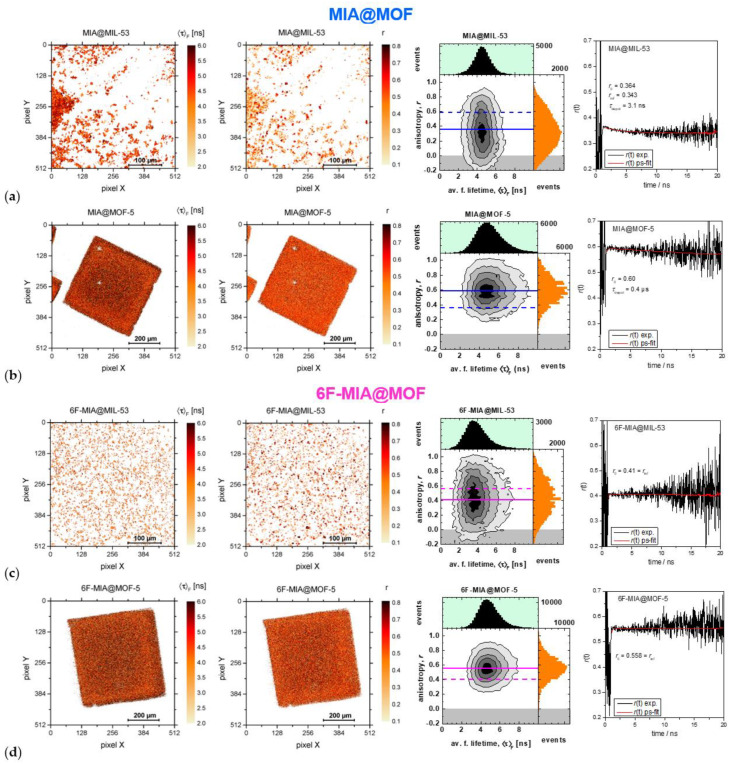
Images of MIA and 6F-MIA in two MOFs obtained by confocal multi-parameter fluorescence image spectroscopy (MFIS) (see Section 3). (**Panel 1**) Image of fluorescence-weighted average lifetime; (**Panel 2**) Image of experimental steady state fluorescence anisotropy *r*; (**Panel 3**) Interrelation ${\langle \tau \rangle }_{F}-r$ in the 2D diagram of the parameter images in panels 1 and 2 with a full horizontal line for the average anisotropy $\overset{-}{r}$ of the sample and second dashed line of other MOF composite as reference; and (**Panel 4**) Time-resolved fluorescence anisotropy curves *r(t)* to resolve the fundamental anisotropy r_0_ and the depolarization time τ_depol_ that are displayed in the individual panels 4. (**a**) MIA@MIL-53 (3.9 wt%), (**b**) MIA@MOF-5, (**c**) 6F-MIA@MIL-53 (1.5 wt%) and (**d**) 6F-MIA@MOF-5. For flavin@MOF-5 five slices in the center of the z-stack were selected to generate the decay histograms, for flavin@MIL53 all photons from the images were used. The acquisition conditions were λ_ex_ = 440 nm (pulsed@32MHz, objective UPLSAPO10X/0.4NA for MIL-53 and objective UPLSAPO20X/0.75NA for MOF-5), λ_F_ = 502–538 nm, under air.

The average fluorescence lifetime ${\langle \tau \rangle }_{F}$ of MIA@MIL-53 (3.9 wt%) (Figure 6a, panel 1) and a crystal of MIA@MOF-5 (Figure 6b panel 1) yielded 4. 5 and 4.7 ns, respectively. For 6F-MIA@MIL-53 (1.5 wt%) and 6F-MIA@MOF-5 the values of ${\langle \tau \rangle }_{F}$ are 3.5 and 4.5 ns (Figure 6c,d panel 1), respectively. These results are consistent with the lifetimes measured in the fluorescence spectrometer. The average fluorescence lifetime values of the chromophores in flavin@MOF composites are similar to the values in aqueous solution (Table 3). Interestingly, the fluorescence lifetime of MIA is affected by the location in the crystal. The lifetime of MIA is slightly but significantly longer in the center of the MOF-5 crystal (Figure 6b—the false colors for lifetime values are darker in the middle (5.5 ns)). This positional effect explains the surprising result that the fluorescence lifetime distribution of MIA is broader in MOF-5 than in MIL-53 crystals (Figure 6b vs. a). Being aware that deactivation processes of MIA and 6F-MIA in solution include fluorescence, intersystem crossing and internal conversion with different relative efficiencies [17], we note that both methods for lifetime analysis indicate that MIA is the less sensitive derivative as compared to 6F-MIA.

With higher loading of the flavins into MIL-53, *λ_F,max_* shifts to slightly longer wavelengths (Table 3). A similar shift is seen for MIA when going from an aqueous to a DCM/MeOH solution and oppositely for 6F-MIA between a DCM/MeOH and water solution. In general, conditions where the flavin molecules come closer together enable charge transfer processes and self-quenching between them, resulting in spectral shifts and broadening of the emission bands with loss of fine structure and reduction of the fluorescence quantum yield. It is known that the photophysical properties of a chromophore in a MOF are influenced by the geometric restrictions imposed by the framework structure [18]. In all flavin@MOF composites, a strong increase in both lifetimes and quantum yields compared to the neat solid flavin is observed. In the case of MIA@MIL-53 and MIA@MOF-5 a ~10-fold increase in lifetime and a 3 to 5-fold increase in quantum yield can be observed compared to solid MIA. For the 6F-MIA@MOF composites, these increases are even more evident, with a ~50-fold increase in lifetime and ~3-fold increase in quantum yield. Compared to the neat solid flavins the incorporation of the molecules within the MOFs reduces the nonradiative decay rates, leading to increased fluorescence intensities, lifetimes, and quantum yields. This effect is especially prominent in MFIS. The photophysical parameters of the flavin@MOF composites correspond to the data of the flavins in solution (Table 3) fostering the concept of “solid solutions” for dye@MOF composites.

From the MFIS analysis, it is evident that microcrystals of flavin@MIL-53 vary significantly in both size and polarization properties (Figure 6a,c panel 2). The orientational distributions of both flavin chromophores is indicated by a broad distribution of the fluorescence anisotropies *r* due to varying size and orientation of the flavin@MIL-53 micro-crystallites (Figure 6a,c). The anisotropies are calculated according to Schaffer et al. [63]. For this discussion of the *r*-distributions, it is important to consider the broadening of the distributions due to shot noise caused by the relatively small average number of photons per pixel ($\bar{N_{p}}$(MIA) = 35) and $\bar{N_{p}}$(6F-MIA) = 200). 6F-MIA@MIL-53 exhibits the broadest *r*-distribution reaching its upper limit (*r* = 1) and lower limit (*r* = 0). To reduce shot noise, we integrated the signal of the whole image to compute time-resolved anisotropy curves *r(t)* (Figure 6a,c panel 4). This was accomplished by global fits with iterative reconvolution of the instrumental function (grey curves in Figure S34) to p- and s-polarized decays as described in ref. [63,64]. The fits required 4 fluorescence lifetime components (lifetimes and species fractions are compiled in Table 3) and one (two for MIA@MIL-53) depolarization correlation time.

It is especially remarkable, that *r(t)* of 6F-MIA@MIL-53 starts at a fundamental anisotropy *r*_0_ = 0.4 and exhibits no decay. This is direct proof the MFIS images resolve the distribution of particles whose orientation on the surface is static and random so that the isotropic average of r is obtained, which is usually mathematically derived in text books [65] considering the case of fluorophores in solution. Three arguments (the anisotropy of pixels of some MIA and 6F-MIA@MIL-53 microcrystals reaches one (see Figure 6a,c, panels 2 and 3 and Figure S35), geometric constraints of available space in MIL-53 and quantum-chemical calculations (see below)) allow us to conclude that transition dipole moments for absorption and fluorescence (TDM) (see supplementary materials Section S6.5 Figure S42) of the various fluorescent MIA and 6F-MIA species must be oriented along the one-dimensional channel of MIL-53 with no large deviation among each other. Considering MIA@MIL-53, the fundamental anisotropy *r*_0_ = 0.364 is slightly smaller and an additional decay term with a depolarization time of 3.1 ns is observed. From this it is evident that the order of the MIA TDMs is slightly lower, but nevertheless quite similar for all fluorescent species. The TDMs are preferentially orientated along the channel axis.

In contrast to MIL-53, MOF-5 provides a very well defined and ordered porous framework with large flavin@MOF-5 crystals (Figure 3, Figure 4 and Figure 6b,d) as indicated by a narrow distribution of fluorescence anisotropies *r*, which are centered around mean anisotropy values: $\bar{r}$(MIA) = 0.6 (full blue line in Figure 6b, panel 3) and $\bar{r}$(6F-MIA) = 0.56 (full magenta line in Figure 6d, panel 3). Both flavin@MOF-5 samples show high anisotropies that indicate distinct oriented binding sites, excluding random orientation of the dye molecules in the crystal. As for 6F-MIA@MIL53, no time-dependence of the anisotropy *r(t)* was observed for 6F-MIA@MOF-5. MIA embedded in either of the two MOFs shows limited depolarization on the ns to µs scale, which could have one or several of the following reasons: (i) constrained rotational motion, (ii) resonance energy transfer to chromophores in close vicinity and/or (iii) slightly differing orientations of fluorescent species with distinct lifetimes.

Due to the high symmetry of the cubic MOF-5 crystal for the x, y, z directions defined in the crystal parameters, it is feasible and appropriate to assume an identical rotation of the transition dipole moment of the MIA chromophore using the normalized rotation vector of the crystal ***n*_CA_** in an angle axis representation that rotates the S_0_-S_1_ absorption transition dipole moment *A* of the chromophore with respect to the crystal axis *c* by an angle ω_CA_. For a cube, the directions of the four potential rotation vectors correspond to the respective body diagonals. Since the body diagonals are equivalent, we consider in the following only one rotation vector. Using the experimental observables for 6F-MIA@MOF-5 ($\bar{r}$(6F-MIA) = 0.56 (Figure 6d panel 3) and α = 8.2° (Figure 6d panel 2)) and for MIA@MOF-5 ($\bar{r}$(MIA) = 0.6 (Figure 6b panel 3) and α = 65° (Figure 6b panel 2)), a unique solution with an angle of the normalized rotation vector ω_CA_ = 13° (for MIA) and ω_CA_ = 16° (for 6F-MIA) is obtained that describes the average orientation of the TDM for absorption of all fluorescent species defined by the Euler angles relative to the crystal lattice *θ*_CA_~12 ± 1 and *θ*_CA_~40.5 ± 1, i. e., the TDM is not oriented along one of the crystal axes. This confirms that MOF-5 provides a suitable frame to incorporate flavins in a defined average orientation regardless of their aggregation.

### 2.4. QM/MM Excited State Computations

**Figure 7:**
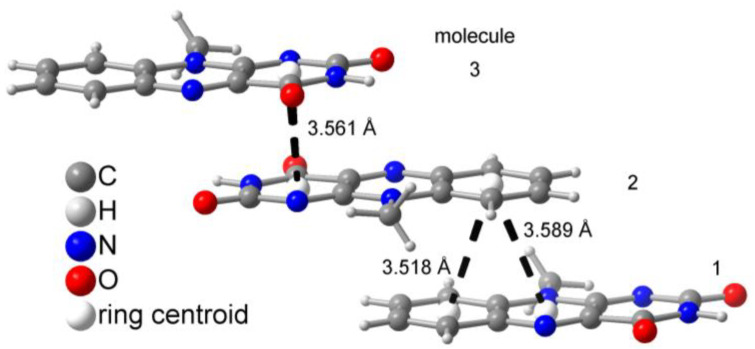
π−π stacking modes in crystalline MIA with centroid-centroid distances (graphics were drawn from the deposited cif file with CCDC Refcode MISALX [60]). The numbers 1, 2 and 3 differentiate the molecules for their different stacking interactions (see text).

In the neat MIA crystal [60], we can identify two orientations of stacked neighboring molecules (Figure 7). In one orientation, π-stacking interactions occur between the terminal phenyl and the central and terminal ring of two adjacent MIA molecules (molecule no. 1 and 2 in Figure 7) with a centroid-centroid separation of 3.518 and 3.589 Å. The other π-stacking orientation involves only the terminal phenyl rings of two adjacent MIA molecules (no. 2 and 3) at a centroid-centroid distance of 3.561 Å.

Due to the different π-stacking modes in the crystal of neat MIA, QM/MM geometry optimizations of ground and excited state structures had to include at least three MIA molecules for a sensible description of interactions with all direct neighbors (see Section S6.1). Compared to the single MIA chromophore in vacuum (λ_abs, max_ = 401 nm), the calculated maximum absorption value in the crystal is 441 nm, thus significantly red-shifted. The calculated emission from S_1_ state in the crystal occurs at ca. 540 nm, compared to ca. 555 nm in the solid-state experiment and 505 nm for MIA calculated in vacuum (Table 3) with a calculated small oscillator strength (f = 0.025) and an emission rate constant of 5.87·10^6^ s^−1^. While for the single MIA molecule S_1_ corresponds to a locally π-excited state, orbital analysis for the three-molecule MIA complex reveals a dominating charge-transfer character. Here, electron density is relocated between the two molecules that overlap with two aromatic rings (number 1 and 2 in Figure 7). This is the main reason for the loss in fluorescence emission intensity in the crystalline state.

Optimization of MIL-53-lp with QuantumEspresso (QE) revealed essentially the same results as the experimental structures (Table S3). Compared to the reported orthorhombic large-pore form MIL-53-lp (CCDC 220476 [41]) the computed guest-free MIL-53-lp structure after optimization has nearly the same cell axes lengths within 0.7 Å (Figure S40, Table S3). With one MIA molecule per unit cell, the structure becomes more similar to the reported narrow-pore form (Figure S41, Table S3). A variation of lattice parameters upon adsorption in MIL-53 was also seen, e.g., upon loading with xylene isomers [66], short linear alkanes [67] or methane [68].

**Figure 8:**
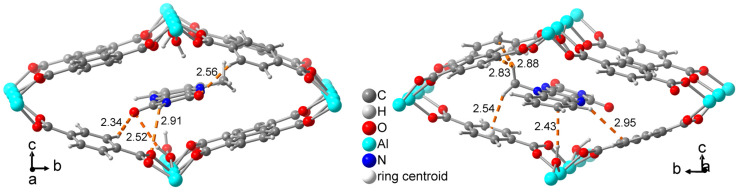
Computed structure of MIA@MIL-53 viewed from both ends of the MIA molecule with indication of the weak C-H···O, C-H···π, C-H···C, N-H···O, O-H···O and O-H···C interactions (H···X distances in Å, distances above 3 Å are not shown).

The position of the MIA molecule is optimized with its length along the channel direction and close to the center of the channel (Figure 8). There are no evident short and strong supramolecular interactions but the MIA molecule is held in place by weak C-H···O, C-H···p, C-H···C, N-H···O, O-H···O and O-H···C interactions. Even the N-H···O and O-H···O contacts have rather long H···X distances above 2.5 Å (Figure 8).

**Figure 9:**
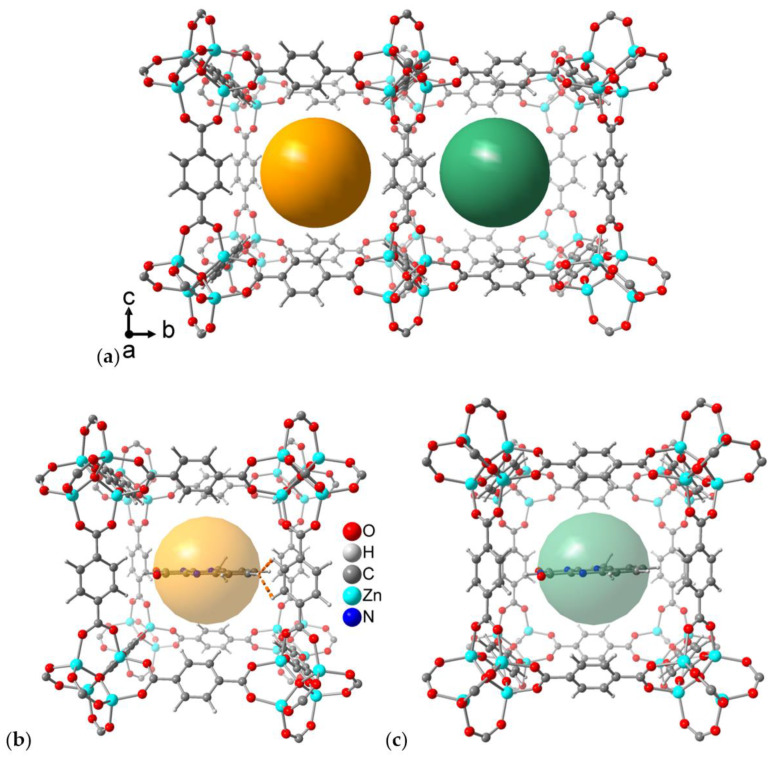
(**a**) Alternating neighboring pores in MOF-5 with different cages for binding MIA. Orange pore at left: Linker phenyl hydrogens face *inwards* toward the center of the pore. Green pore at right: outward position of linker phenyls. (**b**) Computed MIA molecule in the *inward*-cage in MOF-5 with the only two supramolecular contacts below 3.0 Å (two C-H···H-C contacts) indicated as dashed orange lines. (**c**) Computed MIA molecule in the *outward*-cage for which there are no supramolecular contacts below 3.0 Å.

For neat MOF-5, we note a slight compression of the unit cell constants upon optimization by ca. 0.2 Å in each direction, compared to the experimental values [51,58]. The cell size does not significantly change when MIA is inserted. Due to the tilted arrangement of the phenyl rings, MOF-5 offers two different types of cages to host the MIA chromophore (Figure 9a). In one cage, the hydrogen atoms of the phenyl linkers point inwards toward the center of the pore (left pore with orange sphere in Figure 9a,b). We refer to this possibility as *inward-cage*. In the second alternative, the hydrogen atoms of the phenyls face *outwards*, thus offering a wider pore (right pore with green sphere in Figure 9a,c and pore in Figure S15d). The energy for MIA incorporated in the *inward*-cage is ca. 15.3 kJ/mol lower than in the *outward*-cage because of increased attractive van-der-Waals interactions. The computed binding energy of MIA is ca. 49 kJ/mol in the *inward*-complex and ca. 35 kJ/mol in the *outward*-configuration, suggesting that complexation in the *inward*-cage is preferred. There is, however, no evident supramolecular interaction in either case. The MIA molecule is computed in the center of the pores, along the horizontal channel direction (Figure 9b,c). All distances from MIA to the framework walls are larger than 3.0 Å, except for two C-H···H-C contacts in the *inward*-cage position (Figure 9b) with an H···H distance of 2.5–2.6 Å. The next-nearest distances are four C-H···O contacts of ~3.2 Å, also in the *inward*-cage position.

**Figure 10:**
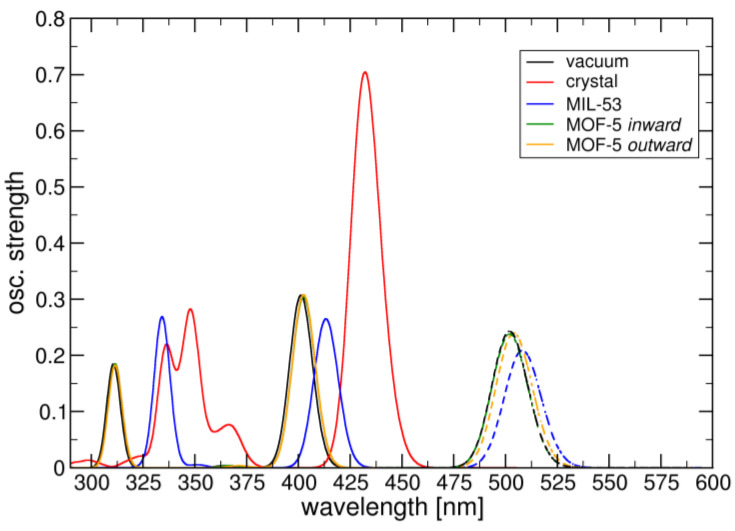
DFT/MRCI computed absorption (full lines) and emission (dashed lines) signatures of MIA in vacuum, neat crystal and MOF environments.

In the MIA@MIL-53 complex, the ground state QM/MM optimization converges to essentially the same structure as with plane-wave methods. The lowest excitation wavelength is 414 nm with an oscillator strength f of 0.265 and local π-excitation character, corresponding to HOMO-LUMO excitation. The computed emission wavelength from the S_1_ state is 509 nm (f = 0.209) (Figure 10) with an emission rate constant of 5.39·10^7^ s^−1^, about ten times larger than in the neat MIA crystal. The absorption and emission wavelength values are slightly blue-shifted with respect to the experimental measurement (523 to 527 nm, depending on the MIA wt%). The oscillator strength compares well with the value for MIA in vacuum (f = 0.243), demonstrating that the MOF environment efficiently increases fluorescence emission by preventing quenching due to π-stacking.

For MOF-5, we find no significant change in absorption and emission wavelength values depending on the mode of complexation. In the more stable *inward* form, maximum wavelength absorption occurs at 402 nm (f = 0.305), while emission from S_1_ is at 502 nm (f = 0.238) with a rate constant of 6.30·10^7^ s^−1^. Similar to MIL-53, the S_1_-excitation shows the same characteristics as in vacuum.

## 3. Materials and Methods

### 3.1. Chemicals and Equipment

Reagents were obtained from commercial sources (Table S1) and used without further purification. All flavin derivatives [17,69,70] and metal-organic frameworks [41,62,66,71] were synthesized and purified according to the literature.

Powder X-Ray diffractograms (PXRD) were measured on a Bruker D2 phaser bench-top diffractometer (Bruker, Ettlingen/Karlsruhe, Germany, 300 W, 30 kV, 10 mA) at ambient temperature using Cu-Kα radiation (λ = 1.5418 Å) at a scan rate of 0.0125°/s. The measurements were performed with a flat silicon, low-background sample holder. This Bragg–Brentano geometry broadens the beam spot at low angles so that only a fraction of the diffraction radiation reaches the detector with reflections measured at 2θ *<* 7° have lower than expected intensity.

Nitrogen physisorption isotherms were obtained at 77 K on a Nova 4200e S/N volumetric gas sorption analyzer from Quantachrome (Boynton Beach, FL, USA), equipped with degassing and three analyses stations. Before the gas sorption experiment, the sample was weighed into a glass tube capped with a septum. The tube was connected to the degassing port of the Nova instrument and degassed under dynamic vacuum of 1·10^−2^ mbar at a temperature of 120 °C for 3 h, then weighed again and transferred to an analysis port of the Nova device.

The Brunauer–Emmett–Teller (BET) surface areas were calculated from the nitrogen adsorption isotherms in the p/p_0_ range from 0.017 to 0.07. Total pore volumes were calculated from the nitrogen sorption isotherm at p/p_0_ = 0.95.

Fluorescence microscopy. Characterization of the incorporated fluorophores by confocal laser scanning and multi-parameter fluorescence image spectroscopy (CLSM and MFIS [72]) (condition g in Table 3) was carried out with a modified Olympus Fluoview 1000 inverse confocal microscope system (Olympus, Hamburg, Germany) with an internal 405 nm laser and a 6-channel single photon detector on dye@MIL-53 (20 × objective) and dye@MOF-5 (10 × objective). For the excitation of CLSM a cw argon laser with a wavelength of 458 nm was used. For MFIS, the acquisition conditions were λ_ex_ = 440 nm (pulsed@32MHz, objective UPLSAPO10X/0.4NA for MIL-53 and objective UPLSAPO20X/0.75NA for MOF-5), λ_em_ = 502–538 nm, under air. Experimental anisotropy decays in Figure 6 were calculated from the measured p- and s-polarized fluorescence decays (Figure S34) after subtracting the observed background according to: $r=(GF_{∥}-F_{⊥})/(\left(1-3l_{1}\right)GF_{∥}+(2-3l_{2})F_{⊥})$. The correction factors, taking into account polarization mixing in high-NA objectives were *l*_1_ = 0.0308 and *l*_2_ = 0.0368 [73]. The model curves (red lines in Figure 6) are constructed in the same way from the fits to the decays. Global fits by iterative reconvolution of the instrumental function (grey curves) to p- and s-polarized decays required 4 fluorescence lifetime components (Table 3 main document) and one (two for MIA@MIL-53) rotational correlation time. The offsets in the decays are caused by afterpulsing of the detectors and taken into account by the fitting routine. The G-factor, compensating differences in the detection efficiencies in the two polarization channels, of G = 0.97 was determined by fitting polarized fluorescence decays of an aqueous solution of Rhodamine 110. The doped MOFs were studied under air.

Steady-state excitation and emission spectra were recorded on a FluoTime 300 spectrometer from PicoQuant (Berlin, Germany) equipped with a 300 W ozone-free Xe lamp (250–900 nm), a 10 W Xe flash-lamp (250–900 nm, pulse width <10 µs) with repetition rates of 0.1–300 Hz, an excitation monochromator (Czerny-Turner 2.7 nm/mm dispersion, 1200 grooves/mm, blazed at 350 nm and 600 g/mm), diode lasers (pulse width <80 ps) operated by a computer-controlled laser driver PDL-820 (repetition rate up to 80 MHz, burst mode for slow and weak decays), two emission monochromators (Czerny-Turner, selectable gratings blazed at 500 nm with 2.7 nm/mm dispersion and 1200 grooves/mm, or blazed at 1250 nm with 5.4 nm/mm dispersion and 600 grooves/mm) with adjustable slit width between 0 mm and 10 mm, Glan-Thompson polarizers for excitation (Xe-lamps) and emission, a Peltier-thermostatized sample holder (−40–105 °C), and two detectors, namely a PMA Hybrid 40 (transit time spread FWHM <120 ps, 200–900 nm) and a R5509-42 NIR-photomultiplier tube (transit time spread FWHM 1.5 ns, 300–1400 nm) from Hamamatsu. The signal-to-noise ratio (optical noise) is typically better than 29000:1, as measured with double monochromators in the excitation and emission light path. Steady-state photoluminescence spectra and fluorescence lifetimes were recorded in TCSPC mode by a PicoHarp 300 (minimum base resolution 4 ps), whereas phosphorescence was measured in the MCS mode by a TimeHarp 300, where up to several ms can be detected. Emission spectra were corrected for source intensity (lamp and grating) by standard correction curves. For samples with lifetime in the ns order, an instrument response function calibration (IRF) was performed using a diluted Ludox^®^ solution. The lifetime analysis was performed using the commercial EasyTau 2.2 software. The quality of the fit was assessed by minimizing the reduced chi squared function (χ^2^) and visual inspection of the weighted residuals and their autocorrelation. Absolute photoluminescence quantum yields were measured with a Hamamatsu Photonics measurement system (C9920-02) equipped with a L9799-01 CW Xenon light source (150 W), a monochromator, a C7473 photonic multi-channel analyzer, an integrating sphere and employing the U6039-05 PLQY measurement software (Hamamatsu Photonics, Ltd., Shizuoka, Japan). All solvents used were of spectrometric grade (Uvasol^®^).

### 3.2. Flavin@MIL-53 Synthesis

Post synthetic wet infiltration was performed by adding different quantities (1 to 15 mL) of saturated solutions of the flavins in chloroform (*c*_MIA_ = 69.5 mg∙L^−1^, *c*_6F-MIA_ = 20.25 mg∙L^−1^) to activated microcrystalline MIL-53 powders (20 mg) in 25 mL glass vials. The different suspensions were stored on an orbital shaker until all the solvent had been evaporated. The flavin@MIL-53 composites were washed three times with chloroform for 5 min at each washing step and then dried at 60 °C under dynamic vacuum. Since the filtrate was already colorless after the first washing process, all of the dye from the initial solution was incorporated in the solid MIL-53 sample. The MIA@MIL-53 composites contained 0.4 to 2.7 wt% of the flavin in the MOF and the 6F-MIA@MIL-53 composites between 0.1 to 1.5 wt% (see also Section S1.2).

### 3.3. Flavin@MOF-5 Synthesis

In situ MOF-5 crystallization was performed by synthesizing the host network in saturated solutions of the flavins in dimethylformamide, DMF (*c*_MIA_ = 272 mg∙L^−1^, *c*_6F-MIA_ = 2640 mg∙L^−1^). The amount of incorporated flavin was then quantified using UV/Vis or postsynthetic digestions ^1^H NMR. The MIA@MOF-5 composites contained 0.85 wt% (UV/Vis) and 6F-MIA@MOF-5 composites contained 5.2 wt% (^1^H NMR) of the flavin (see also Section S1.2).

### 3.4. Computational Methods

Crystal structures of MIA (CCDC Refcode MISALX [60]), MIL-53(Al) (CCDC no./Refcode 220476/SABVUN for -MIL-53-lp [41]) and MOF-5 (CCDC-no./Refcode 256966/SAHYOQ [52,58]) were extracted from experimental data. The geometries were optimized with Quantum Espresso (QE) [74] using RRKJ-pseudopotentials [75], the generalized gradient approximation (GGA) with PBE-exchange correlation [76], Grimme-type D3-dispersion corrections [77] and a kinetic energy cutoff of 40 eV. SCF computations were performed including only the gamma point. For the optimization of the MIA crystal the cell parameters were frozen to the experimental values. MIL-53(Al) and MOF-5 were optimized without any constraints. To obtain the MOF-MIA complexes, one MIA molecule was placed into the center of the unit cell. To fit the MIA chromophore, the MIL53 the unit cell was replicated three times in a-direction, comprising a 3 × 1 × 1 supercell. The resulting geometries were then fully relaxed using the aforementioned methodologies. These structures were used as input structures for QM/MM cluster model computations.

Atomic partial charges for the MIA chromophore were computed with Gaussian16 [78] using RHF, the 6-31G* basis set and the Merz–Kollmann scheme [79]. Periodic charges for the frameworks were generated with the REPEAT method [80] implemented in CP2K [81]. Atoms herein were described with DZVP-MOLOPT-GTH basis sets and corresponding GTH-PBE pseudopotentials [82]. Force field parameters for MIA were obtained from the Generalized Amber Force Field (GAFF). MOF-5 force field parameters were adapted from a previous work by Vanduyfhuys et al. [83]. MIL-53(Al) parameters were computed with the FFTK program suite [84] using cluster models optimized with Gaussian16 (see Section S6.2 for details).

Finite cluster models were generated for each structure using the previously optimized geometries. The MOF structures were saturated accordingly (see Sections S6.2 and S6.3 for details). The MIA crystal cluster was prepared by replicating the unit cell eight times in each direction, thus including 1024 individual MIA molecules with a cell size of ca. 40 × 50 × 60 Å^3^. The size of the MIL-53 cluster was ca. 60 × 30 × 30 Å^3^, the MOF-5 cluster had ca. 98 × 98 × 98 Å^3^.

For QM/MM geometry optimizations, the COBRAMM2.0 package [85] was employed, choosing Gaussian16 [78] for the computation of the QM part (MIA) and AMBER16 [86] for the MM part (i.e., the surrounding). The QM-part was described with the PBE0 functional. The MIA crystal model was computed with different sizes for the central QM part, including 1, 2 and 3 stacked MIA molecules (see Section S6.1) and the 6-31G* basis set. The QM part was allowed to fully relax while the remaining atoms were frozen to their initial positions. MOF structures were calculated with one MIA molecule as the QM part in the center of the clusters using the TZVP basis set. MIA and direct neighbors in the MM part were movable during optimization, while the rest of the cluster was kept fixed. In the case of MOF-5, the metal centers positions were additionally kept frozen.

Spectral properties were computed with the DFT/MRCI [87] program using the R2016 Hamiltonian [88]. Orbitals for MRCI were computed at the BHLYP/TZVP level using Turbomole [89]. The initial reference space was chosen by computing single and double excitations from eight electrons in eight orbitals. An energy selector of 0.8 *E_h_* and the *tight* parameter set were chosen. A second run was performed with the same parameters to obtain an updated reference space and the final values for energies and oscillator strengths.

## 4. Conclusions

The flavin derivatives 10-methyl-isoalloxazine (MIA) and 6-fluoro-10-methyl-isoalloxazine (6F-MIA) were successfully encapsulated in the metal-organic frameworks (MOFs) MIL-53 and MOF-5. The incorporation of flavin@MOF composites was verified by BET surface measurements and powder X-ray diffraction. In the case of the flavin@MOF-5 composites, the incorporation was also demonstrated spectroscopically by homogeneous images obtained by confocal laser scanning microscopy (Figure 3 and Figure 4). The photophysical properties of the flavin@MOF composites are significantly dependent on the specific molecular environment. Compared to the neat dyes in their solid states, the lifetime and quantum yield increase significantly in solution and in the dye@MOF composite. At least four fluorescent MIA species are present in the MOF composites. The lower lifetimes and quantum yield in the solid state of the dyes can be attributed to aggregation-caused quenching (ACQ). The fluorescence lifetimes and quantum yields of the flavins in liquid solution and in the “solid MOF solution” are of comparable orders. The electronic structures of MIA in its crystal environment, of MIA in MIL-53 and MIA in MOF-5 were investigated with plane-wave and QM/MM methods. In the crystalline state, π-stacking interactions of neighboring MIA chromophores change the nature of the first excited state from a local π-π (HOMO-LUMO) electronic excitation with high fluorescence intensity to a charge transfer state with low oscillator strength. The channels in the investigated metal-organic frameworks provide an environment for the incorporation of MIA as separated molecules, thus preventing π-stacking interactions between MIA molecules. The strong reduction of ACQ explains the enhanced fluorescence lifetime and quantum yield compared to the neat MIA crystal. The MIL-53 framework adapts towards the narrow-pore form when the MIA chromophore binds, thereby enhancing van-der-Waals contacts between the chromophore and the framework walls. Together with fluorescence anisotropy images and decays in Figure 6a,c, we can conclude that the transition dipole moments of the MIA derivatives are preferentially oriented along the one-dimensional channel axis. MOF-5 is a rather rigid framework but offers a narrower and wider pore environment for MIA with similar absorption and emission characteristics. In the preferred MIA position in the narrower pore, the hydrogen atoms of the phenyl linkers point towards the MIA-chromophore, thus enhancing host-guest van-der-Waals interactions. Together with fluorescence anisotropy images and decays in Figure 6b,d, we can conclude that the transition dipole moments of the MIA derivatives are not preferentially oriented along the three-dimensional cubic lattices but tilted in a similar manner (see Euler angles as described in Section 2.3).

The MOF environment influences the photophysical properties of the host chromophores. For 6F-MIA the non-radiative processes are significantly reduced. For MIA the location within the crystal becomes relevant. Further studies by fluorescence correlation spectroscopy (FCS) and transient state imaging microscopy (TRAST) will unravel the nature of these processes (internal conversion, IC or intersystem crossing, ISC). Moreover, we will also study the influence of the MOF environment on the photostability of the chromophores.

## Figures and Tables

**Table 1:** Results of nitrogen sorption measurements for MIL-53 and flavin@MIL-53.

Compound	Flavin Loading [wt%] *^a^*	*S*_BET_ [m^2^/g]	*V*_pore(total)_ [cm^3^/g] *^b^*	*V*_pore(micro;NLDFT)_ [cm^3^/g] *^c^*
MIL-53-lp literature	- -	1150 [61] 1140 [41]	0.53 n.a.	0.53 n.a.
MIL-53-lp synthesized	-	1130	0.45	0.43
MIA@MIL-53	0.4	1290	0.52	0.50
	0.8	1130	0.47	0.46
	1.6	1000	0.43	0.43
	2.0	890	0.41	0.42
	2.7	340	0.17	0.20
	3.9	80	0.05	0.09
6F-MIA@MIL-53	0.1	1150	0.49	0.49
	0.5	890	0.41	0.42
	1.0	600	0.28	0.30
	1.5	40	0.02	0.04

*^a^* Calculated by UV-VIS from initial flavin concentration. *^b^* Total pore volume at p/p_0_ = 0.50 for pores ≤2 nm. *^c^* Micropore volume calculated from NLDFT for pores ≤2 nm.

**Table 2:** Probability *p* that a certain occupation number of flavins per site is confined in MOFs assuming a random distribution of flavins ^a^.

	MIA@MOF-5 0.85 wt%	6F MIA@MOF-5 5.2 wt%	MIA@MIL-53 3.9 wt%	6F MIA@MIL-53 1.5 wt%
*n_av_(flavins*/*site)*	0.029	0.18	0.3	0.1
*p(one)*	98.7%	92.0%	86.9%	95.5%
*p(two)*	1.3%	7.6%	12.1%	4.3%
*p(≥three)*	0.0%	0.4%	1.0%	0.1%

^a^: Site corresponds to a pore in MOF-5 and to a channel segment in MIL-53, respectively. Since the channel in MIL-53 is infinite, we cannot speak of “pores” as in MOF-5. Therefore, we define the length of a channel segment that is needed to host a MIA via the length of MIA molecule of ≈13 Å.

**Table 3:** Photophysical data for MIA, 6F-MIA and the flavin@MOF composites with *λ*_exc_ = 405 nm (TCSPC) or 440 nm (confocal MFIS) at room temperature (RT) unless indicated otherwise.

Compound	λ_F,max_ [nm] ^a^	τ_1_ (x_1_), τ_2_ (x_2_), τ_3_ (x_3_) [ns] ^b^	τ_F_ [ns] ^b^	τ_x_ [ns] ^b^	x_sq_ ^g^	*Φ*_F_ [%] ^c^
MIA in solution:						
DCM/MeOH (RT)	527 ^d^	10.2 (0.20), 6.1 (0.62), 2.9 (0.19)	7.1	6.3 ^d^		28 ^d^
DCM/MeOH (77 K) ^h^	487/513	7.5 (1)	7.5	7.5		54
water	524 ^e^	5.0 (1)	5.0	5.0 ^e^		22
MIA solid	555, theo: 540 ^f^	3.5 (0.01), 0.8 (0.12), 0.2 (0.87)	0.8	0.3		<2
0.4 wt% MIA@MIL-53	523	7.2 (0.22), 4.0 (0.51), 1.7 (0.27)	5.0	4.1		15
3.9 wt% MIA@MIL-53	527, theo: 509 ^f^	7.6 (0.17), 4.0 (0.51), 1.3 (0.32) 6.2 (0.13), 2.5 (0.33), 0.7 (0.53) ^g^	4.9 3.6 ^g^	3.7 2.1 ^g^	0.87 ^g^	11
0.85 wt% MIA@MOF-5	533, theo: 528 ^f^	9.5 (0.10), 4.2 (0.55), 1.3 (0.35) 8.6 (0.10), 3.2 (0.65), 1.3 (0.24) ^g^	5.1 4.7 ^g^	3.7 3.3 ^g^	0.79 ^g^	6
6F-MIA in solution:						
DCM/MeOH (RT)	530 ^d^		3.6	3.6 ^d^		13 ^d^
DCM/MeOH (77 K) ^h^	486/514	4.6 (0.17), 3.4 (0.83) 6.0 (0.41), 3.1 (0.59)	4.7	4.3		26
water	533 ^e^	3.6 (1) ^e^	3.6	3.6 ^e^		12 ^e^
6F-MIA solid	527	0.5 (0.01), 0.2 (0.05), 0.05 (0.94)	0.1	0.06		<2
0.1 wt% 6F-MIA@MIL-53	534	5.9 (0.13), 2.6 (0.46), 0.8 (0.41)	3.4	2.3		5
1.5 wt% 6F-MIA@MIL-53	538	6.7 (0.09), 2.5 (0.36), 0.6 (0.55) 6.7 (0.07), 2.4 (0.39), 0.8 (0.53) ^g^	3.5 3.2 ^g^	1.9 1.9 ^g^	0.87 ^g^	3
5.2 wt% 6F-MIA@MOF-5	510	7.0 (0.12), 3.2 (0.59), 0.8 (0.29) 6.2 (0.21), 2.6 (0.70), 1.1 (0.09) ^g^	4.1 4.0 ^g^	3.0 3.2 ^g^	0.79 ^g^	8

^a^: Maximum of the fluorescence wavelength. ^b^: Fluorescence lifetimes: τ(x_i_) lifetime of species *i* (fraction), τ_F_ fluorescence-weighted average lifetime, τ_x_ species-weighted average lifetime. Raw time-resolved fluorescence decays with fits and obtained parameters are shown in the supplementary materials Section S5 Figures S24–S33 except for the MFIS data marked by ^g^. ^c^: Fluorescence quantum yield. Precision ± 2%. ^d^: In DCM/MeOH 1:1 (DCM = dichloromethane). ^e^: Mono-exponential fits from time-correlated single photon counting by Reiffers et al. [17]. ^f^: Computations in this work. ^g^: From confocal multi-parameter fluorescence image spectroscopy (MFIS) with pulsed excitation at 440 nm and λ_em_ = 502–538 nm. Global fits to p- and s-polarized decays required 4 fluorescence lifetime components. The shortest lifetime component (<30 ps, close to the instrumental resolution but distinct from scatter due to its smaller anisotropy) was not considered in the calculation of the average lifetimes and amplitudes were rescaled to the bright species with *τ* > instrument response function (FWHM ≈ 0.5 ns). The non-rescaled amplitude fractions x_sq_ of the lifetime component with *τ_sq_* < 30 ps amount to 87% (dye@MIL-53) and 79% (dye@MOF-5) of all species, indicating a significant amount of strongly quenched dyes in both matrices. Raw time- and polarization-resolved fluorescence decays with fits and obtained parameters are shown in the supplementary materials Section S5 Figure S34. ^h^: Measured in a 1:1 DCM/MeOH glassy matrix at 77 K.

## Data Availability

The data presented in this study are available on request from the corresponding author.

## Supplementary Materials

The following supporting information can be downloaded at https://www.mdpi.com/article/10.3390/molecules28062877/s1. Section S1: Materials and methods; Section S2: MIA and 6F-MIA absorption spectra and calibration curves (Figures S4–S11); Section S3: Structure descriptions (Figures S12–S15); Section S4: Nitrogen adsorption isotherms and powder X-ray diffractograms (Figures S16–S19); Section S5: Photophysical characterization of the dyes and dye@MOF (Figures S20–S35); Section S6: Theoretical calculations (Figures S36–S42). References [90,91,92,93] are cited in the supplementary materials.

## Sample Availability

Samples of the compounds will not be available.
