# Supplementary material for: Enhanced Solid-State Fluorescence of Flavin Derivatives by Incorporation in the Metal-Organic Frameworks MIL-53(Al) and MOF-5
Source: Molecules. 2023 Mar 22;28(6):2877. doi: 10.3390/molecules28062877 (PMC10055669; doi:10.3390/molecules28062877)
Supplement: Supplementary file 1 [file molecules-28-02877-s001.zip › molecules-2210143-supplementary.pdf]

Supplementary Material

Enhanced solid-state fluorescence of flavin derivatives by incorporation in the metal-organic frameworks MIL-53(Al) and MOF-5

Dietrich Püschel [1], Simon Hédé [2], Iván Maisuls [3], Simon-Patrick Höfert [1], Dennis Woschko [1], Ralf Kühnemuth [4], Suren Felekyan [4], Claus A. M. Seidel [4,*], Constantin Czekelius [5], Oliver Weingart [2,*], Cristian A. Strassert [3,*] and Christoph Janiak [1,*]

1 Institut für Anorganische Chemie und Strukturchemie, Heinrich-Heine-Universität Düsseldorf, Universitätsstraße 1, D-40225 Düsseldorf, Germany

2 Institut für Theoretische Chemie und Computerchemie, Heinrich-Heine-Universität Düsseldorf, Universitätsstraße 1, D-40225 Düsseldorf, Germany

3 Institut für Anorganische und Analytische Chemie, CeNTech, CiMIC, SoN, Westfälische Wilhelms-Universität Münster, Heisenbergstraße 11, D-48149 Münster, Germany

4 Institut für Physikalische Chemie, Heinrich-Heine-Universität Düsseldorf, Universitätsstraße 1, D-40225 Düsseldorf, Germany

5 Institut für Organische Chemie und Makromolekulare Chemie, Heinrich-Heine-Universität Düsseldorf, Universitätsstraße 1, D-40225 Düsseldorf, Germany

Corresponding authors:
E-mails: Cseidel@hhu.de, Oliver.Weingart@hhu.de, ca.s@wwu.de, janiak@uni-duesseldorf.de;
Tel: +49-211-81-12286

Additional E-mail addresses:
Dietrich.Pueschel@hhu.de; Simon.Hede@hhu.de; Maisuls@uni-muenster.de;
Simon.patrick.hoefert@googlemail.com; Dennis.Woschko@hhu.de; Ralf.Kuehnemuth@hhu.de;
Suren.Felekyan@uni-duesseldorf.de, Cseidel@hhu.de ; Oliver.Weingart@hhu.de;
Constantin.Czekelius@hhu.de; ca.s@wwu.de

Table of Contents

Section S1: Materials and methods 3

Section S1.1: Synthesis of flavins 3

Section S1.2: MOF syntheses and flavin incorporation 3

Section S2: MIA and 6F-MIA absorption spectra and calibration curves (Figure S4-Figure S11) 11

Section S3: Structure descriptions (Figure S12 - Figure S15) 15

Section S3.1: Structure of 10-methyl-isoalloxazine (MIA) (Figure S12) 15

Section S3.2: Additional structure description of MIL-53(Al) (Figure S13 and Figure S14) 15

Section S3.3: Additional structure description of MOF-5 (Figure S15) 17

Section S4: Nitrogen adsorption isotherms and powder X-ray diffractograms (Figure S16 - Figure S19) 19

Section S5: Photophysical characterization of the dyes and dye@MOF (Figure S20 - Figure S35) 24

Section S6: Theoretical calculations (Figure S36 - Figure S42) 33

Section S6.1: Force Field MIA 33

Section S6.2: Force Field MIL-53(Al) 36

Section S6.3: Force Field MOF-5 39

Section S6.4: QM part, MM part 42

Section S6.5: Transition dipole moments of MIA 47

Section S1: Materials and methods

Commercially available reagents (Table S1) were used without further purification. All reagents were used in reagent grade without further purification. The solvents were purchased in reagent grade or purified by conventional methods. For reactions requiring an inert atmosphere the glassware was dried in a compartment dryer at 120 °C and standard Schlenk techniques were used to work under a dry nitrogen atmosphere. Degassing of solvents was done by purging with dry nitrogen for 30 minutes.

Table S1. Sources for starting materials and solvents.

Reagent	Manufacturer
Zinc nitrate hexahydrate	ACROS Organics
Terephthalic acid	Alfa Aesar
Ethanol	CHEMSOLUTE
N,*N*-dimethylformamide	Honeywell
N,*N*-diethylformamide	Honeywell
Aluminum nitrate nonahydrate	CARL ROTH

Section S1.1: Synthesis of flavins

Flavins were prepared from the corresponding 2-methylamino-anilines by condensation with alloxan hydrate. *N*-Methylation of 2-nitro-anilines was performed by alkylation of the corresponding trifluoroacetamides with dimethylsulfate following a procedure by Brown and Rizzo [1]. 10-Methyl-isoalloxazine (MIA) (Scheme S1) was prepared following a protocol reported by Imada, Murahashi and coworkers [2]. 6-Fluoro-10-methyl-isoalloxazine (6F-MIA) (Scheme S1) [3] was prepared following a modified protocol reported by Averill and co-workers [4].

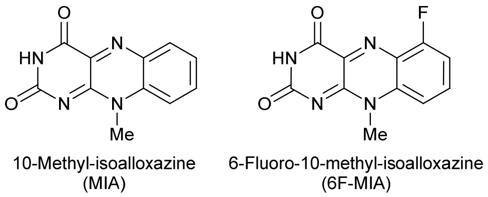

Scheme S1. 10-Methyl-isoalloxazine (MIA) and 6-fluoro-10-methyl-isoalloxazine (6F-MIA).

Section S1.2: MOF syntheses and flavin incorporation

Preparation of saturated MIA and 6F-MIA dye solutions

The MIA or 6F-MIA dye was mixed in chloroform or DMF and dissolved for 30 min at 40 °C using ultrasound. When a clear homogeneous solution was obtained, this process was repeated until no more dye could be dissolved. The saturated chloroform solutions (c_{MIA} = 69.5 mg·L^{-1}, $c_{6F\text{-}MIA}$ = 20.25 mg·L^{-1}; see Section S2) or DMF solutions (c_{MIA} = 272 mg·L^{-1}, $c_{6F\text{-}MIA}$ = 2640 mg·L^{-1}; see Section S2) were then filtered at room temperature and used for the syntheses.

Synthesis of MIL-53(Al) according to Alaerts et al. and Loiseau et al. [5,6]

Aluminum nitrate nonahydrate (3.75 g, 10.0 mmol), terephthalic acid (0.83 g, 5.0 mmol) and dist. water (7.16 mL) were mixed in a 60 mL Teflon-lined stainless-steel autoclave, which was heated at 220 °C for 72 h including a 3 h heating ramp and 3 h cooling ramp. After cooling to room temperature, the white raw product was washed three times with water (10 mL). The white solid was dispersed in DMF and heated under reflux to 160 °C for 48 h to remove the incorporated terephthalic acid from the pores. The product was finally washed three times with hot ethanol (10 mL each). Yield 46 %.

10-Methyl-isoalloxazine (MIA) and 6-fluoro-10-methyl-isoalloxazine (6F-MIA) incorporation into MIL-53

Post-synthetic wet infiltration was performed by adding 1 to 15 mL from the concentrated solutions of the dyes in chloroform to 20 mg activated powders of the microcrystalline MIL-53. After brief mixing, slow evaporation at room temperature was used to promote diffusion of the dyes into the MOF pores.

MOF-5 single crystal synthesis according to Han et al. and Eddaoudi et al. [7,8]

Zinc nitrate hexahydrate (511 mg, 1.72 mmol) and terephthalic acid (96 mg, 0.58 mmol) were dissolved in dimethylformamide, DMF (5 mL). The solution was transferred to Pyrex tubes, which were placed in a programmable oven at 85 °C for 72 h including a 2.5 h heating ramp and 3.5 h cooling ramp. The colorless crystals obtained were washed with DMF (5 mL each) twice daily for three days and with ethanol (5 mL each) twice daily for another three days. After washing, the MOF-5 crystals were filtered and dried under vacuum (15 h at 120 °C). Yield 58 %. The MOF-5 were stored under nitrogen until further investigations.

MOF-5 single crystal synthesis with 10-methyl-isoalloxazine (MIA)

MIA (5.0 mg, 0.022 mmol) was added to DMF (5 mL) and sonicated at 60 °C for 30 min. The solution was then filtered to obtain a homogeneous MIA-saturated DMF solution with c_{MIA} = 272 mg·L^{-1} (Section S2). Zinc nitrate hexahydrate (175.5 mg, 0.59 mmol) and terephthalic acid (31.0 mg, 0.19 mmol) were dissolved in the MIA-DMF solution (5 mL). The solution was then placed in a programmable oven at 85 °C for 72 h including a 2.5 h heating ramp and 3.5 h cooling ramp. Yellow discolored cubic crystals were obtained. The crystals were washed three times with DMF (5 mL), filtered and dried under vacuum (15 h at 120 °C). Yield 42.3 mg (MIA@MOF-5), 39.9 mg MOF-5 after deduction of MIA content (see below), ~90% MOF-5 based on terephthalic acid. The dry MIA@MOF-5 composites were stored under nitrogen until further investigations. For longer storage (> 4 weeks), the non-washed crystals were stored in the mother liquor at 4 °C. When needed, the crystals were then worked up as described above.

The 10-fold diluted supernatant DMF solution gave an absorption at 436 nm of 0.892 from which a residual MIA concentration of 0.892/0.04467 = 19.97 mg L^{-1} (Figure S5), thus 199.7 mg L^{-1} for the original supernatant could be obtained. Hence, from 1.36 mg of MIA in 5 mL of the saturated MIA-DMF solution 1.00 mg of MIA was still present in the supernatant after MOF-5 formation or 0.36 mg MIA were incorporated in 39.94 mg MOF-5 (42.3 mg – 0.36 mg), representing 0.36 / (0.36+39.94) = 0.85 wt%.

MOF-5 single crystal synthesis with 6-fluoro-10-methyl-isoalloxazine (6F-MIA)

6F-MIA (20 mg, 0.081 mmol) was added to DMF (5 mL) and sonicated at 60 °C for 30 min. The solution was then filtered to obtain a homogeneous 6F-MIA-saturated DMF solution with $c_{6F\text{-}MIA}$ = 2640 mg·L^{-1} (Section S2). Zinc nitrate hexahydrate (175.5 mg, 0.59 mmol) and terephthalic acid (31.0 mg, 0.19 mmol) were dissolved in the 6F-MIA-DMF solution (5 mL). The solution was then placed in a programmable oven at 85 °C for 72 h including a 2.5 h heating ramp and 3.5 h cooling ramp. Yellow discolored cubic crystals were obtained. The crystals were washed three times with DMF (5 mL each), filtered and dried under vacuum (15 h at 120 °C). Yield 40.5 mg (6F-MIA@MOF-5), 29.3 mg MOF-5 after deduction of 6F-MIA, ~57% MOF-5 based on terephthalic acid.

The dry 6F-MIA@MOF-5 composites were stored under nitrogen until further investigations. For longer storage (> 4 weeks), the non-washed crystals were stored in the mother liquor at 4 °C. When needed, the crystals were then worked up as described above.

The 20-fold diluted supernatant DMF solution gave an absorption at 425 nm of 0.544 from which a residual 6F-MIA concentration of 0.544/0.0272 = 20.0 mg L^{-1} (Figure S9), thus 400 mg L^{-1} for the original supernatant could be obtained. Hence, from 13.2 mg of 6F-MIA in 5 mL of the saturated MIA-DMF solution 2.0 mg of 6F-MIA was still present in the supernatant after MOF-5 formation or 11.2 mg MIA were adsorbed onto or incorporated in 29.3 mg MOF-5 (40.5 mg – 11.2 mg), representing 11.2 / (11.2+29.3) = 27.6 wt%, albeit before the washing process three times with DMF (5 mL each).

However, digestive dissolution in DMSO-d_6/DCl of 6F-MIA@MOF-5 gave a loading of 5.2 wt%.

The MIA@MOF-5 and 6F-MIA@MOF-5 composites were dissolved in 0.8-0.9 mL DMSO-d_6 upon addition of ~0.1 mL 35% DCl/D_2O. The solution ^{1}H NMR spectra were recorded on a Bruker Avance III-300 MHz spectrometer. Chemical shifts are referenced to the residual proton solvent signal (2.50 ppm for DMSO-d_6).

The digestion ^{1}H NMR spectrum of 6F-MIA@MOF-5 (Figure S1a) shows a small signal for the N-methyl group of 6F-MIA (cf. Scheme S1).

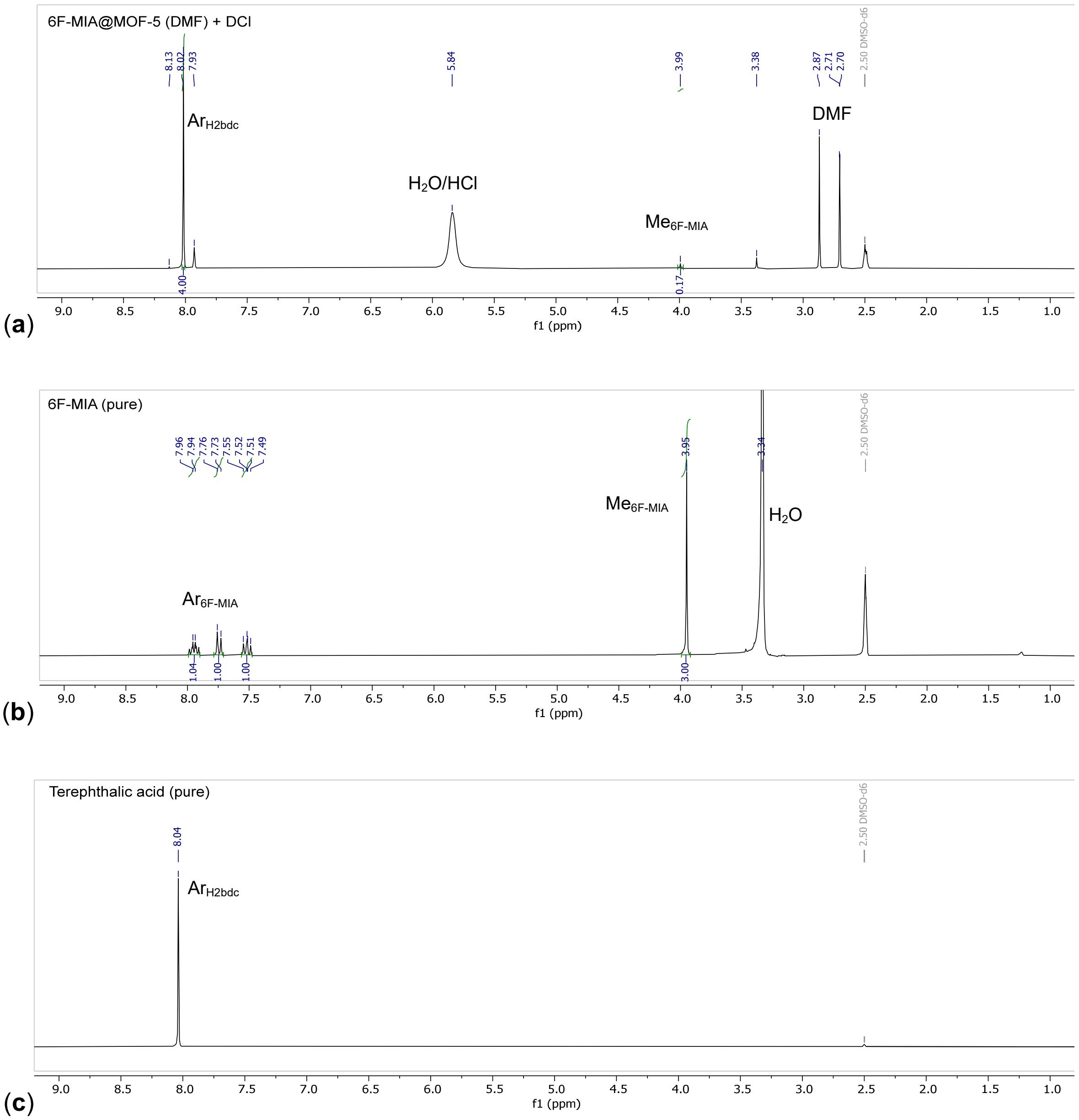

Figure S1. ^{1}H NMR spectra in DMSO-d_6 at 300 MHz of (**a**) 6F-MIA@MOF-5 upon digestive dissolution with DCl/D_2O, (**b**) 6F-MIA and (**c**) terephthalic acid.

The integral of 4.00 for the terephthalic acid signal (4 aromatic protons) has to be divided by 12 as there are three terephthalate (bdc) linkers in the $[Zn_4O(bdc)_3]$ formula unit of MOF-5. The integral of 0.17 for the N-methyl group (3 protons) of 6F-MIA has to be divided by 3. This normalization then gives the molar ratio of MOF-5 formula units to 6F-MIA molecules:

4/12 : 0.17/3 = 1:0.17 = 5.88 mol $[Zn_4O(bdc)_3]$ for each mol of 6F-MIA.

With M = 769.87 g/mol for $[Zn_4O(bdc)_3]$ and M = 246.20 g/mol for 6F-MIA

there are 246.20 g 6F-MIA per 5.88 mol × 769.87 g/mol = 4528.6 g MOF-5.

This gives a 6F-MIA@MOF-5 loading of 246.20 / (246.20 + 4528.6) × 100% = 5.2 wt%.

In view of the much smaller content of MIA@MOF-5 of at most 0.85 wt% from UV/Vis difference measurements no N-methyl signal is seen in the digestion ^{1}H NMR spectra of MIA@MOF-5.

As an explanation of the obtained large loading of 6F-MIA from the UV/Vis absorption difference measurement we suggest that a sizeable amount of 6F-MIA from the concentrated solution was adsorbed on the outer surface or in the pore mouths. This 6F-MIA content could easily be removed when the crystals were washed three times with DMF (5 mL). The much better solubility of 6F-MIA with $c_{6F\text{-}MIA}$ = 2640 mg·L^{-1} compared to MIA with only c_{MIA} = 272 mg·L^{-1}, both for saturated solutions, will also lead to a more facile postsynthetic removal of 6F-MIA through the washing cycles.

Infrared (IR) spectra of flavin@MOF

The Fourier transform infrared (FT-IR) spectra were collected with a Bruker Tensor 37 instrument with KBR or attenuated total reflection (ATR) technique. The spectra are collected in Figure S2 and Figure S3.

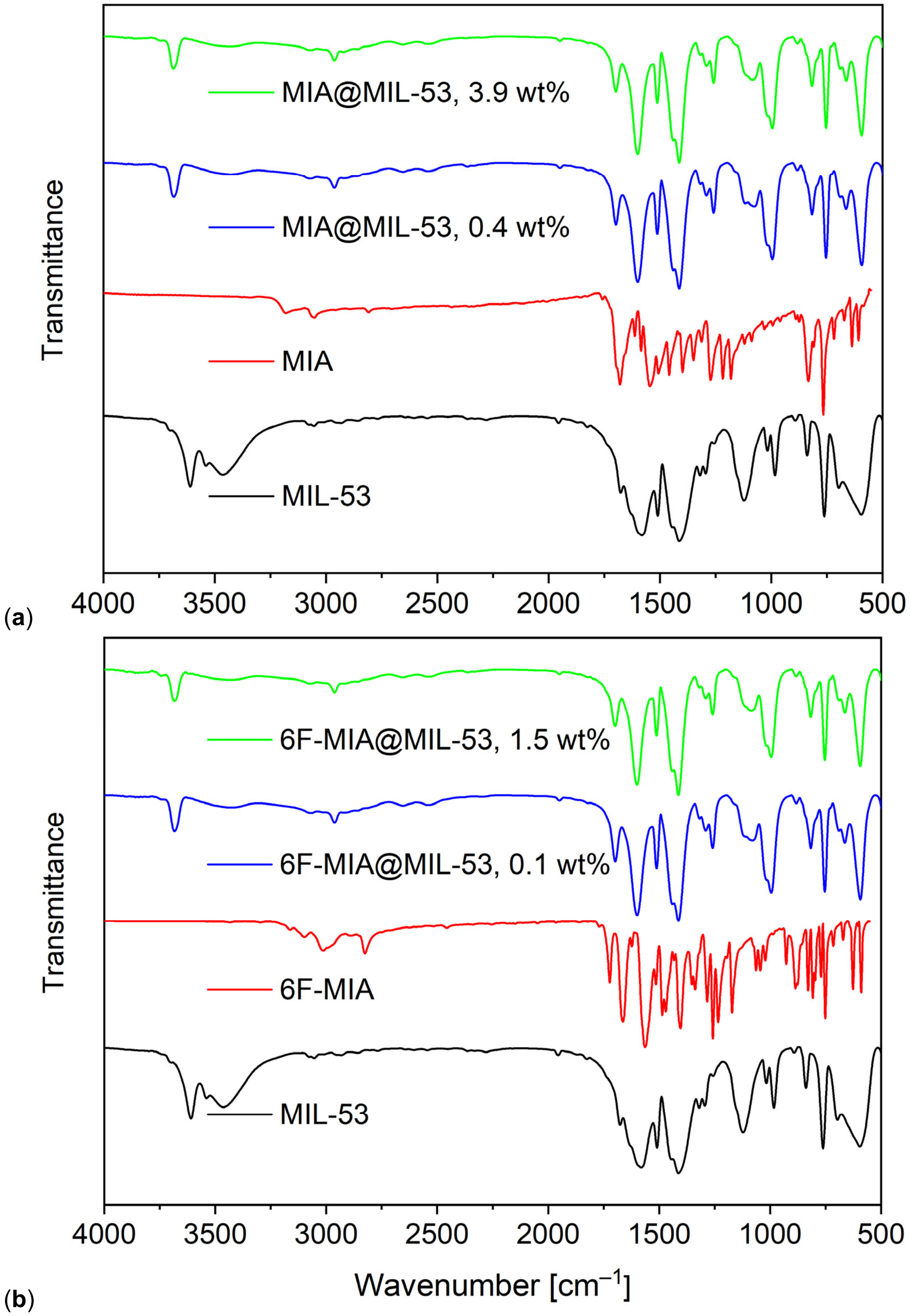

Figure S2. IR spectra of (**a**) MIL-53 (KBr), MIA (ATR) and MIA@MIL-53 (KBr) and (**b**) MIL-53 (KBr), 6F-MIA (ATR) and 6F-MIA@MIL-53 (KBr).

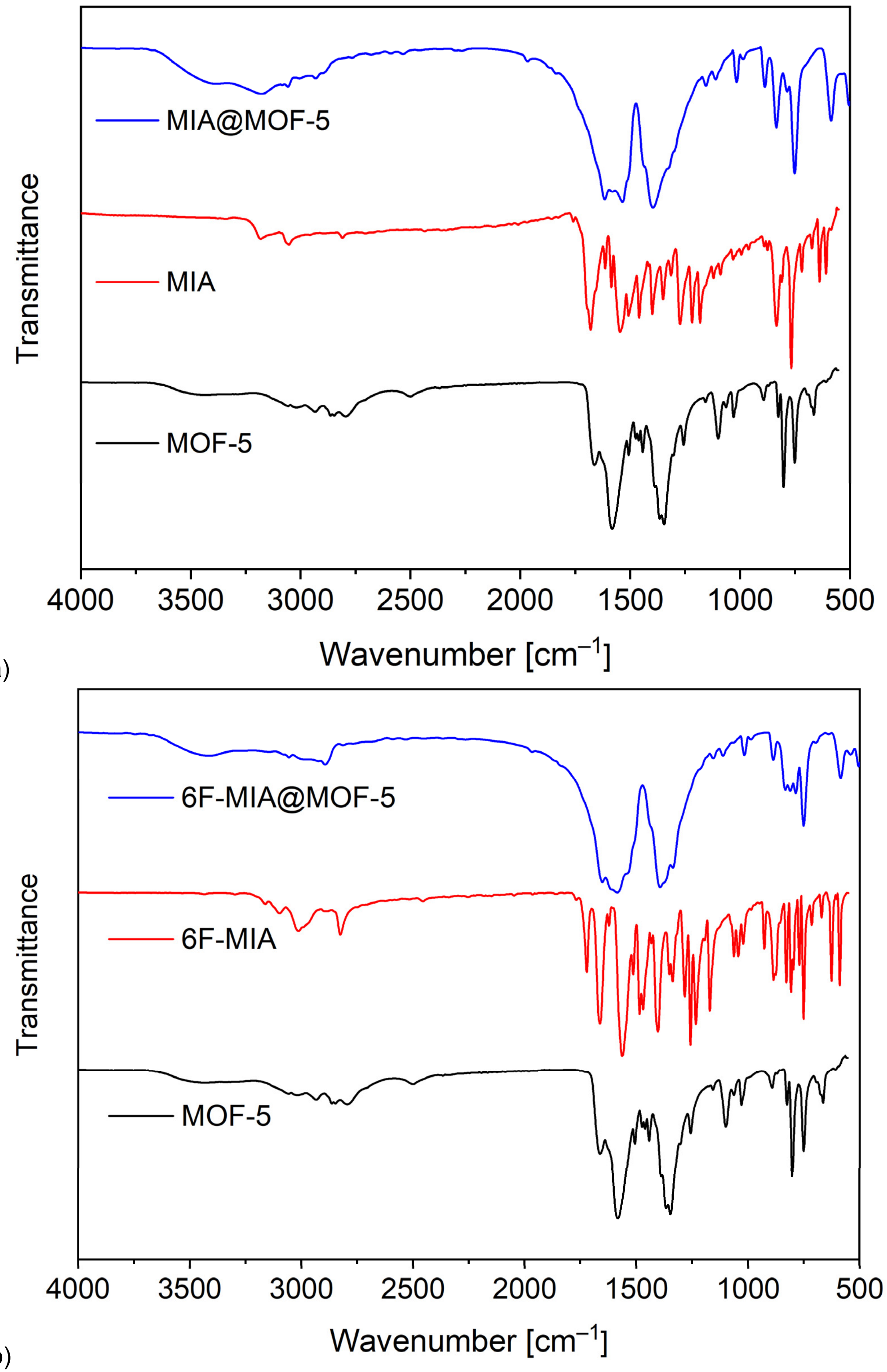

(**a**)

(**b**)

Figure S3. IR spectra of (**a**) MOF-5 (KBr), MIA (ATR) and MIA@MOF-5 (KBr) and (**b**) MOF-5 (KBr), 6F-MIA (ATR) and 6F-MIA@MOF-5 (KBr).

Neat MIL-53 features broad signals which we see as evidence for the superposition of the three MIL-53 phases MIL-53-as, MIL-53-np and MIL-53-lp (and intermediate phases) as is also evident from

the powder X-ray diffractograms (cf. Figure S14a). In the flavin@MIL-53 composites the bands sharpen and become more distinct with all composites giving nearly identical spectra (Figure S2). This is due to the formation of the same phase from the sample preparation as KBr pellets. The superposition of the flavin bands and the low amounts of flavins in MIL-53 do not allow a detection of flavin bands in the composites.

MOF-5 on the other hand has more distinct bands in its neat form whereas in the composites the band between 1000 and 1750 cm^{-1} have considerably broadened (Figure S3). Distinct bands remain only in the region between 750 to 1000 cm^{-1} where C=C- and C-H-bending vibrations occur. Also, MIA and 6F-MIA have bands in the same region. Upon close inspection the flavin-only bands at 606 and 833 cm^{-1} (MIA) and 588 and 828 cm^{-1} (6F-MIA) are also localized in the composites (Table S2).

Table S2. Listing of IR wavenumbers of MOF-5, MIA, 6F-MIA and flavin@MOF-5 composites.

Wavenumber [cm^{-1}]	MOF-5 (KBr)	MIA (ATR)	MIA@MOF-5 (KBr)	6F-MIA (ATR)	6F-MIA@MOF-5 (KBr)
C=C bending, C-H bending		606 ++	590 ++	588 ++	586 ++
	660 +	636 ++		625 ++	
	750 ++	765 +++	751 +++	750 +++	750 +++
	800 +++			808 ++	810 ++
				828 ++	835 ++
		833 ++	837 ++		
	892 +		889 +	885 ++	887 +
	1025 +		1012 +		1014 +
	1096 ++				
C-H stretching, O-H bending	1250-1450 +++, b (1343)	1216 ++	1230-1470 +++, b (1390)	1257 +++	1250-1470 +++, b (1390)
C-H/O-H stretching	1450-1670 +++, b (1578)	1454 +++	1470-1750 +++, b (1531,1616)	1564 +++	1480-1750 +++, b (1585)
		1680 ++		1722 ++	
	2495 +				
C-H/O-H stretching	2790-3100 +, b	2800-3180 +, b	2925-3530 +, b (3175)	2823 +	2900-3400 +, b
				3010 +	

Section S2: MIA and 6F-MIA absorption spectra and calibration curves (Figure S4-Figure S11)

For the concentration determination of the saturated solution of MIA in DMF, a stock solution with a concentration of 100 mg·L^{-1} MIA in DMF was prepared, diluted to lower concentrations and measured by UV/Vis spectroscopy (Figure S4).

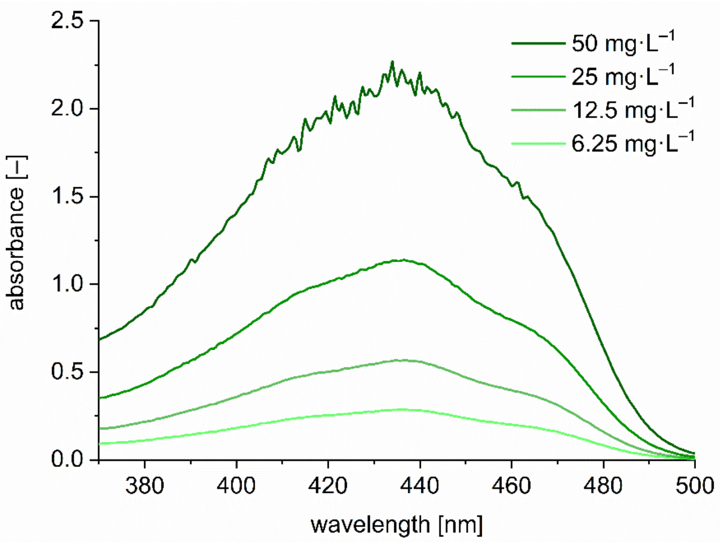

Figure S4. UV/Vis spectra of differently concentrated solutions of MIA in DMF in the range from 370 to 500 nm.

Using the maxima of the absorption spectra at 436 nm, a calibration curve was derived (Figure S5).

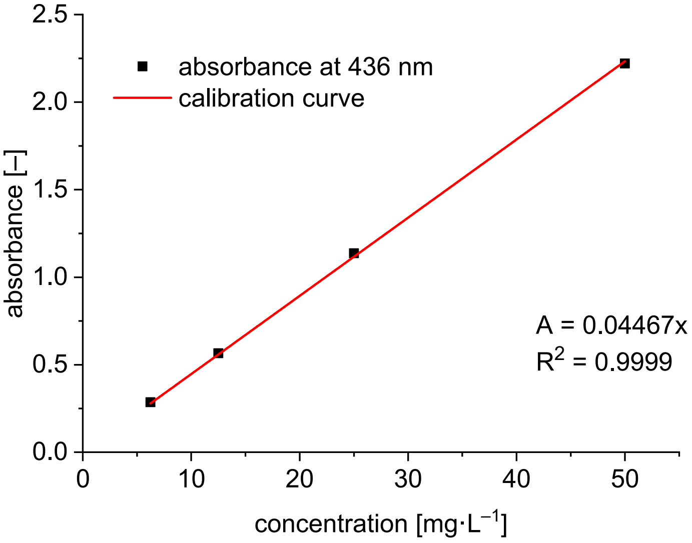

Figure S5. Calibration line for MIA in DMF from the absorbance at 436 nm.

The concentration of the **saturated MIA-DMF solution** was determined as follows: The saturated MIA-DMF solution was diluted by a factor of 1:10 to obtain an absorbance at 436 nm of 1.214 within the calibration range giving a concentration of 27.2 mg L^{-1} for the tenfold-diluted solution. Taking this dilution into account, a concentration of 272 mg·L^{-1} was calculated for the saturated MIA-DMF solution.

For the concentration determination of the saturated solution of MIA in chloroform, a stock solution of 4 mg·L^{-1} of MIA in chloroform was prepared and diluted. This was followed by the recording of the UV/Vis spectra of the solution series (Figure S6).

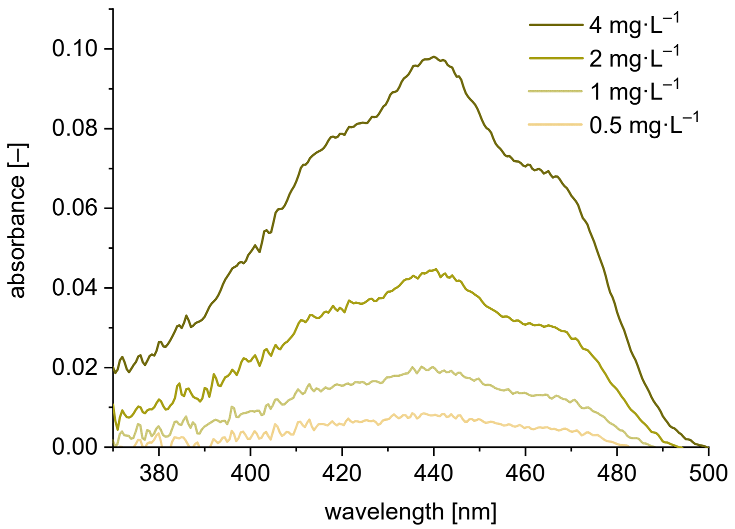

Figure S6. UV/Vis spectra of differently concentrated solutions of MIA in chloroform in the range from 370 to 500 nm.

From the absorbance maxima at 440 nm the calibration curve was obtained (Figure S7).

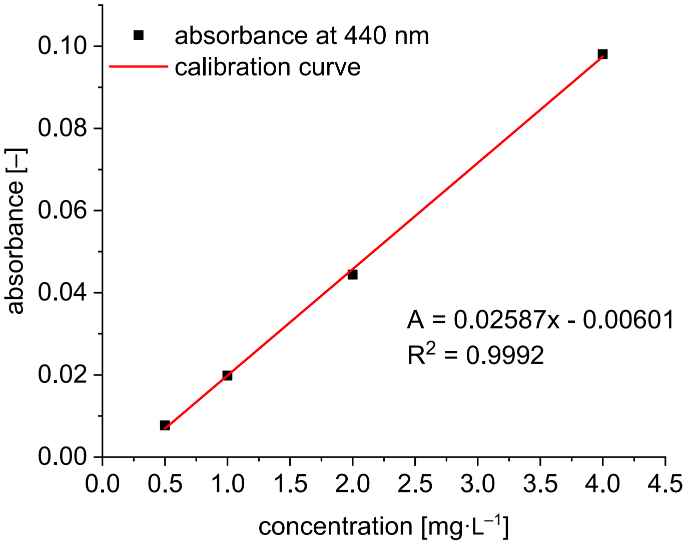

Figure S7. Calibration line for MIA in chloroform from the absorbance at 440 nm.

Subsequently, the concentration of the **saturated MIA-CHCl$_3$ solution** was determined as follows: The saturated MIA-CHCl$_3$ solution was diluted by a factor of 1:25 to obtain an absorbance at 440 nm of 0.078 within the calibration range giving a concentration of 2.78 mg L^{-1} for the 25-fold-diluted solution. Taking this dilution into account, a concentration of 69.5 mg·L^{-1} was calculated for the saturated MIA-CHCl$_3$ solution.

To determine the concentration of the saturated solution of 6F-MIA in DMF, a stock solution with a concentration of 100 mg·L^{-1} of 6F MIA in DMF was prepared and the UV/Vis-spectra were recorded for the dilution series (Figure S8).

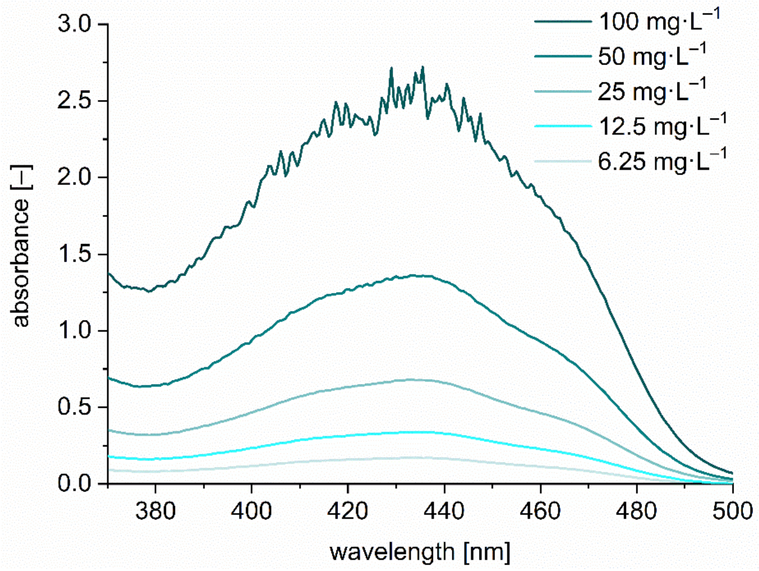

Figure S8. UV/Vis spectra of different concentrated solutions of 6F-MIA in DMF in the range from 370 to 500 nm.

From the absorbance maxima at 435.5 nm the calibration curve was obtained (Figure S9).

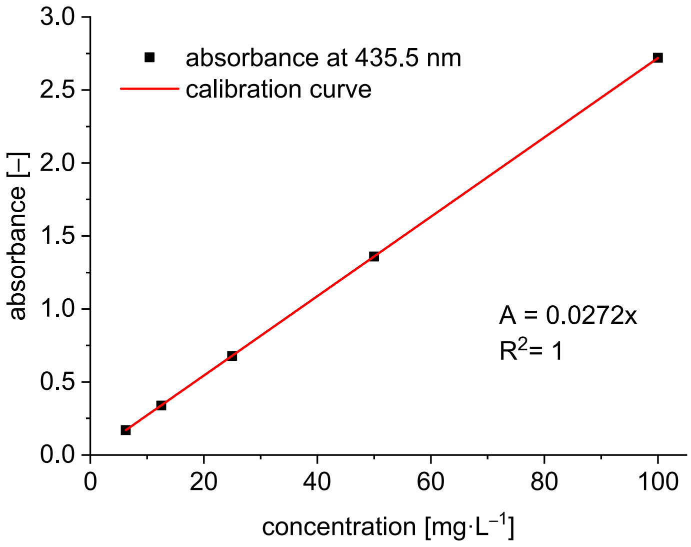

Figure S9. Calibration line for 6F-MIA in DMF from the absorbance at 435.5 nm.

The concentration of the **saturated 6F-MIA-DMF solution** was determined as follows: The saturated 6F-MIA-DMF solution was diluted by a factor of 1:50 to obtain an absorbance at 435.5 nm of 1.436 within the calibration range giving a concentration of 52.8 mg L^{-1} for the 50-fold-diluted solution. Taking this dilution into account, a concentration of 2640 mg·L^{-1} was calculated for the saturated 6F-MIA-DMF solution.

The concentration of the saturated solution of 6F-MIA in chloroform was determined by starting from a solution with a concentration of 2.4 mg·L^{-1} 6F MIA in chloroform, from which a dilution series was prepared and the UV/Vis spectra were recorded (Figure S10).

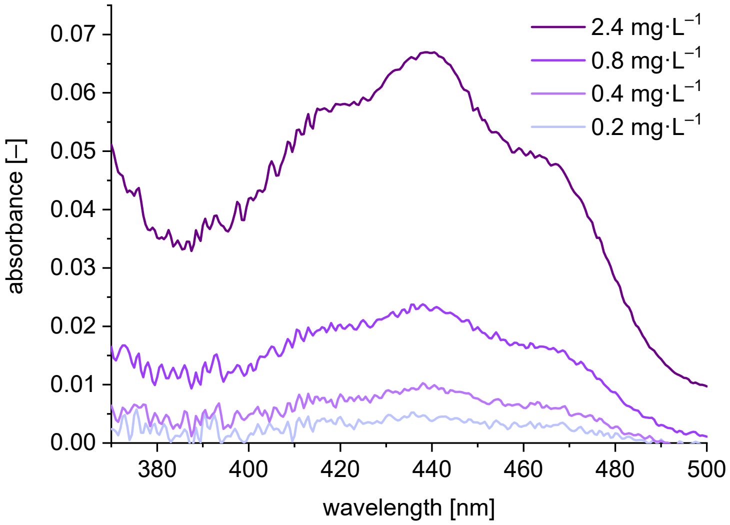

Figure S10. UV/Vis spectra of differently concentrated solutions of 6F-MIA in chloroform in the range from 370 to 500 nm.

The calibration line was derived from the maxima of the absorption at 440 nm (Figure S11).

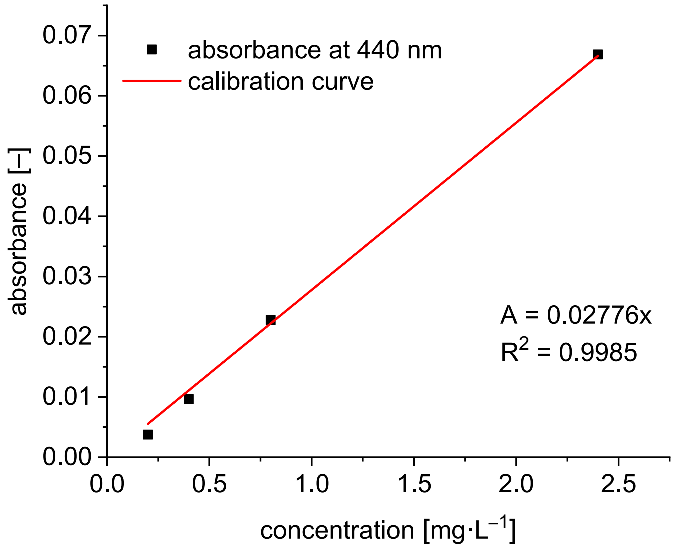

Figure S11 Calibration line for 6F-MIA in chloroform from the absorbance at 440 nm.

Subsequently, the **saturated 6F-MIA-$CHCl_3$ solution** was diluted by a factor of 1:12.5 to obtain an absorbance at 440 nm of 0.045 within the calibration range giving a concentration of 1.62 mg L^{-1} for the 12.5-fold-diluted solution. Taking this dilution into account, a concentration of 20.25 mg·L^{-1} was determined for the saturated 6F-MIA-$CHCl_3$ solution.

Section S3: Structure descriptions (Figure S12 - Figure S15)

Section S3.1: Structure of 10-methyl-isoalloxazine (MIA) (Figure S12)

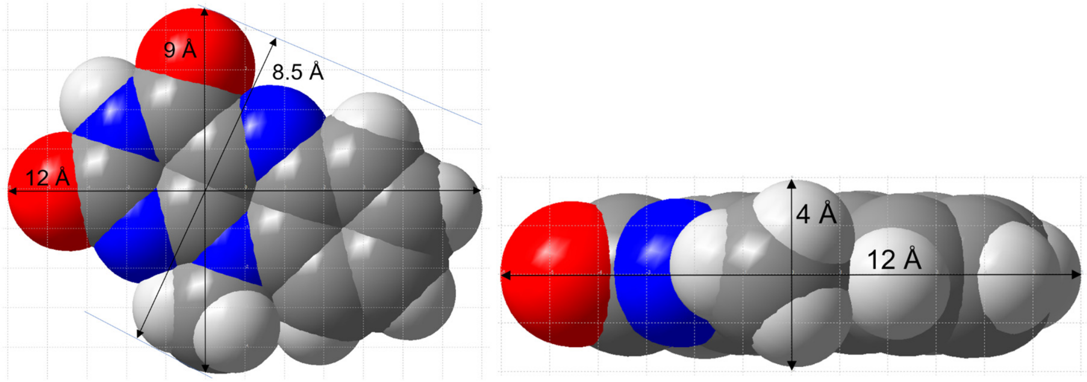

Figure S12. Top view and side view of a 10-methyl-isoalloxazine (MIA) molecule in space-filling presentation with the molecular dimensions indicated (the grid unit is 1 Å). (Structures were drawn with Diamond [9] from the deposited cif file under CCDC-no./Refcode MISALX [10]).

Section S3.2: Additional structure description of MIL-53(Al) (**Figure S13 and Figure S14**)

The MIL-53(Al) structure with the formula unit [Al(OH)(bdc)] (bdc = benzene-1,4-dicarboxylate, terephthalate) is a very flexible, 'breathing'-type network, that is, it can assume different shapes and porosities depending on the presence or absence of host-guest interactions (Figure S13) [5,11]. The secondary or here infinite building unit (SBU, IBU) in MIL-53 is a linear chain of $\{MO_6\}$ octahedra (M = Al, In, Ga, Cr, Fe) The Al^{3+} centers are octahedrally coordinated by terephthalate linkers and bridging μ-OH groups, giving chains of trans-μ-OH-connected vertex-bridged $\{AlO_6\}$ octahedra. The hydroxido-bridging and carboxylate-bridging occurs along the direction of the metal chains (Figure S13a). These chains are connected perpendicular to the chain direction through the ditopic terephthalate linkers to a three-dimensional network with channels running parallel to the $\{MO_6\}$ chains (Figure S13b). Each benzene-1,4-dicarboxylate ligand bridges between four Al atoms. This forms a three dimensional network consisting of rhombic-shaped channels with a significant breathing effect [5,11,12,13,14,15,16,17,18].

MIL-53 can be found in three different crystalline forms which are usually differentiated as MIL-53*as*, MIL-53*ht* = MIL-53*lp* and MIL-53*lt* = MIL-53*np* with *as* = *as synthesized*, *ht* = *high temperature lp* = *large pore*, *lt* = *low temperature* and *np* = *narrow pore* (Figure S13). After the hydrothermal synthesis as-synthesized MIL-53-as is obtained. By heating to a temperature between 275 °C and 420 °C (without vacuum), MIL-53 can be activated by the removal of guest molecules. This leads to the large-pore (lp) structure MIL-53-lp. Upon the uptake of water molecules from the air at ambient conditions, the narrow-pore (np) MIL-53-np is formed (Figure S13). The transformation between MIL-53-lp to MIL-53-np is reversible and is commonly referred to as the ‘breathing effect’. For the large-pore (lp) form the diagonals for the rhombohedral channel cross section are 8.4 x 12 Å (Figure S14).

When incorporating the flavin derivatives, the MIL-53-lp form can be used as the van-der-Waals dimensions of MIA molecules are approximately 4 × 8.5 × 12 Å (Figure S12) with the width of 8.5 Å being the critical dimension for the diffusion through the rhombohedral MIL-53 channels.

(**a**)

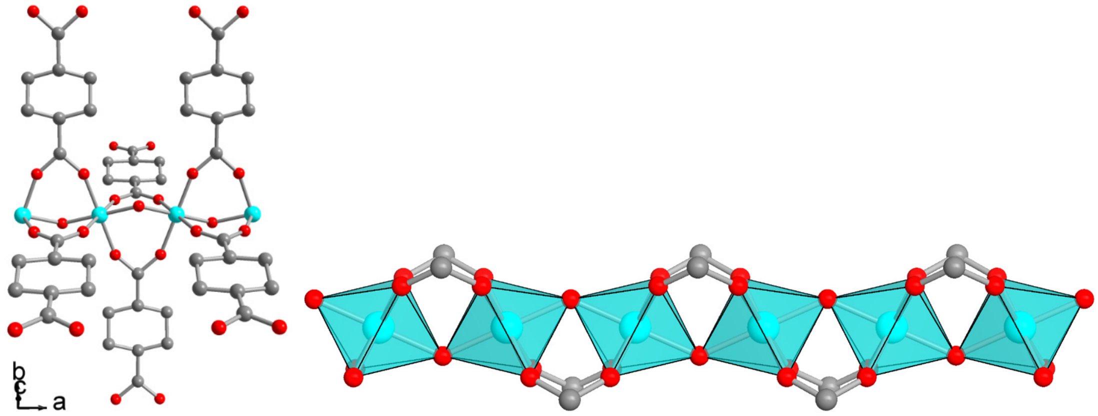

(**b**)

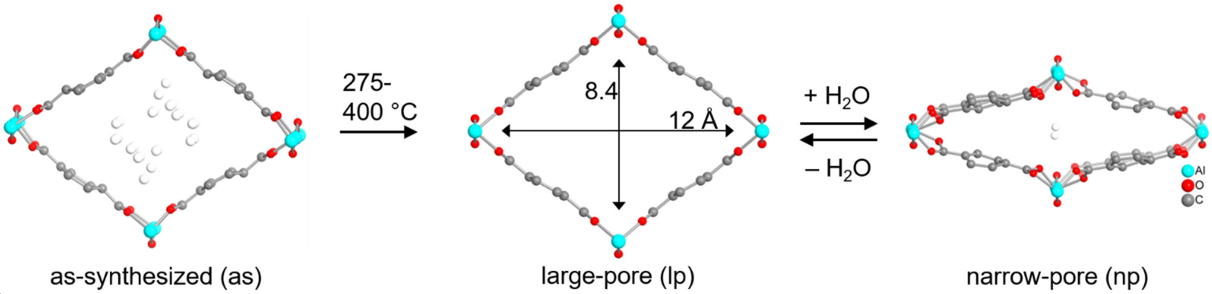

Figure S13. (**a**) Terephthalato- and hydroxido-bridged metal strand of [Al(OH)(bdc)] as an infinite subunit. (**b**) Sections of the packing diagram with a flexible, 'breathing' network adapting to guest molecules. The pore structures of MIL-53(Al) are viewed along the channel direction. The channels can contain guest molecules in the as-synthesized or narrow-pore form or be empty in the similar large-pore structure. The as-synthesized, and the narrow-pore form also indicate the (disordered) guest molecules (hydrogen atoms are not shown). The high temperature for the transition from as-synthesized to large-pore form is due to the removal of residual terephthalic acid. The dimensions along the diagonals of the rhombic opening include the van-der-Waals radii of the atoms (see also Figure S14). Adsorption of water from air at room temperature transforms the structure into the narrow-pore / low-temperature form (right). Hydrogen atoms are not shown. (Structure images were drawn with Diamond [9] from the deposited cif files under CCDC-no./CSD-Refcodes 220475/SABVOH for MIL-53-as, 220476/SABVUN for -MIL-53-lp and 220477/SABWAU for MIL-53-np [6]).

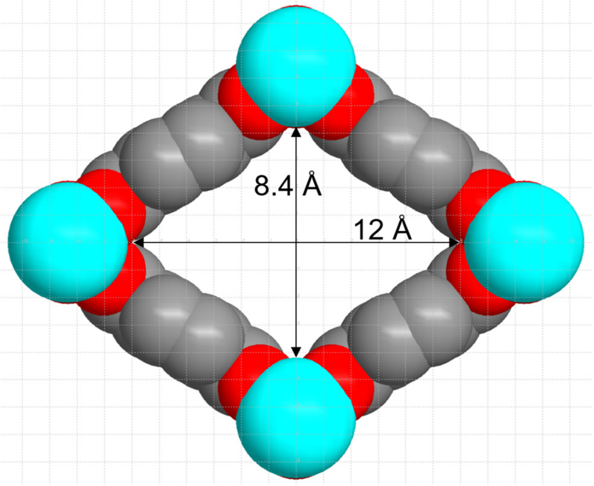

Figure S14. MIL-53(Al)-lp (large pore) in space-filling mode viewed along the channel direction. The dimensions along the diagonals of the rhombic opening include the van-der-Waals radii of the atoms (the grid unit is 1 Å).

Section S3.3: Additional structure description of MOF-5 (**Figure S15**)

MOF-5 (also named IRMOF-1), $[Zn_4O(bdc)_3]$ with bdc = benzene-1,4-dicarboxylate, terephthalate) is one of the best known and prototypical MOFs [19]. The structure of MOF-5 is constructed of tetrahedral $\{Zn_4O\}$ secondary building units where six carboxylate groups of the terephthalate linkers span the six edges of the $\{Zn_4O\}$ tetrahedron in an octahedral fashion to give a 3D primitive cubic (**pcu**) structure with orthogonal channels along each axis of a cartesian coordinate system with channel cross-sections (window size) of 8 × 8 Å and a pore diameter of 15 Å (Figure S15) [8]. Activated MOF-5 has a high surface area (3000 m^2/g) and high thermal stability (up to 400 °C) [19].

(**a**)

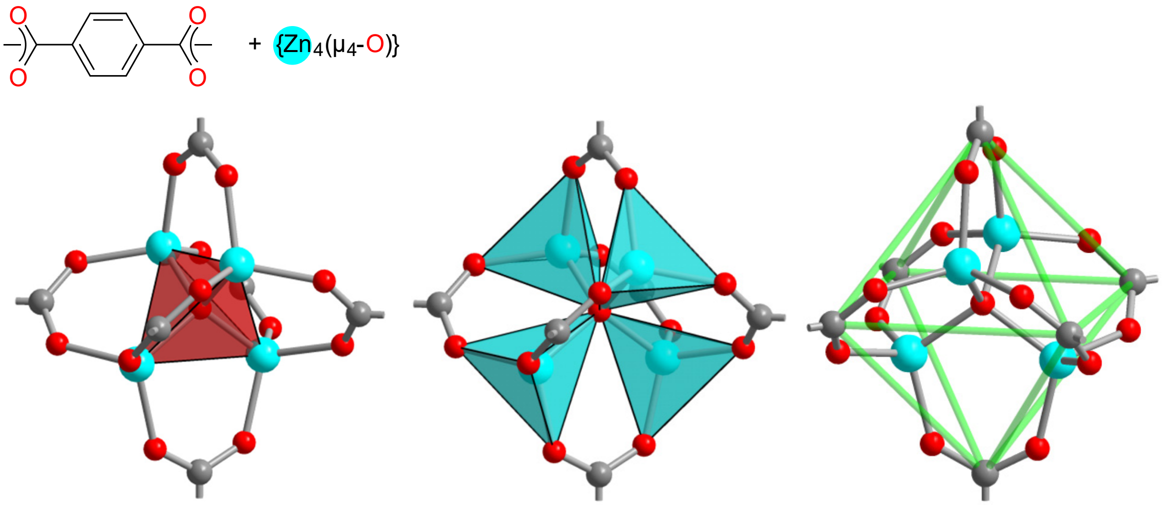

(**b**)

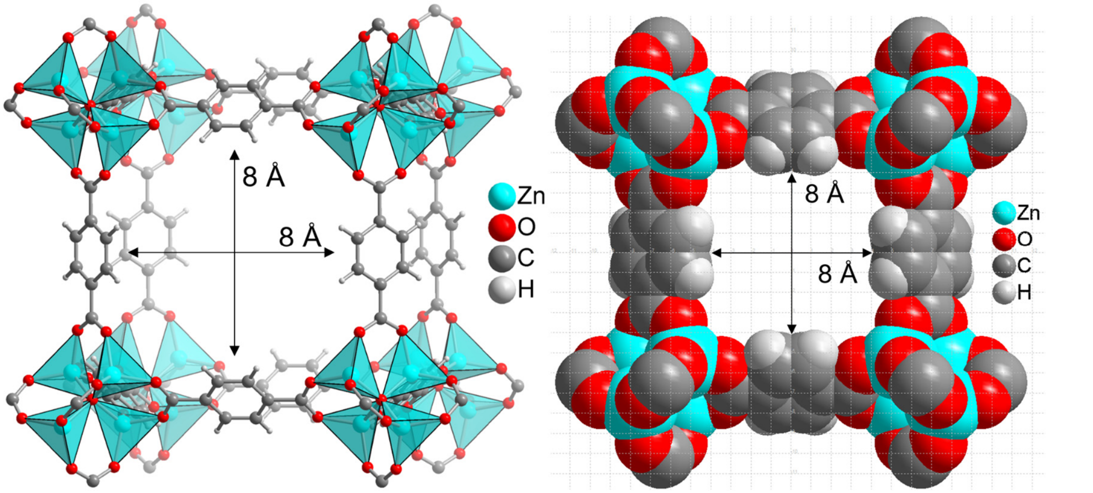

(**c**)

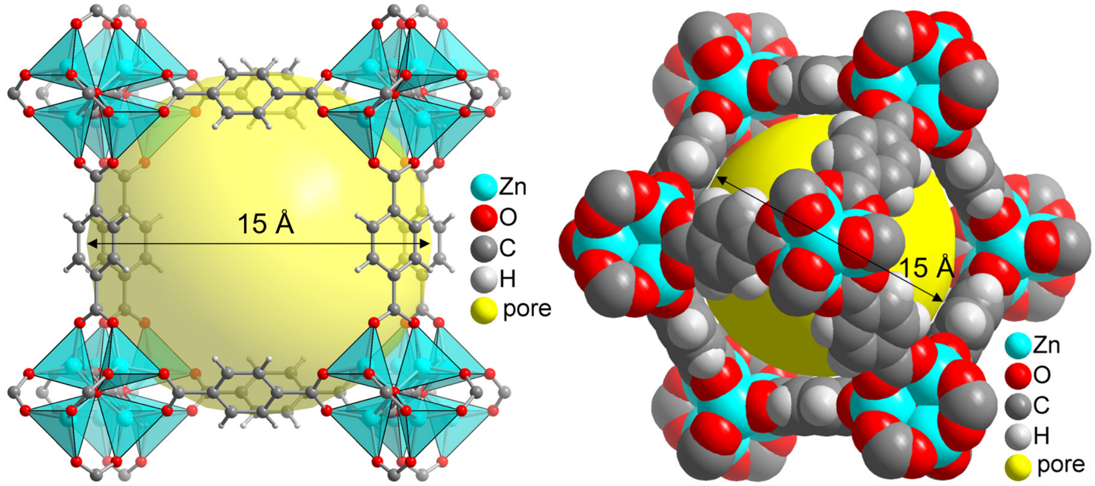

(**d**)

Figure S15. (**a**) Benzene-1,4-dicarboxylate, terephthalate, bdc and μ_4-oxido bridged tetranuclear zinc cluster as building blocks for MOF-5 (IRMOF-1), 3D-[$Zn_4O(bdc)_3$].

(**b**) {Zn_4O} SBU with six carboxylate groups in MOF-5, emphasizing the tetrahedral environment of the central μ-oxido atom, the tetrahedral Zn coordination and the carboxylate carbon atom positions at the vertices of an octahedron.

(**c**) Section of the MOF-5 **pcu** framework in ball-and-stick and space-filling mode viewed along one of channel directions. The edge-to-edge dimensions of the square opening (cross-section) of 8 × 8 Å extend to the van-der-Waals radii of the atoms (the grid unit is 1 Å). (**d**) The pore diameter of 15 Å extends to the van-der-Waals surface of the framework atoms. At right, view along the cube diagonal. The objects in (**b**) to (**d**) are not drawn to scale. (Structure images were drawn with Diamond [9] from the deposited cif file under CCDC-no./Refcode 256966/SAHYOQ [19,20].)

Section S4: Nitrogen adsorption isotherms and powder X-ray diffractograms (Figure S16 - Figure S19)

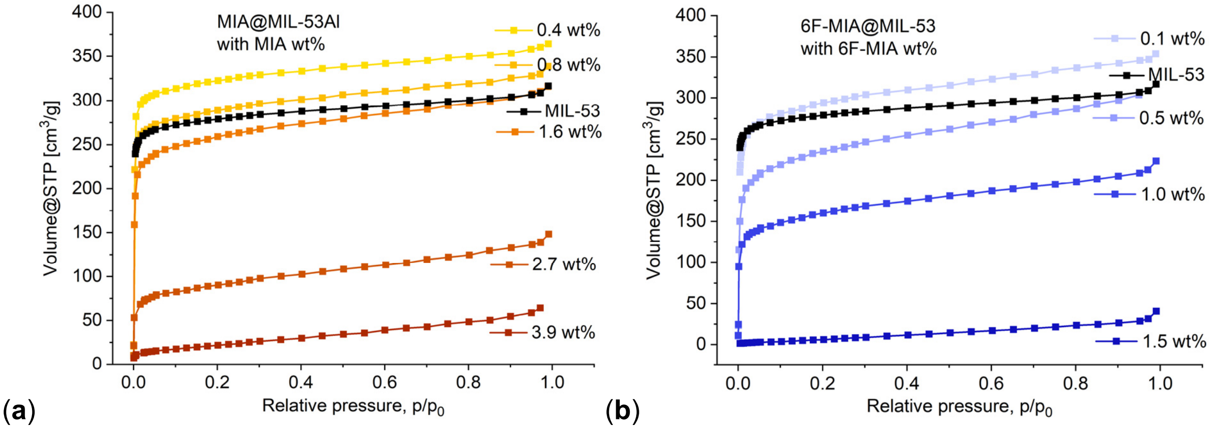

Figure S16. Nitrogen adsorption isotherms (77 K) of (**a**) MIA@MIL-53 and (**b**) 6F-MIA@MIL-53 with different flavin amounts.

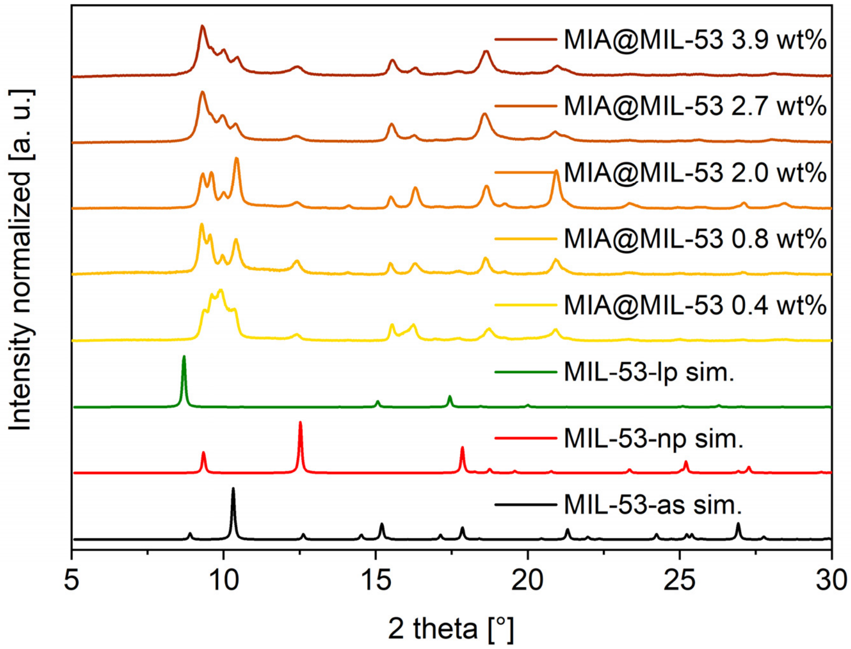

(**a**)

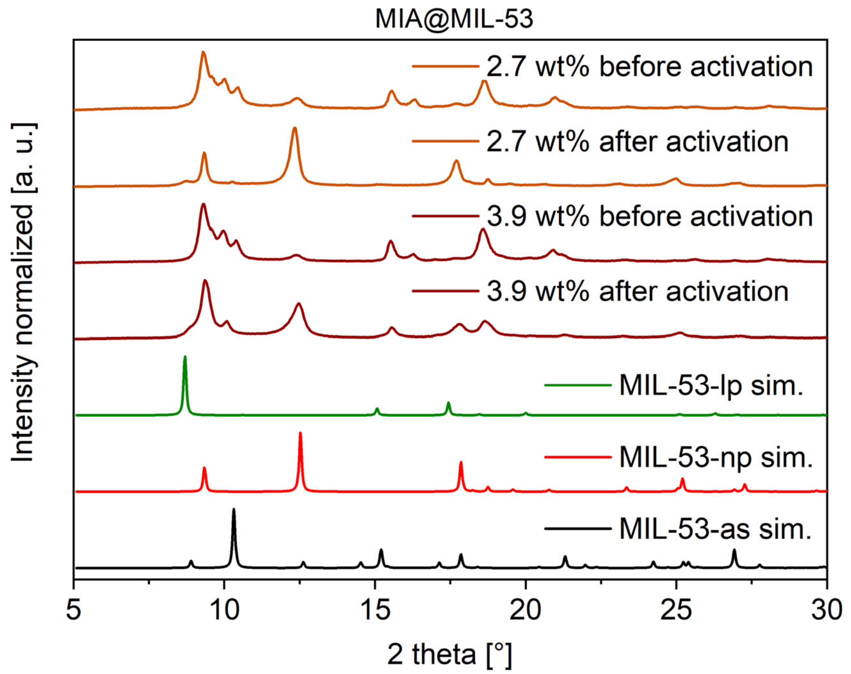

(**b**)

Figure S17. Comparison of the PXRD patterns of MIA@MIL-53 with varying flavin amounts (**a**) *before* activation and (**b**) *before and after* activation for 2.7 wt% and 3.9 wt% with the simulated PXRDs for MIL-53-as, -np and -lp based on the deposited cif files under CCDC-no./Refcode 220475/SABVOH for MIL-53-as, 220476/SABVUN for MIL-53-lp and 220477/ SABWAU for MIL-53-np [6].

The slight shift in the corresponding peak positions between the experimental and simulated diffractograms in Figure S17 is due to different cell constants from the formation of the experimental MIA@MIL-53 phases which only approximate the published MIL-53-as, -lp and -np phases. The simulated patterns from these published MIL-53-as, -lp and -np phases are based on Rietveld

refinements of PXRDs from a specific sample treatment protocol [6], which understandably differs from our treatment of the composite samples. An X-ray thermodiffractogram of MIL-53-as in ref. [6] illustrates the peak shift in the diffractograms with temperature. Hence, the experimental diffractograms of flavin@MIL-53 reflect 'breathing' phases which can only approximate the literature phases obtained at different conditions. Yet, the peak pattern allows for an unequivocal phase assignment.

The transition from as-synthesized to large-pore form required high temperature (cf. Figure S13), due to the removal of residual terephthalic acid. When the terephthalic acid is already removed through the washing steps concomitant with the flavin insertion, then there is only volatile solvent remaining in the as-synthesized form. Subsequently, the transition from as-synthesized to large-pore and further to narrow-pore form can occur upon sample storage and preparation under ambient air conditions. The reflections indicate a phase mixture that additionally varies with the different amounts of flavin incorporated.

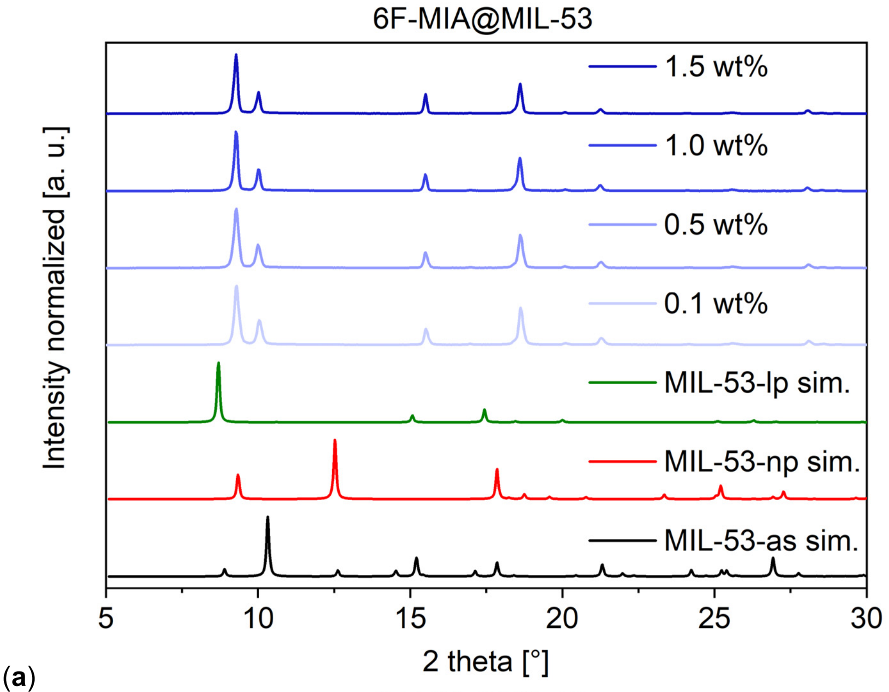

(**a**)

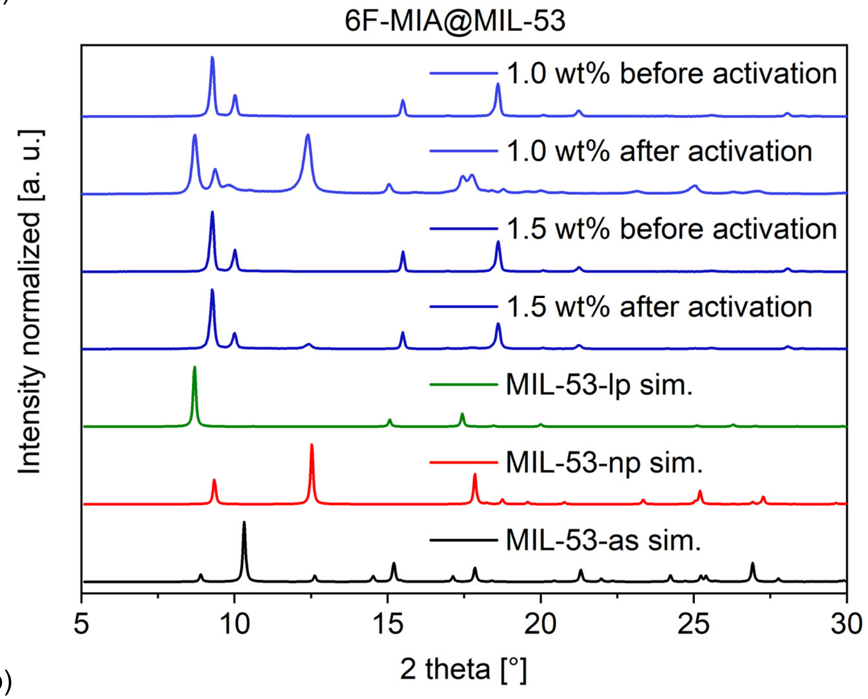

(**b**)

Figure S18. Comparison of the PXRD patterns of 6F-MIA@MIL-53 with varying flavin amounts (**a**) *before* activation and (**b**) *before and after* activation for 1.0 wt% and 1.5 wt%. The simulated PXRDs are based on the deposited cif files under CCDC-no./Refcode 220475/SABVOH for MIL-53-as, 220476/SABVUN for MIL-53-lp and 220477/ SABWAU for MIL-53-np [6].

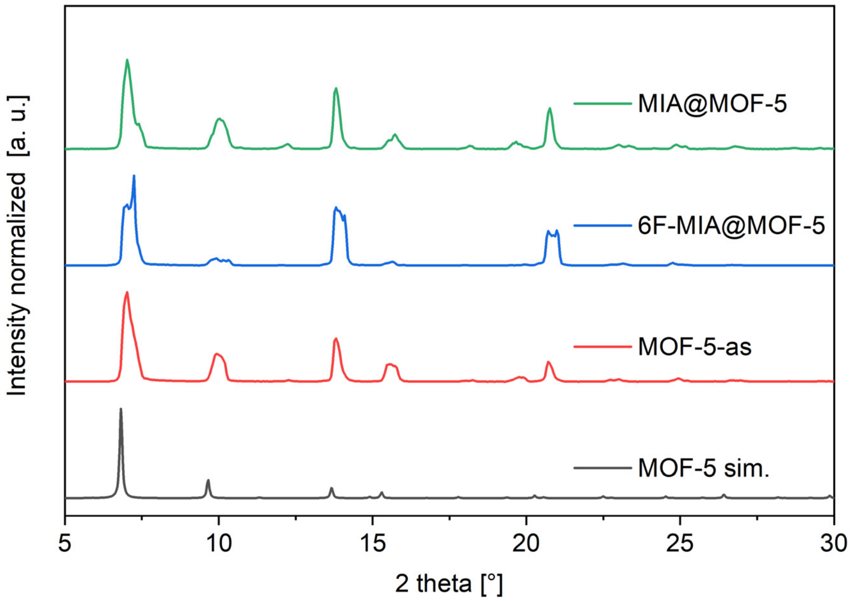

(**a**)

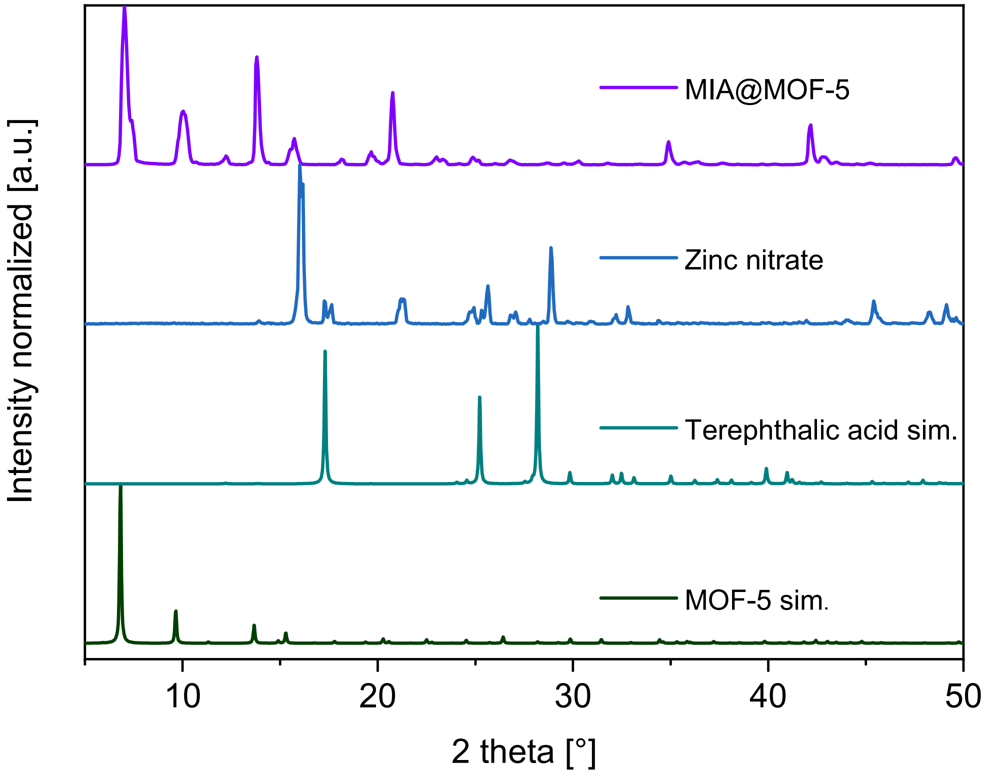

(**b**)

Figure S19. Comparison of the PXRD patterns of (**a**) MIA@MOF-5 and 6F-MIA@MOF-5 with simulated MOF-5; (**b**) MIA@MOF-5 with the measured patterns of zinc nitrate hexahydrate and simulated terephthalic acid and MOF-5. (Note the different 2theta range in (a) and (b). The simulated PXRDs are based on the deposited cif files under CCDC-no./Refcode 1269122/TEPHTH for terephthalic acid [21] and CCDC-no./Refcode 256966/SAHYOQ for MOF-5 [20,22].

Section S5: Photophysical characterization of the dyes and dye@MOF (Figure S20 - Figure S35)

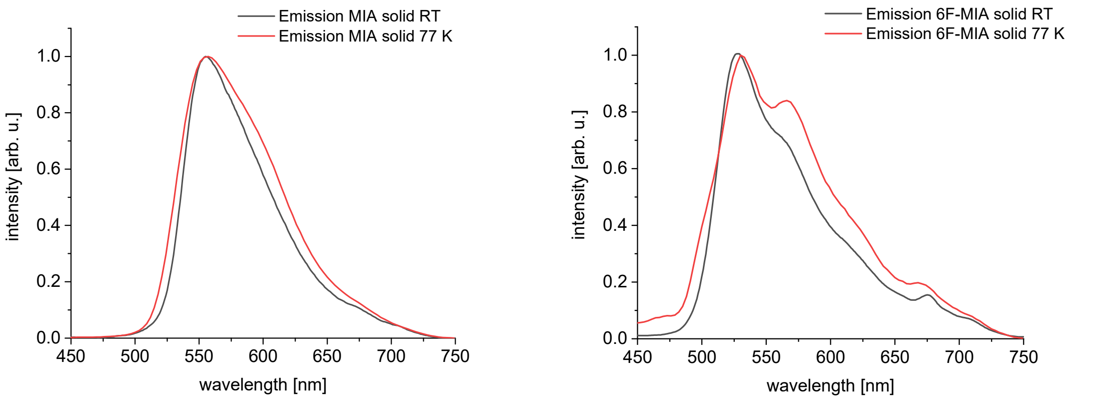

Figure S20. Photoluminescence spectra of MIA (left) and 6F-MIA (right) (λ_{exc} = 440 nm) at room temperature, RT, (black) and at 77 K (red).

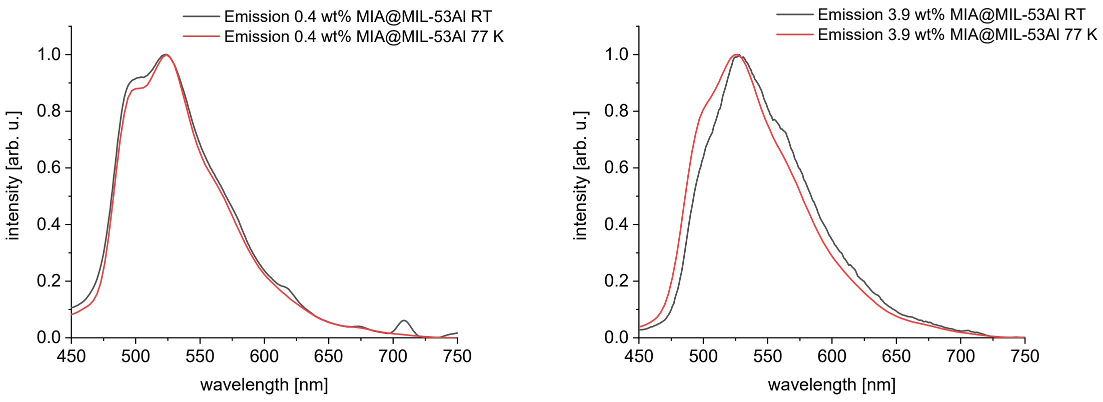

Figure S21. Photoluminescence spectra of MIA@MIL53Al with 0.4 wt % loading (left) and 3.9 wt % loading (right) (λ_{exc} = 440 nm) at room temperature, RT, (black) and at 77 K (red).

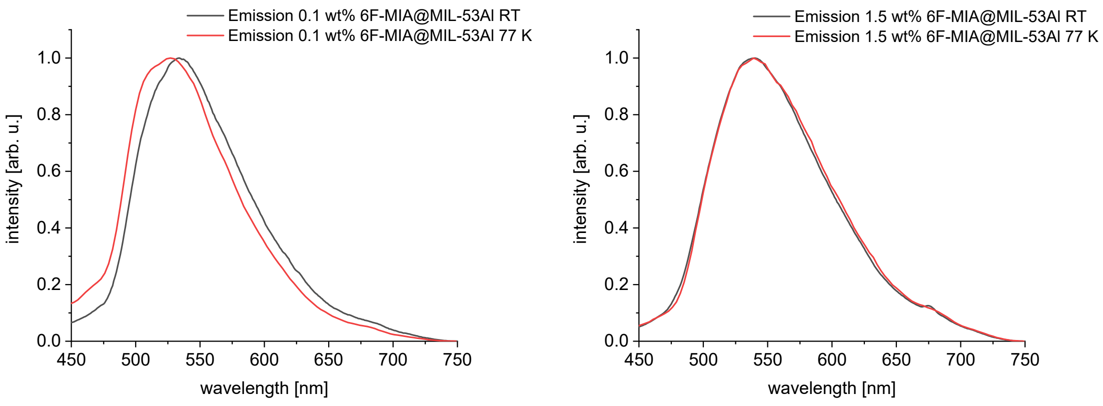

Figure S22. Photoluminescence spectra of 6F-MIA@MIL53Al with 0.1 wt % loading (left) and 1.5 wt % loading (right) (λ_{exc} = 440 nm) at room temperature, RT, (black) and at 77 K (red).

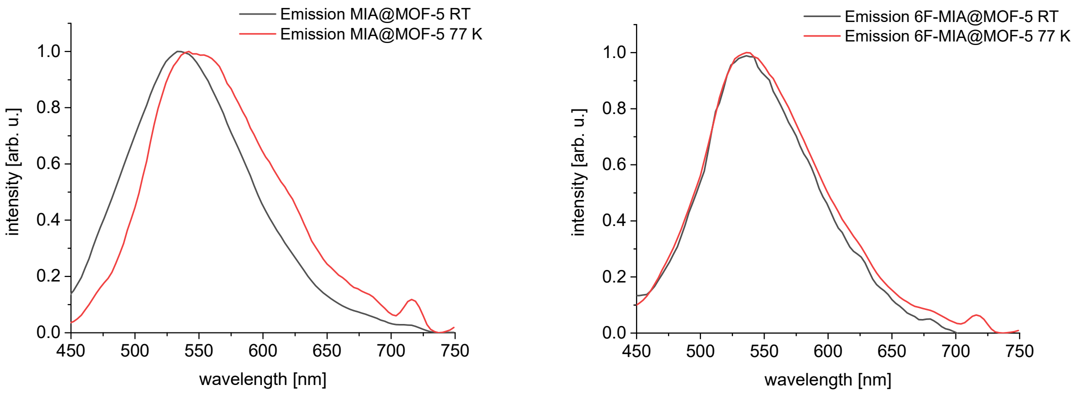

Figure S23. Photoluminescence spectra of MIA@MOF-5 (left) and 6F-MIA@MOF-5 (right) (λ_{exc} = 440 nm) at room temperature, RT, (black) and at 77 K (red).

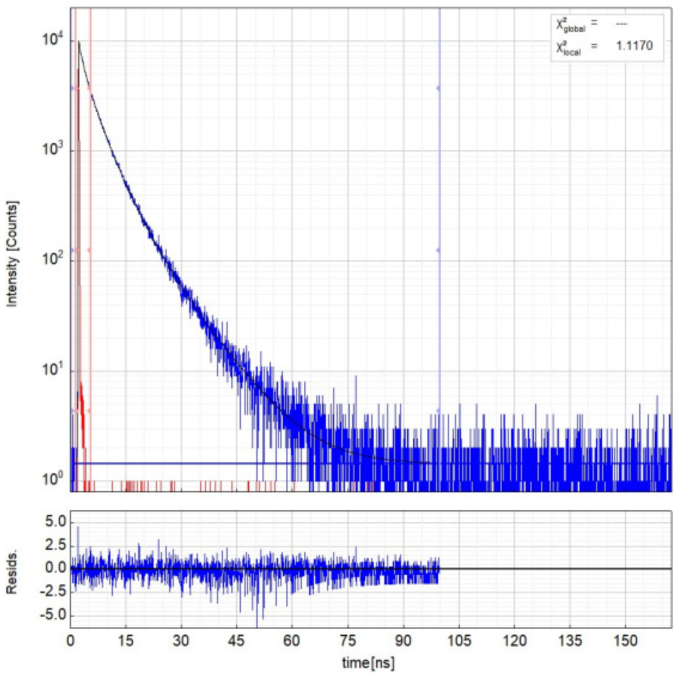

Parameter	Value	Error
A_1[kCnts/Chnl]	0.95	± 0.30
τ_1[ns]	9.47	± 0.75
I_1[kCnts]	141	± 33
$A_{Rel\,1}$[%]	9.5	± 2.8
$I_{Rel\,1}$[%]	24.6	± 5.6
A_2[kCnts/Chnl]	5.53	± 0.31
τ_2[ns]	4.19	± 0.35
I_2[kCnts]	362	± 18
$A_{Rel\,2}$[%]	55.1	± 2.4
$I_{Rel\,2}$[%]	63.0	± 3.1
A_3[kCnts/Chnl]	3.55	± 0.45
τ_3[ns]	1.29	± 0.20
I_3[kCnts]	71	± 20
$A_{Rel\,3}$[%]	35.4	± 4.9
$I_{Rel\,3}$[%]	12.4	± 3.4
$Bkgr_{Dec}$[kCnts]	0.0014	± 0.0002
$Bkgr_{IRF}$[Cnts/Chnl]	0.19	± 0.55
$Shift_{IRF}$[ps]	-47	± 18
A_{Scatt}[kCnts]	8.0	± 4.6
$\tau_{Av\,Int}$[ns]	5.124	± 0.031
$\tau_{Av\,Amp}$[ns]	3.662	± 0.062

Figure S24. Left: Raw time-resolved photoluminescence decay (blue) of MIA@MOF-5 in the solid state at 298 K and the instrument response function (red), including the residuals (λ_{ex} = 405 nm, λ_{em} = 530 nm). Right: Fitting parameters including pre-exponential factors and confidence limits. Here, τ_{av_int} is the intensity-weighted average lifetime (τ_F) and τ_{av_amp} is amplitude-weighted average lifetime (τ_x).

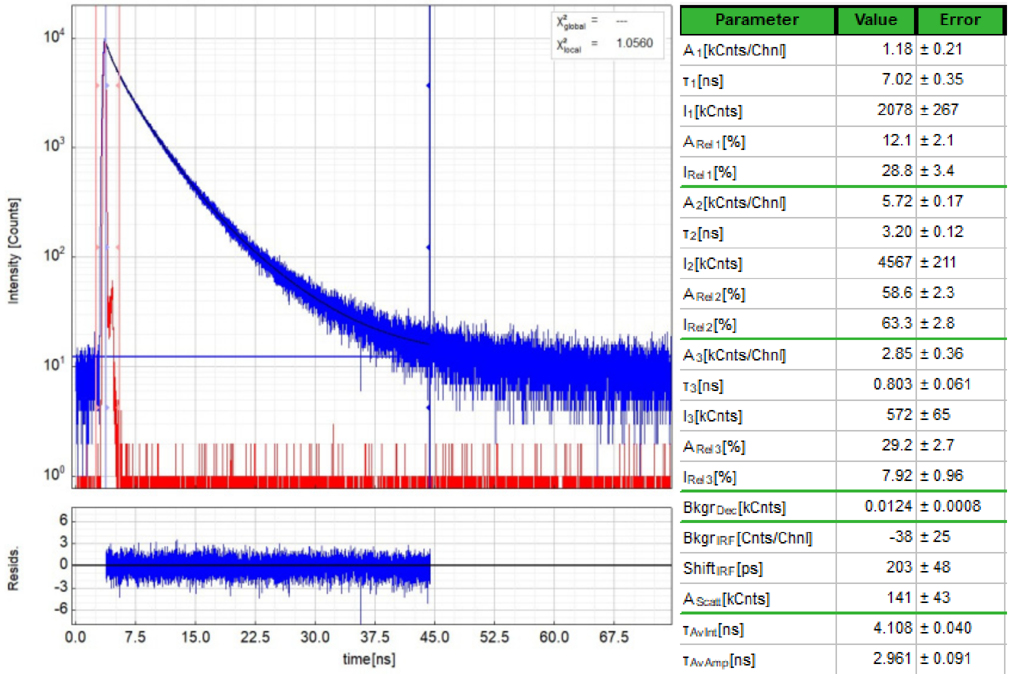

Parameter	Value	Error
A_1[kCnts/Chnl]	1.18	± 0.21
τ_1[ns]	7.02	± 0.35
I_1[kCnts]	2078	± 267
$A_{Rel\,1}$[%]	12.1	± 2.1
$I_{Rel\,1}$[%]	28.8	± 3.4
A_2[kCnts/Chnl]	5.72	± 0.17
τ_2[ns]	3.20	± 0.12
I_2[kCnts]	4567	± 211
$A_{Rel\,2}$[%]	58.6	± 2.3
$I_{Rel\,2}$[%]	63.3	± 2.8
A_3[kCnts/Chnl]	2.85	± 0.36
τ_3[ns]	0.803	± 0.061
I_3[kCnts]	572	± 65
$A_{Rel\,3}$[%]	29.2	± 2.7
$I_{Rel\,3}$[%]	7.92	± 0.96
$Bkgr_{Dec}$[kCnts]	0.0124	± 0.0008
$Bkgr_{IRF}$[Cnts/Chnl]	-38	± 25
$Shift_{IRF}$[ps]	203	± 48
A_{Scatt}[kCnts]	141	± 43
$\tau_{Av\,Int}$[ns]	4.108	± 0.040
$\tau_{Av\,Amp}$[ns]	2.961	± 0.091

Figure S25. Left: Raw time-resolved photoluminescence decay (blue) of 6F-MIA@MOF-5 in the solid state at 298 K and the instrument response function (red), including the residuals (λ_{ex} = 405 nm, λ_{em} = 510 nm). Right: Fitting parameters including pre-exponential factors and confidence limits. Here, τ_{av_int} is the intensity-weighted average lifetime (τ_F) and τ_{av_amp} is amplitude-weighted average lifetime (τ_x).

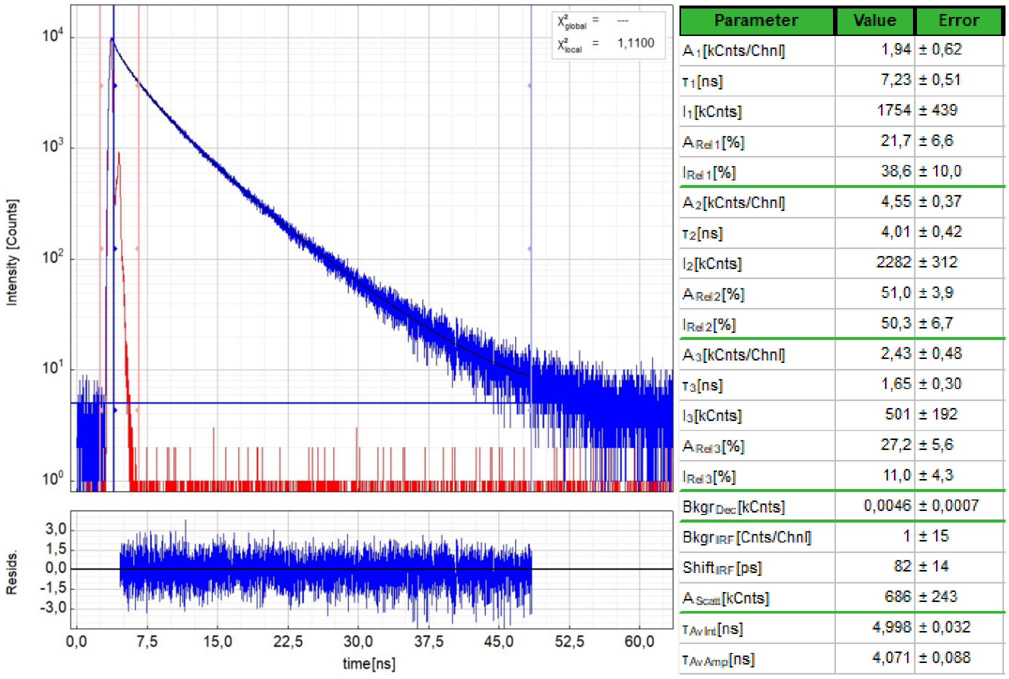

Parameter	Value	Error
A_1[kCnts/Chnl]	1,94	± 0,62
τ_1[ns]	7,23	± 0,51
I_1[kCnts]	1754	± 439
$A_{Rel\,1}$[%]	21,7	± 6,6
$I_{Rel\,1}$[%]	38,6	± 10,0
A_2[kCnts/Chnl]	4,55	± 0,37
τ_2[ns]	4,01	± 0,42
I_2[kCnts]	2282	± 312
$A_{Rel\,2}$[%]	51,0	± 3,9
$I_{Rel\,2}$[%]	50,3	± 6,7
A_3[kCnts/Chnl]	2,43	± 0,48
τ_3[ns]	1,65	± 0,30
I_3[kCnts]	501	± 192
$A_{Rel\,3}$[%]	27,2	± 5,6
$I_{Rel\,3}$[%]	11,0	± 4,3
$Bkgr_{Dec}$[kCnts]	0,0046	± 0,0007
$Bkgr_{IRF}$[Cnts/Chnl]	1	± 15
$Shift_{IRF}$[ps]	82	± 14
A_{Scat}[kCnts]	686	± 243
$\tau_{Av\,Int}$[ns]	4,998	± 0,032
$\tau_{Av\,Amp}$[ns]	4,071	± 0,088

Figure S26. Left: Raw time-resolved photoluminescence decay (blue) of 0.4 wt% MIA@MIL-53 in the solid state at 298 K and the instrument response function (red), including the residuals (λ_{ex} = 405 nm, λ_{em} = 520 nm). Right: Fitting parameters including pre-exponential factors and confidence limits. Here, τ_{av_int} is the intensity-weighted average lifetime (τ_F) and τ_{av_amp} is amplitude-weighted average lifetime (τ_x).

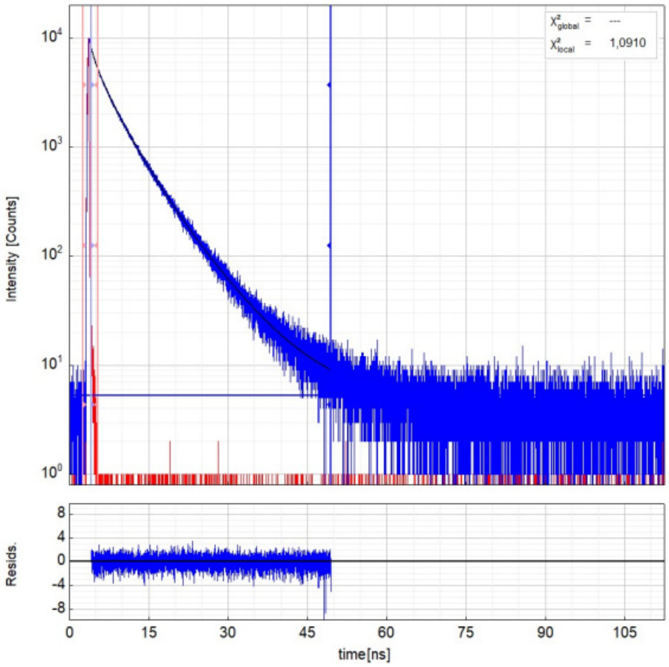

Parameter	Value	Error	Plot
A_1[kCnts/Chnl]	1,65	± 0,55	☐
τ_1[ns]	7,55	± 0,55	☑
I_1[kCnts]	1562	± 400	☐
$A_{Rel\,1}$[%]	17,0	± 5,3	☐
$I_{Rel\,1}$[%]	34,3	± 8,7	☐
A_2[kCnts/Chnl]	4,93	± 0,20	☐
τ_2[ns]	4,02	± 0,36	☑
I_2[kCnts]	2479	± 307	☐
$A_{Rel\,2}$[%]	50,6	± 3,0	☐
$I_{Rel\,2}$[%]	54,4	± 6,8	☐
A_3[kCnts/Chnl]	3,16	± 0,22	☐
τ_3[ns]	1,31	± 0,17	☐
I_3[kCnts]	518	± 93	☐
$A_{Rel\,3}$[%]	32,4	± 2,6	☐
$I_{Rel\,3}$[%]	11,4	± 2,1	☐
$Bkgr_{Dec}$[kCnts]	0,0053	± 0,0008	☐
$Bkgr_{IRF}$[Cnts/Chnl]	0,6	± 1,6	☐
$Shift_{IRF}$[ps]	-28,2	± 1,2	☐
A_{Scat}[kCnts]	5791	± 1907	☐
$\tau_{Av\,Int}$[ns]	4,924	± 0,042	☐
$\tau_{Av\,Amp}$[ns]	3,745	± 0,091	☐

Figure S27. Left: Raw time-resolved photoluminescence decay (blue) of 3.9 wt% MIA@MIL-53 in the solid state at 298 K and the instrument response function (red), including the residuals (λ_{ex} = 405 nm, λ_{em} = 525 nm). Right: Fitting parameters including pre-exponential factors and confidence limits. Here, τ_{av_int} is the intensity-weighted average lifetime (τ_F) and τ_{av_amp} is amplitude-weighted average lifetime (τ_x).

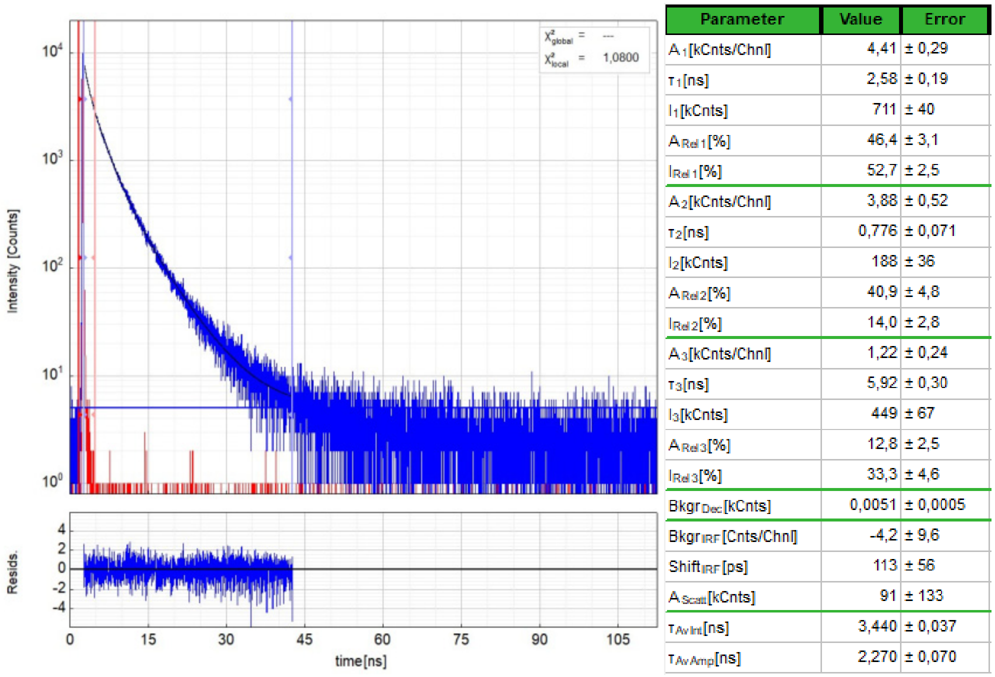

Parameter	Value	Error
A_1[kCnts/Chnl]	4,41	± 0,29
τ_1[ns]	2,58	± 0,19
I_1[kCnts]	711	± 40
$A_{Rel\,1}$[%]	46,4	± 3,1
$I_{Rel\,1}$[%]	52,7	± 2,5
A_2[kCnts/Chnl]	3,88	± 0,52
τ_2[ns]	0,776	± 0,071
I_2[kCnts]	188	± 36
$A_{Rel\,2}$[%]	40,9	± 4,8
$I_{Rel\,2}$[%]	14,0	± 2,8
A_3[kCnts/Chnl]	1,22	± 0,24
τ_3[ns]	5,92	± 0,30
I_3[kCnts]	449	± 67
$A_{Rel\,3}$[%]	12,8	± 2,5
$I_{Rel\,3}$[%]	33,3	± 4,6
$Bkgr_{Dec}$[kCnts]	0,0051	± 0,0005
$Bkgr_{IRF}$[Cnts/Chnl]	-4,2	± 9,6
$Shift_{IRF}$[ps]	113	± 56
A_{Scatt}[kCnts]	91	± 133
$\tau_{Av\,Int}$[ns]	3,440	± 0,037
$\tau_{Av\,Amp}$[ns]	2,270	± 0,070

Figure S28. Left: Raw time-resolved photoluminescence decay (blue) of 0.1 wt% 6F-MIA@MIL-53 in the solid state at 298 K and the instrument response function (red), including the residuals (λ_{ex} = 405 nm, λ_{em} = 535 nm). Right: Fitting parameters including pre-exponential factors and confidence limits. Here, τ_{av_int} is the intensity-weighted average lifetime (τ_F) and τ_{av_amp} is amplitude-weighted average lifetime (τ_x).

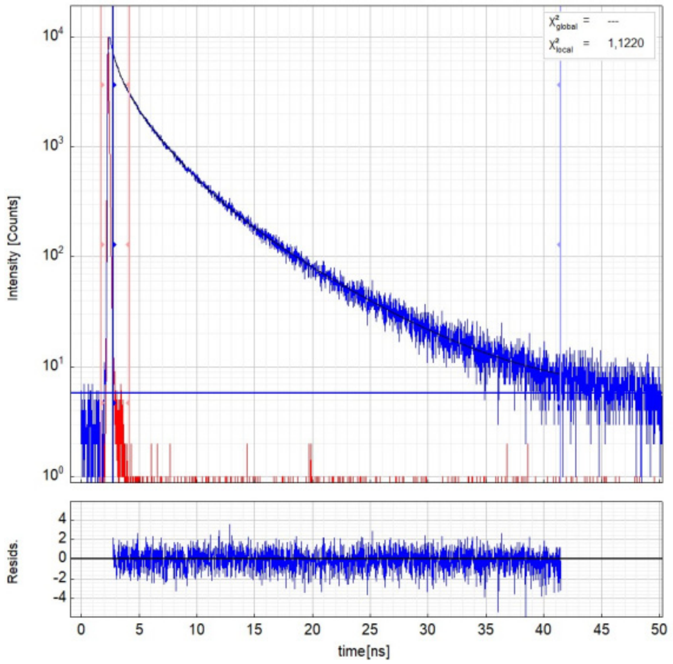

Parameter	Value	Error
A_1[kCnts/Chnl]	0,944	± 0,089
τ_1[ns]	6,70	± 0,21
I_1[kCnts]	395	± 26
$A_{Rel\,1}$[%]	9,11	± 0,93
$I_{Rel\,1}$[%]	32,5	± 2,0
A_2[kCnts/Chnl]	3,73	± 0,14
τ_2[ns]	2,547	± 0,095
I_2[kCnts]	594	± 21
$A_{Rel\,2}$[%]	36,0	± 1,6
$I_{Rel\,2}$[%]	48,9	± 1,3
A_3[kCnts/Chnl]	5,69	± 0,39
τ_3[ns]	0,633	± 0,040
I_3[kCnts]	225	± 19
$A_{Rel\,3}$[%]	54,9	± 2,3
$I_{Rel\,3}$[%]	18,6	± 1,4
$Bkgr_{Dec}$[kCnts]	0,0057	± 0,0005
$Bkgr_{IRF}$[Cnts/Chnl]	-27,8	± 9,5
$Shift_{IRF}$[ps]	-25	± 29
A_{Scatt}[kCnts]	-38	± 84
$\tau_{Av\,Int}$[ns]	3,542	± 0,038
$\tau_{Av\,Amp}$[ns]	1,874	± 0,066

Figure S29. Left: Raw time-resolved photoluminescence decay (blue) of 1.5 wt% 6F-MIA@MIL-53 in the solid state at 298 K and the instrument response function (red), including the residuals (λ_{ex} = 405 nm, λ_{em} = 535 nm). Right: Fitting parameters including pre-exponential factors and confidence limits. Here, τ_{av_int} is the intensity-weighted average lifetime (τ_F) and τ_{av_amp} is amplitude-weighted average lifetime (τ_x).

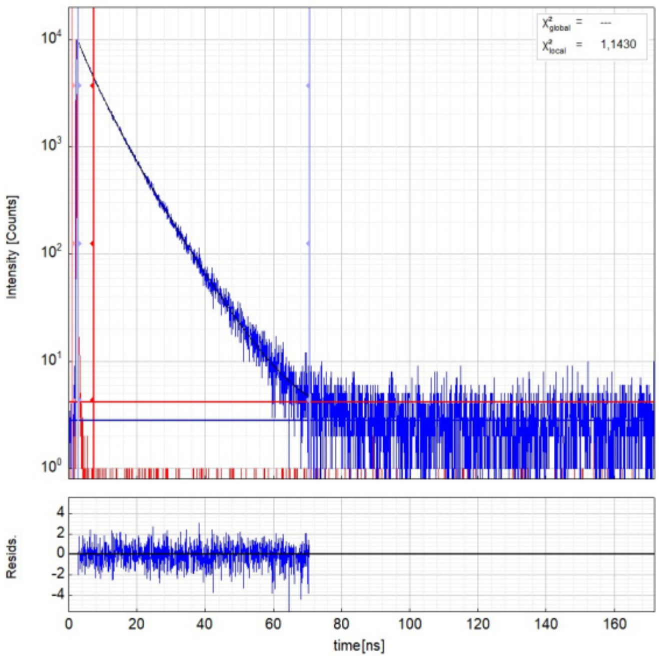

Parameter	Value	Error
A_1[kCnts/Chnl]	1,99	± 0,30
τ_1[ns]	2,87	± 0,28
I_1[kCnts]	89	± 19
$A_{Rel\,1}$[%]	18,5	± 2,6
$I_{Rel\,1}$[%]	8,4	± 1,7
A_2[kCnts/Chnl]	2,13	± 0,11
τ_2[ns]	10,15	± 0,13
I_2[kCnts]	338	± 16
$A_{Rel\,2}$[%]	19,84	± 0,94
$I_{Rel\,2}$[%]	31,8	± 1,5
A_3[kCnts/Chnl]	6,61	± 0,31
τ_3[ns]	6,14	± 0,11
I_3[kCnts]	634	± 28
$A_{Rel\,3}$[%]	61,6	± 3,0
$I_{Rel\,3}$[%]	59,8	± 2,5
$Bkgr_{Dec}$[kCnts]	0,0017	± 0,0005
$Bkgr_{IRF}$[Cnts/Chnl]	0,6	± 2,1
$Shift_{IRF}$[ps]	-65	± 59
A_{Scatt}[kCnts]	212	± 204
$\tau_{Av\,Int}$[ns]	7,139	± 0,027
$\tau_{Av\,Amp}$[ns]	6,329	± 0,067

Figure S30. Left: Raw time-resolved photoluminescence decay (blue) of MIA in a DCM/MeOH 1:1 fluid solution at 298 K and the instrument response function (red), including the residuals (λ_{ex} = 405 nm, λ_{em} = 525 nm). Right: Fitting parameters including pre-exponential factors and confidence limits. Here, τ_{av_int} is the intensity-weighted average lifetime (τ_F) and τ_{av_amp} is amplitude-weighted average lifetime (τ_x).

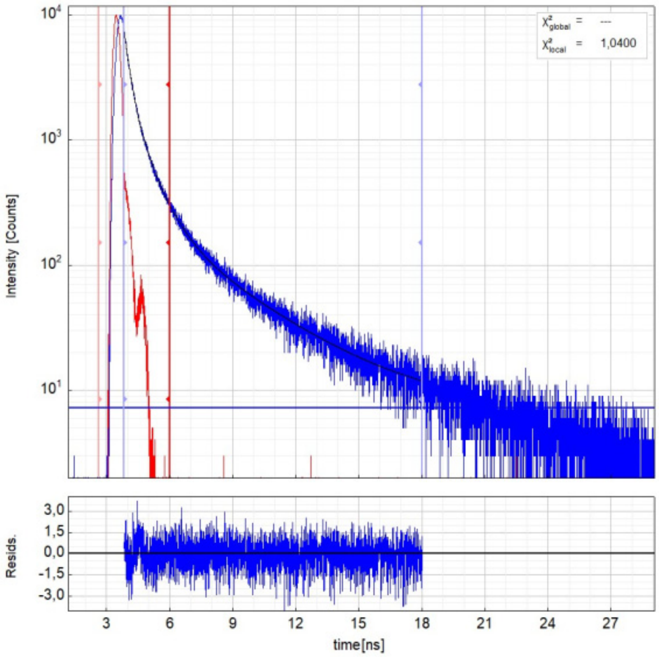

Parameter	Value	Error
A_1[kCnts/Chnl]	0,305	± 0,055
τ_1[ns]	3,48	± 0,37
I_1[kCnts]	265	± 20
$A_{Rel\,1}$[%]	1,20	± 0,18
$I_{Rel\,1}$[%]	12,4	± 1,1
A_2[kCnts/Chnl]	2,97	± 0,54
τ_2[ns]	0,819	± 0,093
I_2[kCnts]	608	± 35
$A_{Rel\,2}$[%]	11,7	± 1,6
$I_{Rel\,2}$[%]	28,4	± 1,8
A_3[kCnts/Chnl]	22,2	± 1,8
τ_3[ns]	0,228	± 0,013
I_3[kCnts]	1266	± 98
$A_{Rel\,3}$[%]	87,1	± 1,8
$I_{Rel\,3}$[%]	59,2	± 2,7
$Bkgr_{Dec}$[kCnts]	0,0072	± 0,0017
$Bkgr_{IRF}$[Cnts/Chnl]	1,9	± 9,0
$Shift_{IRF}$[ps]	-29	± 17
A_{Scatt}[kCnts]	-344	± 322
$\tau_{Av\,Int}$[ns]	0,798	± 0,056
$\tau_{Av\,Amp}$[ns]	0,336	± 0,015

Figure S31. Left: Raw time-resolved photoluminescence decay (blue) of MIA in the solid state at 298 K and the instrument response function (red), including the residuals (λ_{ex} = 405 nm, λ_{em} = 550 nm). Right: Fitting parameters including pre-exponential factors and confidence limits. Here, τ_{av_int} is the intensity-weighted average lifetime (τ_F) and τ_{av_amp} is amplitude-weighted average lifetime (τ_x).

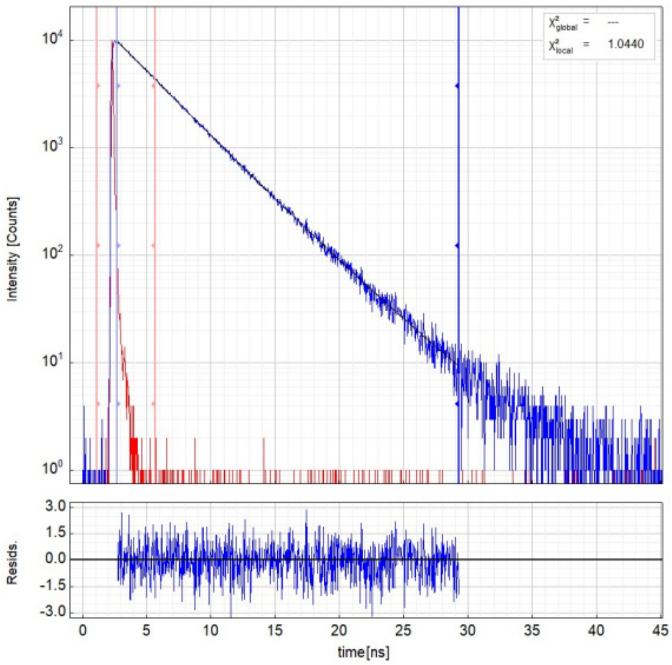

Parameter	Value	Error
A_1[kCnts/Chnl]	1.895	± 0.096
τ_1[ns]	4.62	± 0.11
I_1[kCnts]	274	± 15
$A_{Rel\,1}$[%]	17.30	± 0.81
$I_{Rel\,1}$[%]	22.4	± 1.1
A_2[kCnts/Chnl]	9.06	± 0.16
τ_2[ns]	3.357	± 0.043
I_2[kCnts]	950	± 21
$A_{Rel\,2}$[%]	82.70	± 0.81
$I_{Rel\,2}$[%]	77.6	± 1.1
$Bkgr_{Dec}$[kCnts]	0.0007	± 0.0009
$Bkgr_{IRF}$[Cnts/Chnl]	-5.8	± 3.1
$Shift_{IRF}$[ps]	-3	± 41
A_{Scatt}[kCnts]	57	± 116
$\tau_{Av\,Int}$[ns]	3.6401	± 0.0083
$\tau_{Av\,Amp}$[ns]	3.576	± 0.018

Figure S32. Left: Raw time-resolved photoluminescence decay (blue) of 6F-MIA in a DCM/MeOH 1:1 fluid solution at 298 K and the instrument response function (red), including the residuals (λ_{ex} = 405 nm, λ_{em} = 525 nm). Right: Fitting parameters including pre-exponential factors and confidence limits. Here, τ_{av_int} is the intensity-weighted average lifetime (τ_F) and τ_{av_amp} is amplitude-weighted average lifetime (τ_x).

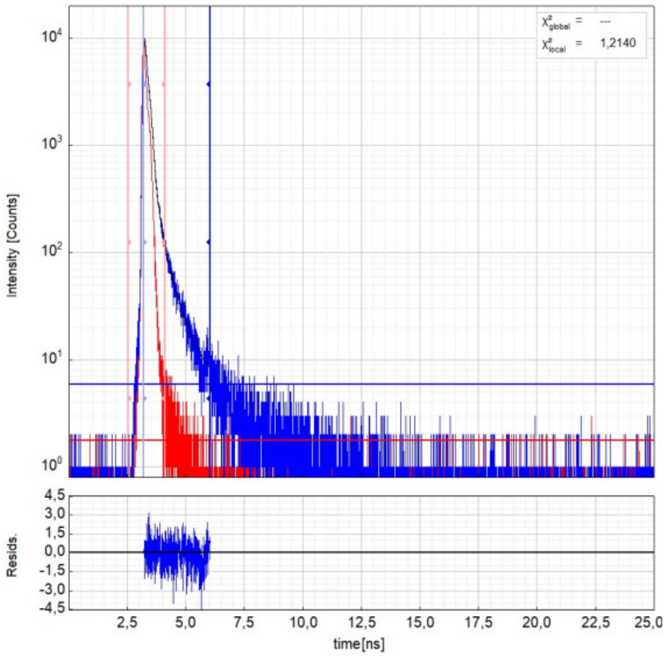

Parameter	Value	Error
A_1[kCnts/Chnl]	1,72	± 0,28
τ_1[ns]	0,186	± 0,021
I_1[kCnts]	80,2	± 7,6
$A_{Rel\,1}$[%]	4,57	± 0,62
$I_{Rel\,1}$[%]	14,6	± 1,2
A_2[kCnts/Chnl]	0,412	± 0,070
τ_2[ns]	0,541	± 0,035
I_2[kCnts]	55,8	± 5,7
$A_{Rel\,2}$[%]	1,09	± 0,20
$I_{Rel\,2}$[%]	10,2	± 1,2
A_3[kCnts/Chnl]	35,6	± 3,7
τ_3[ns]	0,0464	± 0,0022
I_3[kCnts]	413	± 29
$A_{Rel\,3}$[%]	94,33	± 0,74
$I_{Rel\,3}$[%]	75,2	± 1,6
$Bkgr_{Dec}$[kCnts]	0,0059	± 0,0008
$Bkgr_{IRF}$[Cnts/Chnl]	1,8	± 3,2
$Shift_{IRF}$[ps]	-5,3	± 1,9
A_{Scatt}[kCnts]	116	± 32
$\tau_{Av\,Int}$[ns]	0,1172	± 0,0049
$\tau_{Av\,Amp}$[ns]	0,0582	± 0,0028

Figure S33. Left: Raw time-resolved photoluminescence decay (blue) of 6F-MIA in the solid state at 298 K and the instrument response function (red), including the residuals (λ_{ex} = 405 nm, λ_{em} = 550 nm). Right: Fitting parameters including pre-exponential factors and confidence limits. Here, τ_{av_int} is the intensity-weighted average lifetime (τ_F) and τ_{av_amp} is amplitude-weighted average lifetime (τ_x).

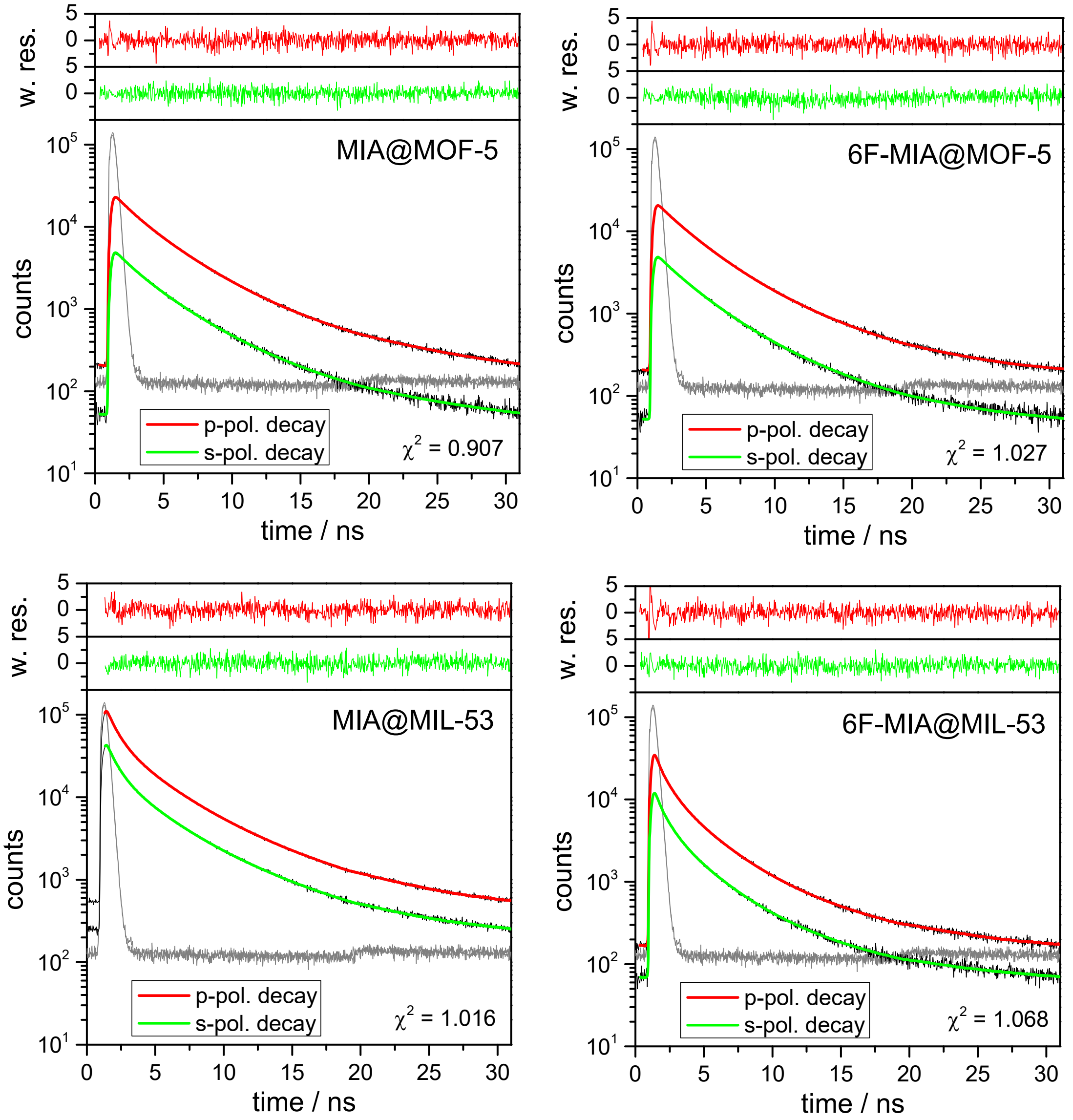

Figure S34. Time-correlated polarization-resolved single-photon data of the flavin@MOF composites imaged by confocal multi-parameter fluorescence image spectroscopy (MFIS) with pulsed excitation at 440 nm and λ_{em} = 502-538 nm (Figure 6, main document). Global fits by iterative reconvolution of the instrumental function (grey curves) to p- and s-polarized decays required 4 fluorescence lifetime components (lifetimes and species fractions are compiled in Table 3 of the main document) and one (two for MIA@MIL-53) rotational correlation time. The corresponding equations are described in ref. [23]. The offsets in the decays are caused by afterpulsing of the detectors and taken into account by the fitting routine. The *G*-factor, compensating differences in the detection efficiencies in the two polarization channels, of *G* = 0.97 was determined by fitting polarized fluorescence decays of an aqueous solution of Rhodamine 110. For flavin@MOF-5 five slices in the center of the z-stack were selected to generate the decay histograms, for flavin@MIL53 all photons from the images were used.

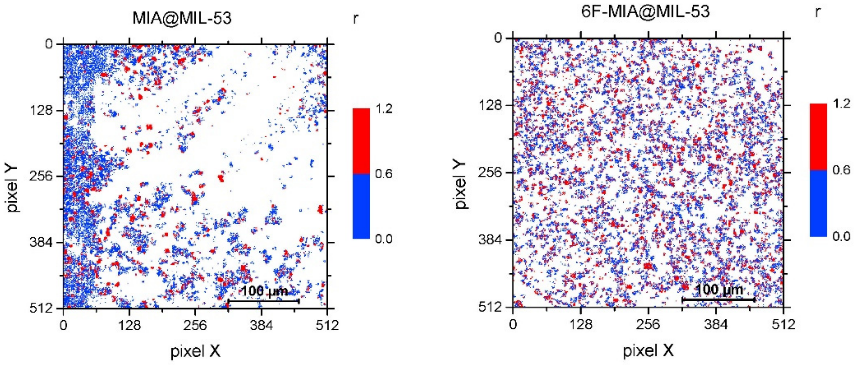

Figure S35. MFIS images of flavin@MIL-53 displaying experimental steady state fluorescence anisotropy *r* of Figure 6 with a distinct color scheme to highlight that one microcrystal has a unique anisotropy, i.e. *r* is a unique feature for each crystal that is related to its orientation. For further information, see discussion of Figure 6 in the main text section 2.3.

Section S6: Theoretical calculations (Figure S36 - Figure S42)

Force Field parameters are in the AMBER20 format and units.

Section S6.1: Force Field MIA

Atom number		*FF atom type*
O	1	Oc
O	2	Oc
N	3	Na
N	4	Nc
N	5	Nc
N	6	N
H	7	H1
H	8	Hn
H	9	Hm
H	10	Hm
H	11	Hm
H	12	Hm
H	13	H1
H	14	H1
C	15	Cd
C	16	Cd
C	17	Co
C	18	Co
C	19	Cm
C	20	Cm
C	21	Cm
C	22	Cm
C	23	Cm
C	24	C3
C	25	Cm

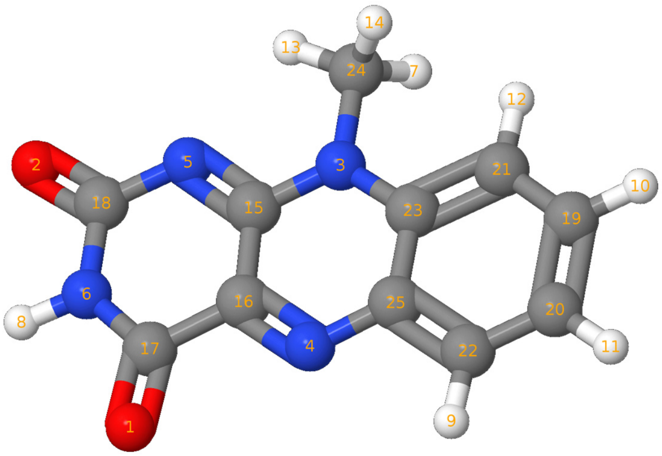

MASS

om	16.000	0.434
n	14.010	0.530
nc	14.010	0.530
na	14.010	0.530
hn	1.008	0.161
h1	1.008	0.135
hm	1.008	0.135
c2	12.010	0.616
cd	12.010	0.360
c3	12.010	0.878
cm	12.010	0.360

BOND STRETCHING

om-c2	637.70	1.218
n -hn	403.20	1.013

n -c2	427.60	1.379
nc-c2	416.90	1.387
nc-cd	525.40	1.317
nc-cm	467.70	1.352
na-cd	425.80	1.380
na-c3	327.70	1.463
na-cm	420.50	1.384
h1-c3	330.60	1.097
hm-cm	345.80	1.086
c2-cd	371.00	1.468
cd-cd	419.80	1.428
cm-cm	461.10	1.398

ANGLE BENDING

om-c2-n	74.220	123.050
om-c2-nc	73.910	123.180
om-c2-cd	69.140	123.930
n -c2-nc	71.580	117.110
n -c2-cd	69.070	112.700
nc-cd-na	74.900	112.220
nc-cd-cd	71.570	112.560
nc-cd-c2	67.620	121.880
nc-cm-cm	69.460	119.720
na-cd-cd	68.580	117.770
na-c3-h1	49.830	108.780
na-cm-cm	69.080	118.340
hn-n -c2	48.330	117.550
h1-c3-h1	39.240	108.460
hm-cm-cm	48.180	119.880
c2-n -c2	63.740	127.080
c2-nc-cd	66.680	120.490
c2-cd-cd	63.620	122.690
cd-nc-cm	72.480	104.880
cd-na-c3	61.880	126.460
cd-na-cm	67.400	113.150
c3-na-cm	62.320	124.360
cm-cm-cm	66.620	120.020

TORSION

om-c2-n -hn	1	2.500	180.000	-2.000
om-c2-n -hn	1	2.000	0.000	1.000
om-c2-n -c2	1	2.500	180.000	2.000
om-c2-nc-cd	1	4.000	180.000	2.000
om-c2-cd-nc	1	2.875	180.000	2.000
om-c2-cd-cd	1	2.875	180.000	2.000
n -c2-nc-cd	1	4.000	180.000	2.000
n -c2-cd-nc	1	2.875	180.000	2.000

n -c2-cd-cd	1	2.875	180.000	2.000
nc-c2-n -hn	1	2.500	180.000	2.000
nc-c2-n -c2	1	2.500	180.000	2.000
nc-cd-na-c3	1	1.700	180.000	2.000
nc-cd-na-cm	1	1.700	180.000	2.000
nc-cd-cd-nc	1	4.000	180.000	2.000
nc-cd-cd-c2	1	4.000	180.000	2.000
nc-cd-cd-na	1	4.000	180.000	2.000
nc-cm-cm-na	1	3.625	180.000	2.000
nc-cm-cm-cm	1	3.625	180.000	2.000
nc-cm-cm-hm	1	3.625	180.000	2.000
na-cd-nc-c2	1	4.750	180.000	2.000
na-cd-cd-c2	1	4.000	180.000	2.000
na-cm-cm-cm	1	3.625	180.000	2.000
na-cm-cm-hm	1	3.625	180.000	2.000
hn-n -c2-cd	1	2.500	180.000	2.000
h1-c3-na-cd	1	0.000	0.000	2.000
h1-c3-na-cm	1	0.000	0.000	2.000
hm-cm-cm-cm	1	3.625	180.000	2.000
hm-cm-cm-hm	1	3.625	180.000	2.000
c2-n -c2-cd	1	2.500	180.000	2.000
c2-nc-cd-cd	1	4.750	180.000	2.000
c2-cd-nc-cm	1	4.750	180.000	2.000
cd-nc-cm-cm	1	4.800	180.000	2.000
cd-cd-na-c3	1	1.700	180.000	2.000
cd-cd-na-cm	1	1.700	180.000	2.000
cd-na-cm-cm	1	0.300	180.000	2.000
cd-cd-nc-cm	1	4.750	180.000	2.000
c3-na-cm-cm	1	0.300	180.000	2.000
cm-cm-cm-cm	1	3.625	180.000	2.000

OUT-OF-PLANE

c2-c2-n -hn	1.1	180.0	2.0
c3-cm-na-cd	1.1	180.0	2.0
n -nc-c2-om	10.5	180.0	2.0
cd-n -c2-om	10.5	180.0	2.0
c2-cd-cd-nc	1.1	180.0	2.0
cd-na-cd-nc	1.1	180.0	2.0
cm-cm-cm-nc	1.1	180.0	2.0
cm-cm-cm-na	1.1	180.0	2.0
cm-cm-cm-hm	1.1	180.0	2.0

NON-BONDED

om	1.6612	0.2100
n	1.8240	0.1700
nc	1.8240	0.1700
na	1.8240	0.1700

hn	0.6000	0.0157
h1	1.3870	0.0157
hm	1.4590	0.0150
c2	1.9080	0.0860
cd	1.9080	0.0860
c3	1.9080	0.1094
cm	1.9080	0.0860

Section S6.2: Force Field MIL-53(Al)

-Metal residue (Al):

Atom number		*FF atom type*
Al	1	Al

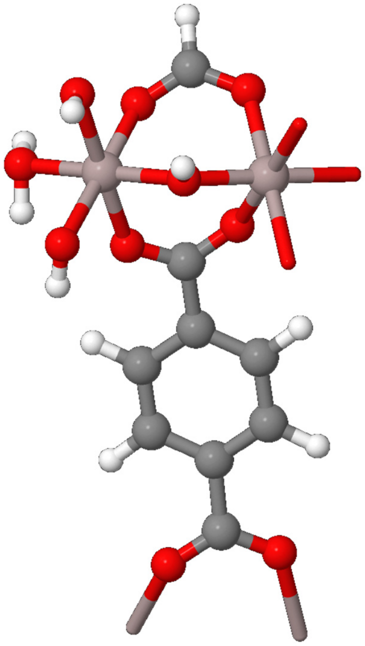

-Linker ($C_8O_4H_4$):

Atom number		*FF atom type*
O	1	Oc
O	2	Oc
O	3	Oc
O	4	Oc
H	5	Ha
H	6	Ha
H	7	Ha
H	8	Ha
C	9	Co
C	10	Cp
C	11	Ca
C	12	Ca
C	13	Ca
C	14	Ca
C	15	Cp
C	16	Co

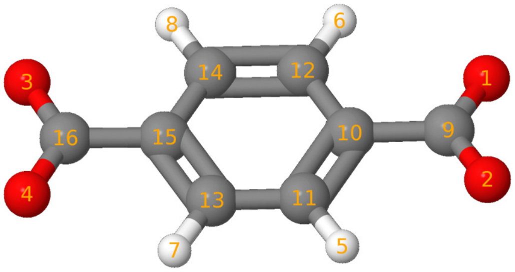

-Hydroxy bridge (OH):

Atom number		*FF atom type*
O	1	Oh
H	2	Ho

-Border residue (CO_2H):

Atom number		*FF atom type*
O	1	Oc
O	2	Oc
H	3	H5
C	4	Co

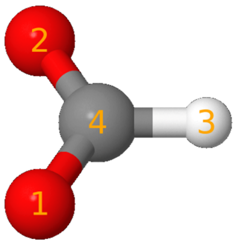

- Border Hydroxy (OH):

Atom number		*FF atom type*
O	1	Oy
H	2	Hy

- Border water (OH_2):

Atom number		*FF atom type*
O	1	Ow
H	2	Hw
H	3	Hw

MASS

al	26.982	0.520
oh	16.000	0.465
oc	16.000	0.465
od	16.000	0.465
ow	16.000	0.465
oy	16.000	0.465
co	12.010	0.360
cp	12.010	0.360
ca	12.010	0.360
ho	1.008	0.135
ha	1.008	0.135
h5	1.008	0.135
hw	1.008	0.135
hy	1.008	0.135

BOND STRETCHING

al-oh	226.383	1.807
al-oc	191.193	1.905
al-od	185.625	1.910
al-oy	179.614	1.802
oh-ho	483.767	0.948
oy-hy	619.790	0.958
oc-co	532.656	1.264
od-co	515.050	1.268
co-cp	323.272	1.482
cp-ca	394.916	1.397
ca-ca	394.916	1.397
ca-ha	271.310	1.200
co-h5	319.400	1.115
al-ow	226.383	1.807

ANGLE BENDING

al-oh-al	267.297	125.491

al-oh-ho	88.822	114.648
al-oy-hy	17.242	122.255
al-oc-co	94.016	133.962
al-od-co	94.016	133.962
oc-al-oc	56.898	179.890
od-al-od	55.952	179.789
oh-al-oh	61.380	179.487
oc-al-od	180.099	91.440
od-al-oh	9.883	90.324
oh-al-oc	221.271	90.942
oy-al-oy	46.500	90.000
oy-al-oh	63.653	90.000
oy-al-oc	63.653	90.000
oy-al-od	63.653	90.000
oy-al-ow	46.500	90.000
oc-co-oc	290.609	124.241
od-co-od	287.813	123.089
oc-co-cp	290.848	117.674
od-co-cp	290.848	117.674
co-cp-ca	284.293	120.197
cp-ca-ca	136.436	120.261
cp-ca-ha	82.144	121.174
ca-cp-ca	136.436	120.261
ca-ca-ha	82.144	121.174
oc-co-h5	55.501	123.760
od-co-h5	55.501	123.760
ow-al-oc	221.271	90.942
ow-al-od	221.271	90.942
ow-al-oh	221.271	90.942
ow-al-ow	55.952	179.789
al-ow-hw	88.822	114.648

TORSION

al-oc-co-cp	1	1.516	180.000	2.000
al-od-co-cp	1	1.516	180.000	2.000
al-oc-co-h5	1	1.516	180.000	2.000
oc-co-cp-ca	1	2.175	180.000	-2.000
oc-co-cp-ca	1	0.300	0.000	3.000
od-co-cp-ca	1	2.175	180.000	-2.000
od-co-cp-ca	1	0.300	0.000	3.000
co-cp-ca-ca	1	6.650	180.000	2.000
co-cp-ca-ha	1	6.650	180.000	2.000
cp-ca-ca-ha	1	6.650	180.000	2.000
ca-cp-ca-ha	1	6.650	180.000	2.000
ha-ca-ca-ha	1	6.650	180.000	2.000

OUT-OF-PLANE

cp-oc-oc-co	1.100	180.0000	2.000
co-ca-ca-cp	1.100	180.0000	2.000
ca-cp-ha-ca	1.100	180.0000	2.000
ca-ca-ca-ha	1.100	180.0000	2.000
co-ca-cp-ca	1.100	180.0000	2.000
ca-ca-cp-co	1.100	180.0000	2.000

NON-BONDED

al	2.36	0.115918
oh	1.82	0.059034
oc	1.82	0.059034
od	1.82	0.059034
oy	1.82	0.059034
ow	1.82	0.059034
co	1.94	0.055927
cp	1.94	0.055927
ca	1.94	0.055927
ho	1.60	0.016013
ha	1.60	0.016013
hw	1.60	0.016013
h5	1.60	0.016013
hy	1.60	0.016013

Section S6.3: Force Field MOF-5

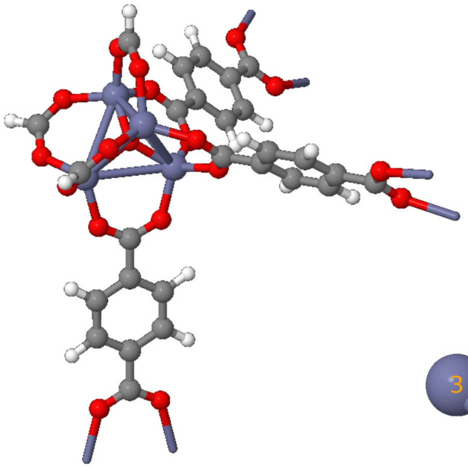

-Metal residue (Zn_4O):

Atom number		*FF atom type*
Zn	1	Zn
Zn	2	Zn
Zn	3	Zn
Zn	4	Zn
O	1	Os

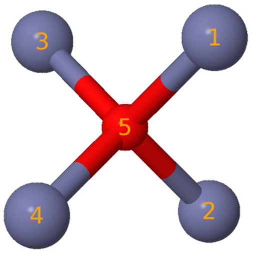

-Linker ($C_8O_4H_4$):

Atom number		*FF atom type*
O	1	O
O	2	O
O	3	O
O	4	O
H	5	Ha
H	6	Ha
H	7	Ha
H	8	Ha
C	9	C
C	10	Ca

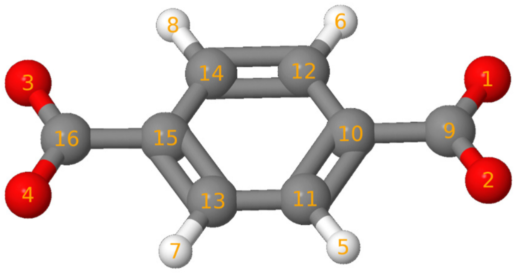

C	11	Ca
C	12	Ca
C	13	Ca
C	14	Ca
C	15	Ca
C	16	C

-Border residue (CO_2H):

Atom number		*FF atom type*
O	1	O
O	2	O
H	3	H5
C	4	C

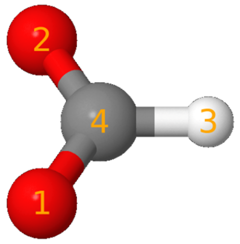

MASS

zn	65.38	0.999
o	16.00	0.434
os	16.00	0.465
c	12.01	0.616
ca	12.01	0.360
ha	1.008	0.135
h5	1.008	0.135

BOND STRETCHING

c -o	637.70000	1.2183
o -zn	61.13030	2.0150
zn-os	61.13030	2.0750
ca-o	542.26172	1.2850
ca-ca	471.78208	1.3800
c -ca	348.08312	1.4800
h5-c	319.40000	1.1150

ANGLE BENDING

zn-os-zn	28.76720	109.471
o -zn-os	21.57540	111.300
o -zn-o	15.82196	107.600
c -o -zn	30.92474	131.200
o -c – o	46.02752	125.500
ca-ca- c	52.50014	120.000
o – c-ca	56.09604	117.000
h5-c -o	55.50125	123.760

TORSION

ca-ca- c- o	1	1.0	180.0	2.0
os-zn- o- c	1	5.0	180.0	2.0

zn-os-zn- o	1	5.0	180.0	2.0
zn- o- c- c	1	5.0	180.0	2.0
zn -o- c-ca	1	5.0	180.0	2.0
zn-o -c -h5	1	1.6	180.0	2.0

OUT-OF-PLANE

o -o -c -ca	16.54	180.0	2.0
ca-ca-ca-ha	1.1	180.0	2.0
c -ca-ca-ca	1.1	180.0	2.0
ca-o -c -o	1.1	180.0	2.0

NON-BONDED

zn	2.29	0.276
os	1.82	0.059
o	1.82	0.059
c	1.94	0.056
ca	1.94	0.056
ha	1.62	0.020

Section S6.4: QM part, MM part

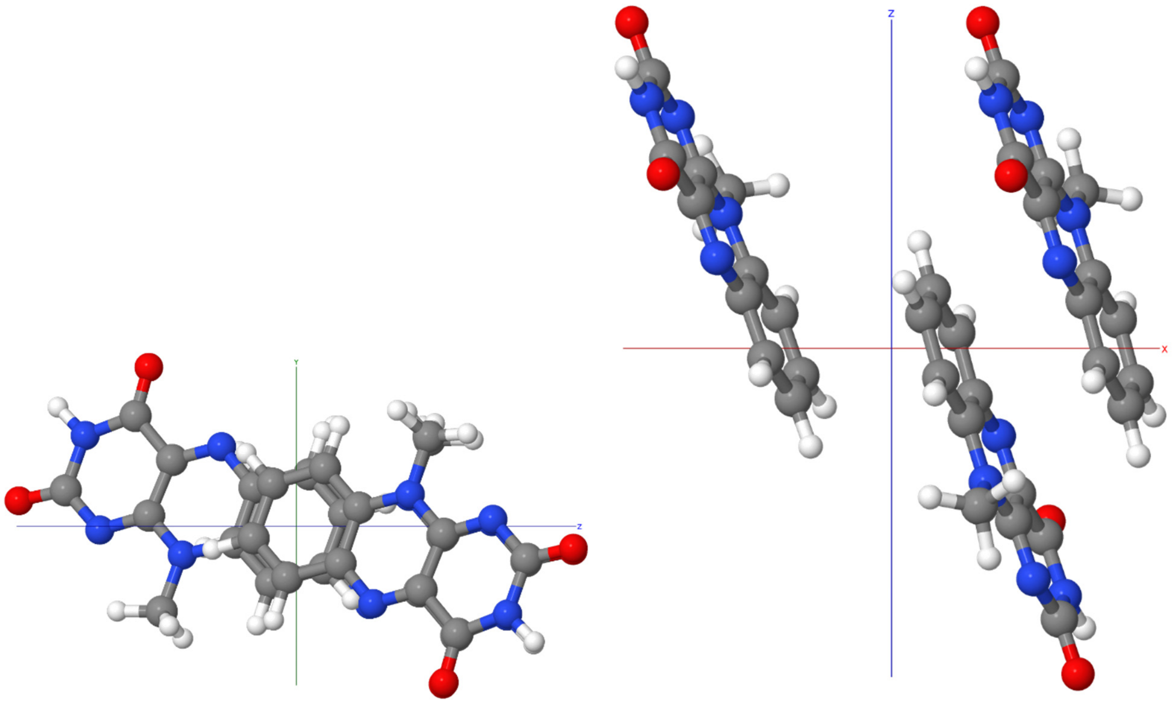

Figure S36. QM-Layer including three MIA molecules. During QM/MM-optimization only QM atoms were allowed to move (mobile layer), the remaining atom position were fixed at their optimized values from the corresponding plane-wave calculation with QuantumEspresso [24]. (left: view along X axis, right: view along Y axis).

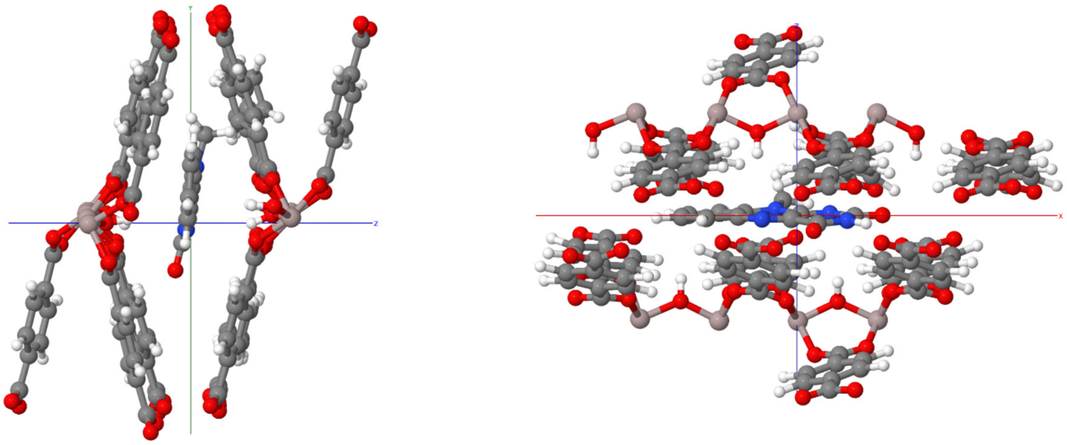

Figure S37. The mobile layer in MIL-53(Al) contains MIA and all residues that have at least 1 atom in a radius of 7.5 Å around the MIA chromophore (QM layer (MIA) and movable MM portion (MOF), left: view along X axis, right: view along Y axis). The remaining atoms (not shown) were fixed at their optimized positions from the plane-wave computation with QuantumEspresso [24].

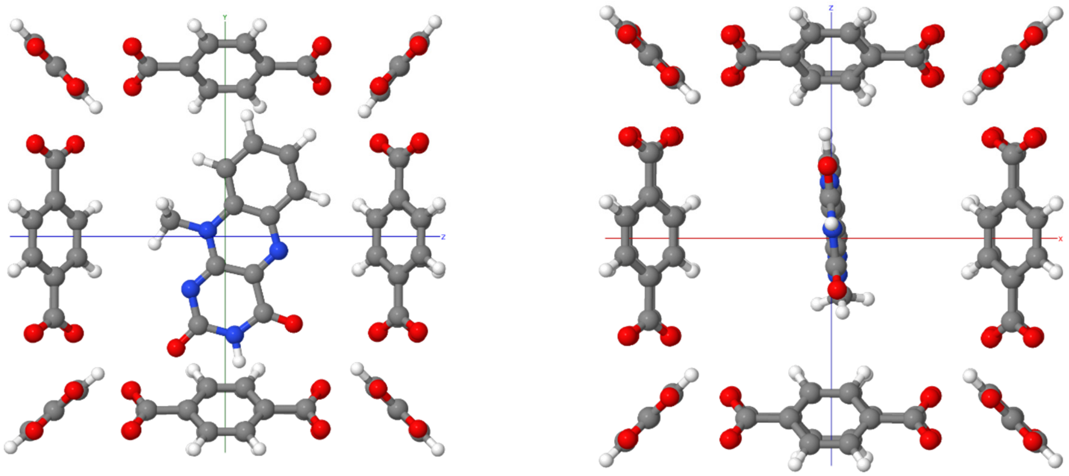

Figure S38. The mobile layer in both *inward* and *outward* pores of MOF-5, containing MIA and all residues that have at least one atom within a radius of 5.0 Å around the MIA chromophore. Zn_4O residues and the remaining portion of the MOF were kept fixed at their plane-wave optimized positions. (QM layer (MIA) and medium layer (MOF), left: view along X axis, right: view along Y axis).

Cluster for MIL-53(Al) Force Field generation:

The same cluster as presented in the work of Vanduyfhuys et al. [25] was used to generate the MIL-53(Al) force field (Figure S39). The total charge was –1. It was first geometry-optimized with the B3LYP hybrid functional and the 6-311++G(d,p) basis set, followed by vibrational analysis using the Gausian16 package [26].

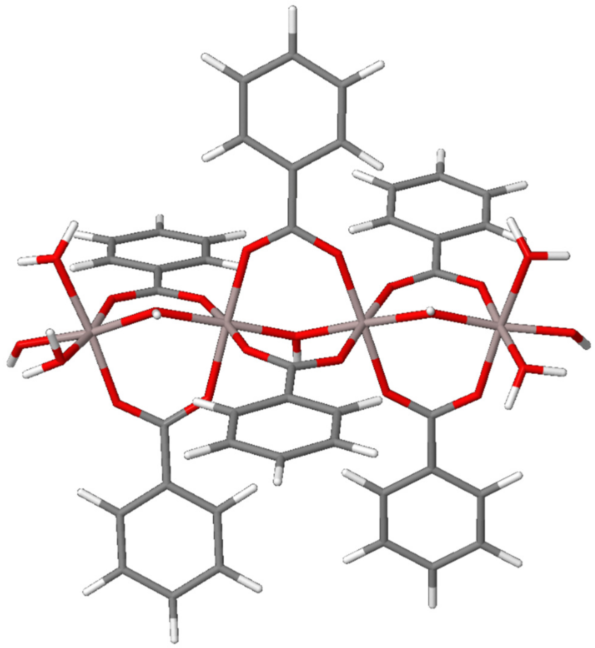

Figure S39. Cluster model of Vanduyfhuys et al. [25] used for generating the MIL-53(Al) force field used in this work.

Table S3. Crystallographic data for MIL-53 phases (powder data with Rietveld refinement from literature) and MIL-53 (computed).

Compound	experiment. MIL-53-lp [a]	computed MIL-53-lp [b]	experiment. MIL-53-np [a,c]	computed MIA@MIL-53 [b]
Crystal system	orthorhombic	unconstrained (triclinic)	monoclinic	unconstrained (triclinic)
Space group	*Imma*	unconstrained (*P*1)	*Cc*	unconstrained (*P*1)
a [Å]	6.6085(9)	6.8904	19.513(2)	20.1698 [d]
b [Å]	16.675(3)	16.4224	7.612(1)	18.8673
c [Å]	12.813(2)	13.5000	6.576(1)	8.9399
α [°]	90	90	90	89.92
β [°]	90	90	104.24(1)	90.31
γ [°]	90	90	90	87.29
V [Å^3]	1411.95(40)	1527.62	946.74(10)	3398.21

[a] CCDC no. 220476 (lp), 220477 (np) [6]. [b] Cell parameters refined through Quantum Espresso. [c] The cell setting/orientation of the coordinate system in the experimental structure of MIL-53-np is different from those of the other three (exp. and comput.) structures. To facilitate a comparison of the axes lengths, matching directions are given in the same color. [d] Note that in computed MIA@MIL-53 the *a* axis (channel direction) was extended to about three times the corresponding axis length in experimental MIL-53-lp or -np (ca. 6.6 Å) to accommodate separated MIA molecules with their length of 12 Å (cf. Figure S12).

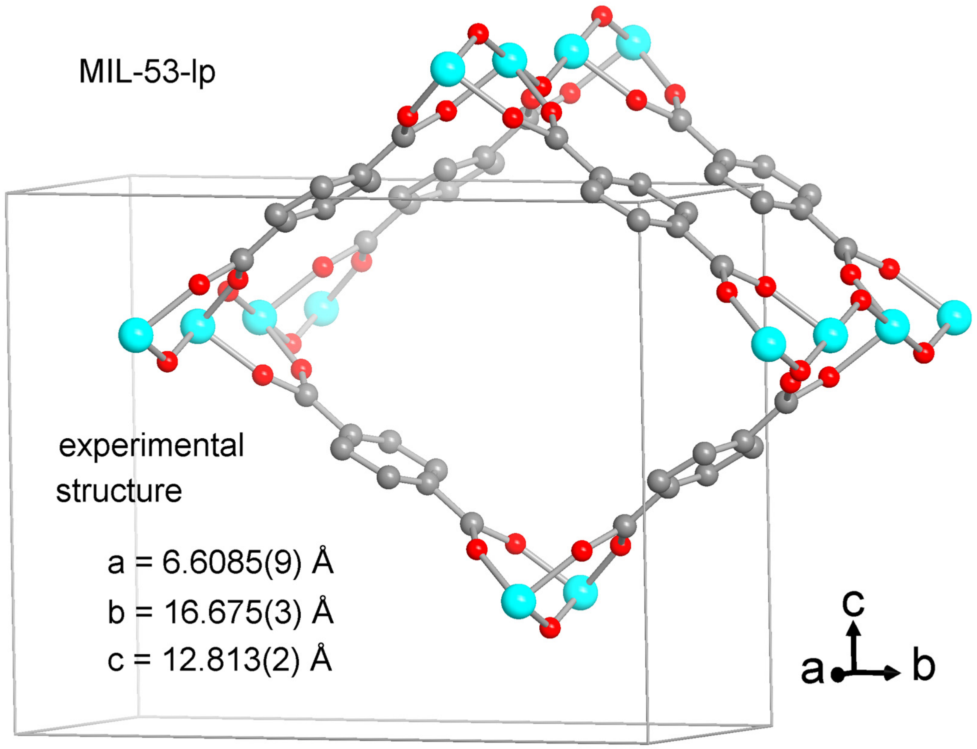

(a)

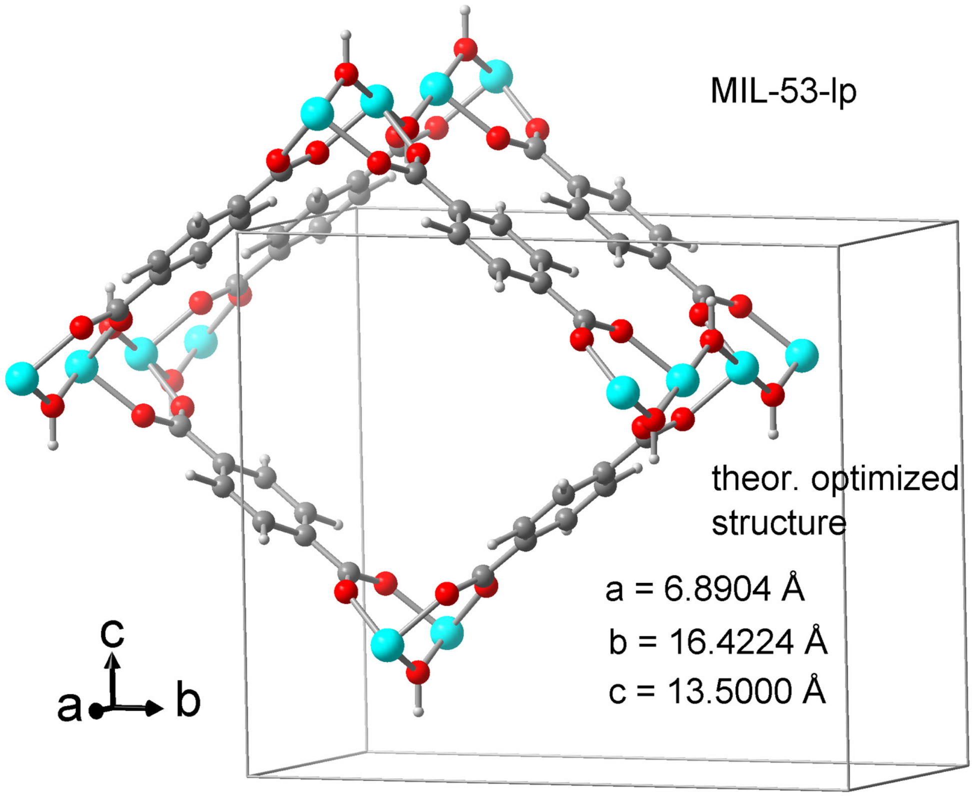

(b)

Figure S40. Sections of the structures with the cell edges and cell constants of MIL-53-lp from (a) experimental Rietveld refinement (CCDC no./Refcode 220476/SABVUN) [6] and (b) from theoretical optimization with QuantumEspresso (see also Table S3). Compared to the experimental orthorhombic large-pore form MIL-53-lp the computed guest-free MIL-53-lp structure after optimization widens by ca. 0.3 Å and 0.7 Å in a- and c-directions, respectively, while it shrinks by ca. 0.25 Å in the b-direction, with the cell angles remaining at 90°

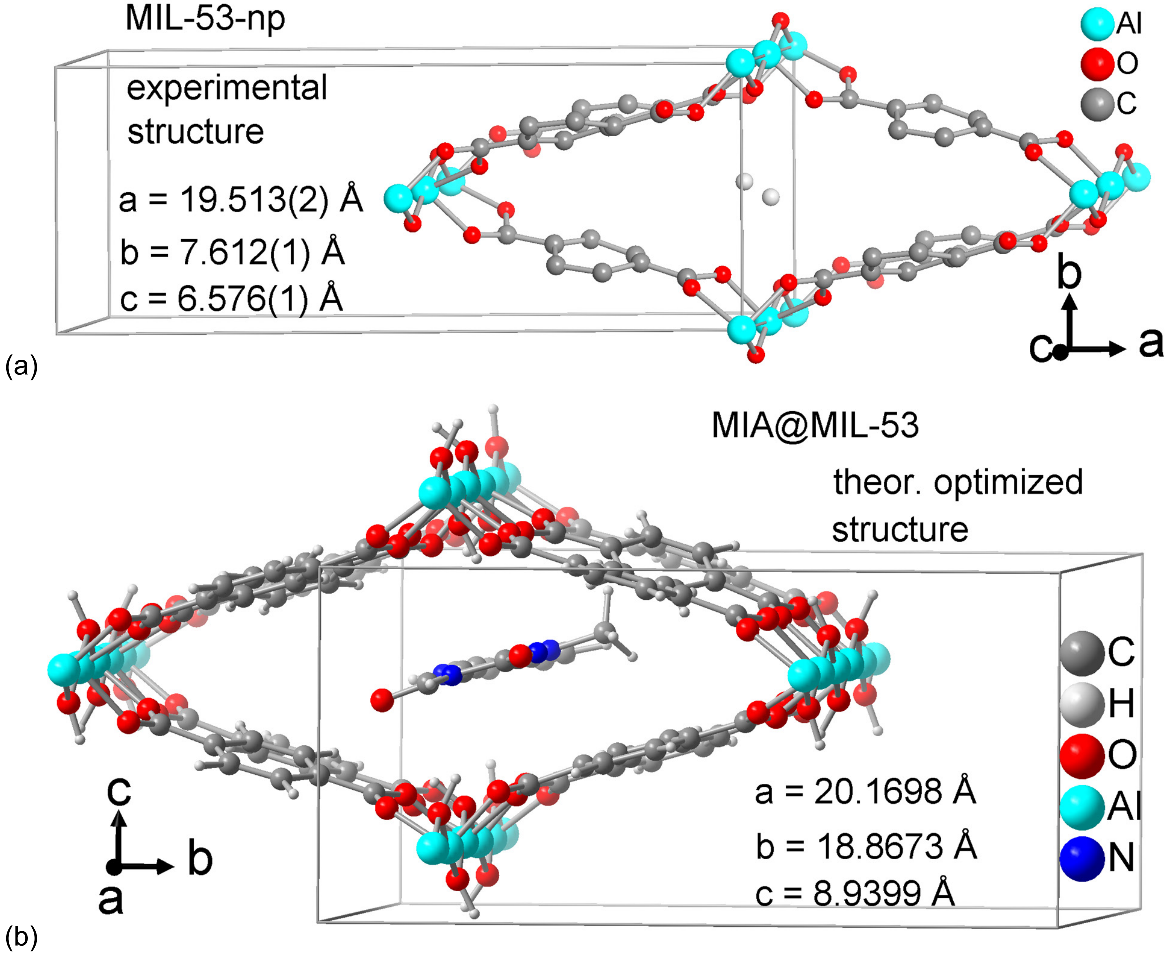

Figure S41. Sections of the structures with the cell edges and cell constants of (a) MIL-53-np from experimental Rietveld refinement (CCDC no./Refcode 220477/SABWAU) [6] and of (b) MIA@MIL-53 from theoretical optimization with QuantumEspresso. The theoretical cell setting/orientation of the coordinate system in (b) is the same as in the MIL-53-lp structures in Figure S40 but different to the MIL-53-np structure in (a). However, the axis parallel to the channel direction in MIA@MIL-53 (*a* axis, ca. 20 Å) was extended to about three times the corresponding axis length in experimental MIL-53-lp or -np (ca. 6.6 Å) to accommodate separated MIA molecules in the channels (cf. MIA length of 12 Å in Figure S12) (see also Table S3).

Section S6.5: Transition dipole moments of MIA

The orientations of the transition dipole moment vector (TDM) were obtained from the quantum-chemical calculations for S0-> S1 (absorption) and for S1-> S0 (fluorescence) (see example in Figure S42). The vector coordinates are as follows:

MIA in vacuum			
TDM	x	y	z
Absorption (S0-S1)	1.8176223	-0.4546537	0.7971484
Emission (S1-S0)	1.6719799	-0.3554352	0.7142668
Difference (S0-S1)	0.1456424	-0.0992185	0.0828816
Angle = 1.9°			

MIA@MIL-53			
TDM	x	y	z
Absorption (S0-S1)	1.7076416	0.9172188	-0.1371604
Emission (S1-S0)	1.5876942	-0.9185493	-0.1183539
Difference (S0-S1)	0.1199474	0.0013305	-0.0188065
Angle = 1.8°			

MIA@MOF-5			
TDM	x	y	z
Absorption (S0-S1)	0.0462862	-2.0319814	-0.0827332
Emission (S1-S0)	-0.0345843	-1.8358583	-0.1126006
Difference (S0-S1)	0.0808705	-0.1961231	
Angle = 2.7°			

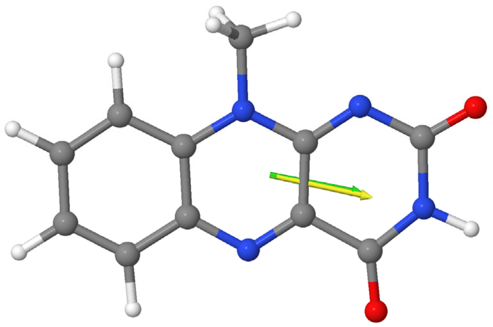

Figure S42. Transition dipole moments of MIA@MIL-53. Yellow arrow for $S_0 \rightarrow S_1$ (absorption) and green arrow for $S_1 \rightarrow S_0$ (fluorescence).

References

1 Brown, S.A.; Rizzo, C.J. A “One-Pot” Phase Transfer Alkylation/Hydrolysis of *o*-Nitrotrifluoroacetanilides. A Convenient Route to *N*-Alkyl o-Phenylenediamines. *Synth. Commun.* **1996**, *26*, 4065-4080. https://doi.org/10.1080/00397919608003827

2 Imada, Y.; Iida, H.; Ono, S.; Masui, Y.; Murahashi, S.-I. Flavin-Catalyzed Oxidation of Amines and Sulfides with Molecular Oxygen: Biomimetic Green Oxidation. *Chem. Asian J.* **2006**, 136-147. https://doi.org/10.1002/asia.200600080

3 Reiffers, A.; Ziegenbein, C.T.; Engelhardt, A.; Kühnemuth, R.; Gilch, P.; Czekelius, C. Impact of Mono-Fluorination on the Photophysics of the Flavin Chromophore. *Photochem. Photobiol.* **2018**, *94*, 667-676. https://doi.org/10.1111/php.12921

4 Kumar, V.; Bode, K.A.; Bryan, R.F.; Averill, B.A. Evidence for a competing condensation reaction in the alloxan synthesis of flavins: synthesis and crystal and molecular structures of 7-chloro-8-methylalloxazine and 7,10-dimethyl-8-[(2-hydroxyethyl)thio]isoalloxazine. *J. Am. Chem. Soc.* **1986**, *108*, 490-496. https://doi.org/10.1021/ja00263a022

5 Alaerts, L.; Maes, M.; Giebeler, L.; Jacobs, P.A.; Martens, J.A.; Denayer, J.F.M.; Kirschhock, C.E.A.; De Vos, D.E. Selective Adsorption and Separation of ortho-Substituted Alkylaromatics with the Microporous Aluminum Terephthalate MIL-53. *J. Am. Chem. Soc.* **2008**, *130*, 14170-14178. https://doi.org/10.1021/ja802761z

6 Loiseau, T.; Serre, C.; Huguenard, C.; Fink, G.; Taulelle, F.; Henry, M.; Bataille, T.; Férey, G. A Rationale for the Large Breathing of the Porous Aluminum Terephthalate (MIL-53) Upon Hydration *Chem. Eur. J.* **2004**, *10*, 1373-1382. https://doi.org/10.1002/chem.200305413

7 Han, S.; Wei, Y.; Valente, C.; Lagzi, I.; Gassensmith, J.J.; Coskun, A.; Stoddart, J.F.; Grzybowski, B.A. Chromatography in a Single Metal−Organic Framework (MOF) Crystal. *J. Am. Chem. Soc.* **2010**, *132*, 16358-16361. https://doi.org/10.1021/ja1074322

8 Eddaoudi, M.; Kim, J.; Rosi, N.; Vodak, D.; Wachter, J.; O'Keeffe, M.; Yaghi, O.M. Systematic Design of Pore Size and Functionality in Isoreticular MOFs and Their Application in Methane Storage. *Science* **2002**, *295*, 469-472. https://doi.org/10.1126/science.1067208

9 Brandenburg, K. *Diamond (Version 4.6), Crystal and Molecular Structure Visualization*, *Crystal Impact* – K. Brandenburg & H. Putz Gbr, Bonn, Germany, **2009-2022**.

10 Wang, M., Fritchie Jr., C.J. Geometry of the Unperturbed Flavin Nucleus. The Crystal Structure of 10-Methylisoalloxazine. *Acta Cryst.* **1973**, *B29*, 2040-2045. https://doi.org/10.1107/S0567740873006096

11 Meilikhov, M.; Yusenko, K.; Fischer, R.A. The adsorbate structure of ferrocene inside [Al(OH)(bdc)]x (MIL-53): a powder X-ray diffraction study. *Dalton Trans.* **2009**, 600-602. https://doi.org/10.1039/B820882B

12 Couck, S.; Gobechiya, E.; Kirschhock, C.E.A.; Serra-Crespo, P.; Juan-Alcañiz, J.; Martinez Joaristi, A.; Stavitski, E.; Gascon, J.; Kapteijn, F.; Baron, G.V.; Denayer, J.F.M. Adsorption and Separation of Light Gases on an Amino-Functionalized Metal–Organic Framework: An

Adsorption and In Situ XRD Study. *ChemSusChem*, **2012**, *5*, 740-750. https://doi.org/10.1002/cssc.201100378

13 Rosenbach, N.J.; Jobic, H.; Ghoufi, A.; Salles, F.; Maurin, G.; Bourrelly, S.; Llewellyn, P.L.; Devic, T.; Serre, C.; Férey, G. Quasi-Elastic Neutron Scattering and Molecular Dynamics Study of Methane Diffusion in Metal Organic Frameworks MIL-47(V) and MIL-53(Cr). *Angew. Chem. Int. Ed.* **2008**, *47*, 6611-6615. https://doi.org/10.1002/anie.200801748

14 Salles, F.; Bourrelly, S.; Jobic, H.; Devic, T.; Guillerm, V.; Llewellyn, P.; Serre, C.; Ferey, G.; Maurin, G. Molecular Insight into the Adsorption and Diffusion of Water in the Versatile Hydrophilic/Hydrophobic Flexible MIL-53(Cr) MOF. *J. Phys. Chem. C* **2011**, 115, 21, 10764-10776. https://doi.org/10.1021/jp202147m

15 Bauer, S.; Serre, C.; Devic, T.; Horcajada, P.; Marrot, J.; Férey, G.; Stock, N. High-Throughput Assisted Rationalization of the Formation of Metal Organic Frameworks in the Iron(III) Aminoterephthalate Solvothermal System, *Inorg. Chem.* **2008**, *47*, 7568-7576. https://doi.org/10.1021/ic800538r

16 Serre, C.; Millange, F.; Thouvenot, C.; Noguès, M.; Marsolier, G.; Louër, D.; Férey, G. Very Large Breathing Effect in the First Nanoporous Chromium(III)-Based Solids: MIL-53 or $Cr^{III}(OH)\cdot\{O_2C-C_6H_4-CO_2\}\cdot\{HO_2C-C_6H_4-CO_2H\}_x\cdot H_2O_y$. *J. Am. Chem. Soc.* **2002**, *124*, 13519-13526. https://doi.org/10.1021/ja0276974

17 Millange, F.; Serre, C.; Férey, G. Synthesis, structure determination and properties of MIL-53as and MIL-53ht: the first Cr^{III} hybrid inorganic–organic microporous solids: $Cr^{III}(OH)\cdot\{O_2C-C_6H_4-CO_2\}\cdot\{HO_2C-C_6H_4-CO_2H\}_x$. *Chem. Commun.* **2002**, 822-823. https://doi.org/10.1039/B201381A

18 Millange, F.; Guillou, N.; Walton, R.I.; Grenèche, J.-M.; Margiolaki, I.; Férey, G. Effect of the nature of the metal on the breathing steps in MOFs with dynamic frameworks. *Chem. Commun.* **2008**, 4732-4734. https://doi.org/10.1039/B809419E

19 Li, H.; Eddaoudi, M.; O'Keeffe, M.; Yaghi, O.M. Design and synthesis of an exceptionally stable and highly porous metal-organic framework. *Nature* **1999**, *402*, 276-279. https://doi.org/10.1038/46248

20 Li, H.; Eddaoudi, M.; O'Keeffe, M.; Yaghi, O.M. *CCDC 256966: Experimental Crystal Structure Determination*, Cambridge Crystallographic Data Centre, **2005**. https://doi.org/10.5517/cc8md7h

21 Bailey, M.; Brown, C.J. The crystal structure of terephthalic acid. *Acta Crystallogr.* **1967**, *22*, 387-391. https://doi.org/10.1107/s0365110x67000751

22 Lock, N.; Wu, Y.; Christensen, M.; Cameron, L.J.; Peterson, V.K.; Bridgeman, A.J.; Kepert, C.J.; Iversen, B.B. Elucidating Negative Thermal Expansion in MOF-5. *J. Phys. Chem. C* **2010**, *114*, 16181-16186. https://doi.org/10.1021/jp103212z

23 Möckel, C.; Kubiak, J.; Schillinger, O.; Kühnemuth, R.; Della Corte, D.; Schröder, G.F.; Willbold, D.; Strodel, B.; Seidel, C.A.M.; Neudecker, P. Integrated NMR, fluorescence and MD benchmark

study of protein mechanics and hydrodynamics. *J. Phys. Chem. B* **2019**, *123*, 1453-1480. https://doi.org/10.1021/acs.jpcb.8b08903

24 Giannozzi, P.; Baroni, S.; Bonini, N.; Calandra, M.; Car, R.; Cavazzoni, C.; Ceresoli, D.; Chiarotti, G.L.; Cococcioni, M.; Dabo, I.; Dal Corso, A.; de Gironcoli, S.; Fabris, S.; Fratesi, G.; Gebauer, R.; Gerstmann, U.; Gougoussis, C.; Kokalj, A.; Lazzeri, M.; Martin-Samos, L.; Marzari, N.; Mauri, F.; Mazzarello, R.; Paolini, S.; Pasquarello, A.; Paulatto, L.; Sbraccia, C.; Scandolo, S.; Sclauzero, G.; Seitsonen, A.P.; Smogunov, A.; Umari, P.; Wentzcovitch, R.M. QUANTUM ESPRESSO: a modular and open-source software project for quantum simulations of materials. *J. Phys.: Condens. Matter* **2009**, *21*, 395502. https://doi.org/10.1088/0953-8984/21/39/395502

25 Vanduyfhuys, L.; Vandenbrande, S.; Verstraelen, T.; Schmid, R.; Waroquier, M.; Van Speybroeck, V. QuickFF: A program for a quick and easy derivation of force fields for metal-organic frameworks from ab initio input. *J. Comput. Chem.* **2015**, *36*, 1015-1027. https://doi.org/10.1002/jcc.23877

26 Frisch, M.J.; Trucks, G.W.; Schlegel, H.B.; Scuseria, G.E.; Robb, M.A.; Cheeseman, J.R.; Scalmani, G.; Barone, V.; Petersson, G.A.; Nakatsuji, H.; et al. *Gaussian 16*, Revision C.01; Gaussian, Inc.: Wallingford, CT, USA, 2016.
